# Australian Guideline for the Identification and Management of Hypertension in Children and Adolescents

**DOI:** 10.1111/jpc.70491

**Published:** 2026-07-14

**Authors:** N. G. Larkins, T. Medley, A. Black, K. A. Bolton, S. Chaturvedi, M. Clark, G. M. Gabb, J. P. Glenning, C. Grimes, R. C. Huang, A. Hyland, C. Jeffries‐Stokes, L. Kalman, S. Kaul, S. Kim, S. McTaggart, R. Kowalski, S. X. Li, S. McNab, D. Metz, A. S. Mihailidou, J. Monin, M. Nelson, M. Pathak, D. Roebuck, M. P. Schlaich, R. Sleaby, C. Quinlan, J. P. Mynard

**Affiliations:** ^1^ Department of Nephrology and Hypertension Perth Children's Hospital Nedlands Western Australia Australia; ^2^ Medical School University of Western Australia Perth Western Australia Australia; ^3^ Nutrition and Health Innovation Research Institute, School of Medical and Health Sciences Edith Cowan University Perth Western Australia Australia; ^4^ Murdoch Children's Research Institute Parkville Victoria Australia; ^5^ Consumer Representative Perth Western Australia Australia; ^6^ Institute for Physical Activity and Nutrition, School of Exercise and Nutrition Sciences, Deakin University Geelong Victoria Australia; ^7^ Department of Nephrology Sydney Children's Hospital Sydney New South Wales Australia; ^8^ Department of General Practice and Primary Care University of Melbourne Parkville Victoria Australia; ^9^ College of Medicine and Public Health Flinders University Adelaide South Australia Australia; ^10^ Cardiology Department Southern Adelaide Local Health Network Adelaide South Australia Australia; ^11^ Acute and Urgent Care Central Adelaide Local Health Network Adelaide South Australia Australia; ^12^ Heart Research Murdoch Children's Research Institute Parkville Victoria Australia; ^13^ Department of Paediatrics University of Melbourne Parkville Victoria Australia; ^14^ Telethon Kids Institute Nedlands Western Australia Australia; ^15^ Department of Nutrition and Dietetics Perth Children's Hospital Nedlands Western Australia Australia; ^16^ Rural Clinical School University of Western Australia Perth Western Australia Australia; ^17^ National Heart Foundation Melbourne Australia; ^18^ Department of Nephrology The Royal Children's Hospital Parkville Victoria Australia; ^19^ Department of Nephrology Monash Children's Hospital Clayton Victoria Australia; ^20^ Department of Nephrology The Children's Hospital at Westmead Sydney New South Wales Australia; ^21^ Department of Nephrology Children's Health Queensland Queensland Australia; ^22^ Department of Cardiology The Royal Children's Hospital Parkville Victoria Australia; ^23^ Department of General Medicine The Royal Children's Hospital Parkville Victoria Australia; ^24^ Department of Cardiology Royal North Shore Hospital Sydney New South Wales Australia; ^25^ Cardiovascular and Hormonal Research Laboratory Kolling Institute St Leonards New South Wales Australia; ^26^ Department of General Medicine The Children's Hospital at Westmead Westmead New South Wales Australia; ^27^ School of Medicine and Menzies Institute for Medical Research University of Tasmania Hobart Tasmania Australia; ^28^ Department of Epidemiology and Preventive Medicine Monash University Clayton Victoria Australia; ^29^ Department of Medical Imaging Perth Children's Hospital Nedlands Western Australia Australia; ^30^ Medical School, Royal Perth Hospital Unit and RPH Research Foundation The University of Western Australia Perth Western Australia Australia; ^31^ Department of Biomedical Engineering University of Melbourne Parkville Victoria Australia

**Abbreviations**
AAP: American Academy of Pediatrics
ABPM: ambulatory blood pressure monitoring
ACEi: angiotensin converting enzyme inhibitor
AGREE: Appraisal of Guidelines for Research and Evaluation
ARB: angiotensin receptor blocker
BP: blood pressure
CAP: Clinical Advisory Panel
CKD: chronic kidney disease
CT: computed tomography
DASH: dietary approaches to stop hypertension
ESCAPE: effect of strict blood pressure control and angiotensin converting enzyme inhibition on the progression of chronic renal failure in pediatric patients
ESH: European Society of Hypertension
GSC: Guideline Steering Committee
HBP: home blood pressure
HOT‐KID: hypertension optimal treatment in children with chronic kidney disease
LVH: left ventricular hypertrophy
LVMI: left ventricular mass index
MRI: magnetic resonance imaging
RCT: randomised controlled trial
SPRINT: systolic blood pressure intervention trial
USPSTF: United States Preventative Services Task Force

## 1 Introduction

Hypertension is among the most important risk factors for chronic disease and avoidable death worldwide [1]. There is now clear evidence that high blood pressure (BP) should be approached from a life‐course perspective, with the identification of early risk factors in time creating opportunity to alter adverse trajectories through early intervention [2]. In recent years, evidence linking childhood BP to clinical events in later life has expanded, building on prior evidence demonstrating an association between BP in children and surrogate endpoints by mid‐adulthood [3, 4, 5, 6, 7, 8, 9, 10]. This is timely because of the increasing prevalence of hypertension among children and adolescents, concurrent with increasing overweight and obesity [11, 12]. These trends are equally apparent among the Australian population with the additional observation of a greater prevalence among Aboriginal and Torres Strait Islander children and adolescents consistent with the marked excess of early cardiac and kidney events in young Aboriginal and Torres Strait Islander adults [13, 14, 15, 16, 17]. Yet, in Australia there is no unified guideline for the identification and management of hypertension for children and adolescents less than 18 years of age [18]. Use of international guidelines may not account for the unique characteristics of the Australian population and healthcare system, including geographic distribution and limited person‐level preventative healthcare mechanisms once children commence school. This guideline, facilitated by the BPOzKids Network and Hypertension Australia, aims to address this gap by providing a unified and practical guideline for the identification and management of hypertension in children and adolescents. It is aligned in all practical aspects to Heart Foundation, Stroke Foundation, and Hypertension Australia guidelines for blood pressure and cardiovascular disease risk [19, 20, 21]. Noting that the Heart Foundation Guideline is due for review shortly and amendments will be issued to this guideline if significant changes are required to maintain continuity.

The following organisations provided input during guideline development and endorsed the final version as published: National Heart Foundation of Australia, Hypertension Australia, Royal Australasian College of Physicians, Cardiac Society of Australia and New Zealand, Kidney Health Australia, Australia and New Zealand Paediatric Nephrology Association, and the Australia New Zealand Paediatric Obesity Network.

## 2 Methods

The Australian Guideline for the Identification and Management of Hypertension in Children and Adolescents (hereafter referred to as the ‘Australian Guideline’) was developed by adapting existing international guidelines to the Australian context using the ADAPTE process [22]. A multidisciplinary Guideline Steering Committee (GSC) oversaw the process and included subject‐matter experts, consumer representation and clinicians experienced in serving rural/remote and First Nations communities. The GSC established the guideline scope as clinical diagnosis and management of hypertension in the Australian population up to 18 years of age. A Clinical Advisory Panel (CAP) was formed to undertake the guideline adaptation and vote on recommendations using a modified Delphi process (Supporting Information Section 3.3.5). The GSC/CAP together developed an implementation plan throughout the guideline development process. In addition, the GSC (1) critically reviewed the guideline developed by the CAP, (2) engaged with consumers and other stakeholders, and (3) oversaw the external consultation, endorsement and dissemination process. Committee membership details are contained in Supporting Information Section 7.

### 2.1 Rationale for the Choice of Guideline for Adaptation

The CAP began by evaluating existing guidelines using Appraisal of Guidelines for Research and Evaluation (AGREE II; Table A1), including the European Society of Hypertension (ESH) 2023 and American Academy of Pediatrics 2017 guidelines (hereafter referred to as AAP Guideline); noting that ESH 2023 is primarily an adult‐focused guideline and mirrors the ESH 2016 guidelines for the management of high blood pressure in children and adolescents which was referred to when relevant [23, 24, 25]. Other guidelines such as the Hypertension Canada 2020 Guideline and Japanese Society of Hypertension Guideline 2019 also have paediatric sections [26, 27]. For the first iteration of the Australian Guideline addressing blood pressure in children and adolescents, it was important to construct a paediatric‐specific document and set of practice principles to guide clinicians, including general practitioners and other health care professionals who may be less familiar with the definitions, investigations, and management options unique to children and adolescents. The AAP Guideline was adapted because the process and available documentation better met the AGREE II quality metric. The AAP Guideline is based on a well described systematic review supported by publicly available evidence to recommendation tables [28]. At a practical level, there are many similarities between the ESH and AAP Guidelines. A comparison of the new Australian guideline with international guidelines is provided in Supporting Information Section 9.

## 3 Summary of the Guideline Adaptation and Key Action Statements

A summary of key principles is presented in Table 1, and the final Key Action Statements (KAS) adapted from the AAP Guideline are presented in Table 2. Table A3 contains the AAP KAS that were not included in the Australian guideline and the reasons why. The next section of this document outlines the main evidence and clinical rationale underpinning these recommendations in sequence according to Table 2.

**TABLE 1:** Summary of key principles from the Australian guideline.

Most hypertension in children is essential (primary), but young children (< 6 years), those with stage 2 hypertension or target organ damage require more detailed investigation for secondary causes.
BP screening for all children is recommended twice during childhood in those without other risk factors, at approximately 7 to 9 and 13 to 15 years of age.
The diagnostic and treatment initiation thresholds are similar to adult populations, after adjustment for growth (95th percentile based on age, sex and height, or ≥ 140/90 mmHg).
Simplified thresholds may be used for initial screening: 120/80 mmHg for children aged 6 to 11 years and 130/85 mmHg for children aged 12 to 17 years.
Out of office BP monitoring is an important component of care. ABPM should be performed prior to treatment^a^ to phenotype BP and exclude white coat hypertension. Out of office blood pressure is more accurate than clinic blood pressure in determining ongoing treatment.
A 6‐month trial of non‐pharmacologic, team‐based management is the optimal first‐line treatment for most children with hypertension.
For children who require antihypertensive medications, the preferred medications are a long‐acting calcium channel blocker or ACEi/ARB.^b^ When combination treatment is required, then a diuretic (e.g., thiazide) should be included as either the 2nd or 3rd agent introduced.
BP should be adequately, rather than just, controlled. The treatment target for most children is < 90th percentile or < 130/80 mmHg. The treatment target for children with chronic kidney disease is < 50th percentile.

Abbreviations: ABPM, ambulatory blood pressure monitoring; ACEi, angiotensin converting enzyme inhibitor; ARB, angiotensin receptor blocker; BP, blood pressure.

^a^ In children able to tolerate the procedure for whom appropriate reference values are available; ≥ 5 years of age or ≥ 120 cm tall.

^b^ Accounting for any specific indications or contraindications (e.g., teratogenicity of ACEi/ARB).

**TABLE 2:** Adapted Australian guideline for the identification and management of hypertension in Australian children diagnosis.

	Key action statements—adapted from the American Academy of Pediatrics 2017	Relationship to original	Certainty of evidence	Strength of recommendation
Diagnosis	1. In children and adolescents without risk factors, assess blood pressure (BP) twice during childhood at approximately 7 to 9 years of age, and again at approximately 13 to 15 years of age. BP may be assessed opportunistically at other ages.	Adapted	Low	Moderate
	2. In all children and adolescents ≥ 3 years of age with risk factors (Table 3), or who are taking medications known to increase BP, assess BP at every health care encounter.	Adopted^d^	Low	Moderate
	3. Diagnosis of hypertension in a child or adolescent should be confirmed with auscultatory BP readings ≥ 95th percentile or ≥ 140/90 mmHg (whichever is lower) at 3 different visits by a trained health care professional. Simplified thresholds may be used for screening in primary care: 120/80 mmHg for children aged 6 to 11 years and 130/85 mmHg for children aged 12 to 17 years.	Adapted	Low	Moderate
	4. Organisations with electronic health records used in a clinic setting should consider including flags for abnormal BP values, both when the values are being entered and when they are being viewed.	Adopted	Low	Weak
	5. BP screening in children and adolescents may be performed with automated oscillometric devices that have been validated in the paediatric age group. If elevated BP is suspected based on oscillometric readings, confirmatory measurements should be obtained by auscultation.	Adopted^d^	Low^a^	Strong
	6. In children and adolescents with clinic BP measurements in the high‐normal BP category for 1 year or more or with stage 1 hypertension over 3 clinic visits, confirm diagnosis with ambulatory BP monitoring.	Adopted^d^	Low	Moderate
	7. In children and adolescents with high‐risk conditions (Table 3), routine ambulatory BP monitoring should be strongly considered to assess for hypertension and determine if abnormal circadian BP patterns are present, which may indicate increased risk for target organ damage.	Adopted^d^	Low	Moderate
	8. When performing ambulatory BP monitoring, use a standardised approach with monitors that have been validated in a paediatric population. Interpret results using paediatric normative data.	Adopted^d^	Low	Moderate
	9. Home BP monitoring should not be used to diagnose hypertension, but may be a useful adjunct to clinic and ambulatory BP measurement after hypertension has been diagnosed.	Adapted	Low	Moderate
Investigation	10. Children and adolescents ≥ 6 years of age with a positive family history of primary hypertension or overweight or obesity, and no history or physical examination findings suggestive of a secondary cause of hypertension do not require an extensive evaluation for secondary causes of hypertension (Tables 7 and 8).	Adopted^d^	Low	Moderate
	11. For children and adolescents who have undergone coarctation repair, use ambulatory BP monitoring to detect hypertension (including masked hypertension).	Adopted^d^	Moderate	Strong
	12. In children and adolescents being evaluated for high BP, the provider should obtain a perinatal history, appropriate nutritional history, physical activity history, psychosocial history, and family history and perform a physical examination to identify findings suggestive of secondary causes of hypertension.	Adopted	N/A	Strong
	13. In hypertensive children and adolescents, do not use electrocardiography to evaluate for left ventricular hypertrophy.	Adopted^d^	Moderate	Strong
	14. When considering pharmacologic treatment of hypertension, perform echocardiography to assess for cardiac target organ damage (left ventricular mass, geometry, and function).	Adopted^d^	Low	Moderate
	15. In the case of persistent hypertension despite treatment, left ventricular hypertrophy, or reduced left ventricular ejection fraction, repeat echocardiography may be performed to monitor improvement or progression of target organ damage at 6‐ to 12‐month intervals.	Adopted^d^	Low	Moderate
	16. In patients with stage 2 hypertension, secondary hypertension, or chronic stage 1 hypertension incompletely treated (noncompliance or drug resistance), and without left ventricle target organ injury at initial echocardiographic assessment, consider repeat echocardiography at yearly intervals to assess for the development of worsening left ventricular target organ injury.	Adopted^d^	Low	Moderate
	17. In normal‐weight children and adolescents ≥ 8 years of age, use Doppler renal ultrasonography as a non‐invasive screening study for the initial evaluation of possible renal artery stenosis.	Adapted	Low	Moderate
	18. In children and adolescents suspected of having renal artery stenosis, direct angiography is the preferred diagnostic test.	Adapted	Low^a^	Moderate^c^
Management	19. In children and adolescents diagnosed with hypertension, the treatment goal with lifestyle and pharmacologic therapy should be a reduction in SBP and DBP to < 90th percentile.	Adapted	Low	Moderate
	20. At the time of diagnosis of high‐normal BP or hypertension in a child or adolescent, provide advice on the DASH diet and recommend moderate to vigorous physical activity at least 3 to 5 days per week (30–60 min per session) to help reduce BP^b^.	Adopted^d^	Low	Weak
	21. In patients with primary hypertension meeting criteria for anti‐hypertensive therapy (Table 10), either ACEi/ARBs or a long‐acting calcium channel blocker are the preferred initial agent. When combination treatment is required, then a diuretic (e.g., thiazide) should be included as either the 2nd or 3rd line agent introduced. The decision on which agent to use should account for specific clinical indications (e.g., ACEi/ARB with diabetes mellitus) or contraindications (e.g., pregnancy and ACEi/ARB, or impaired glucose tolerance and thiazide [relative]). In the absence of these, any of the above can be chosen in discussion with the family.	Adapted	Low^a^	Moderate
	22. In children and adolescents being treated for hypertension, ambulatory BP monitoring may be used to assess treatment effectiveness, especially when clinic and/or home BP measurements indicate insufficient BP response to treatment.	Adopted^d^	N/A	Moderate
Chronic kidney disease	23. Children and adolescents with chronic kidney disease should be evaluated for hypertension at each medical encounter.	Adopted	Moderate	Strong
	24. Children or adolescents with both chronic kidney disease and hypertension should be treated to lower 24‐h mean arterial pressure < 50th percentile by ambulatory BP monitoring. In‐lieu of recent ambulatory BP data, a target office BP of the 50th percentile (acceptable range 25th to 75th percentile) measured by auscultation may substitute.	Adapted	Moderate	Strong
	25. Children and adolescents with chronic kidney disease and a history of hypertension should have BP regularly assessed by ambulatory BP monitoring, regardless of apparent control of BP with clinic measures. The frequency of measurement will depend on the history of agreement with other measures and degree of hypertension or target organ damage.	Adapted	Moderate	Strong
	26. Children and adolescents with chronic kidney disease and hypertension should be evaluated for proteinuria at each visit.	Adopted	Moderate	Strong
	27. Children and adolescents with chronic kidney disease, hypertension, and proteinuria should be treated with an ACEi or ARB.	Adopted	Moderate	Strong
Management	28. Children and adolescents with type 1 diabetes mellitus or type 2 diabetes mellitus should be evaluated for hypertension at each medical encounter and treated if BP ≥ 95th percentile or ≥ 140/90 mmHg.	Adapted	Low	Moderate
	29. In children and adolescents with acute severe hypertension and life‐threatening symptoms, initiate immediate treatment with short‐acting antihypertensive medication, and reduce BP by no more than 25% of the planned reduction over the first 8 h.	Adopted	Expert opinion	Weak
	30. Children and adolescents with hypertension should not be routinely excluded from sports participation. Children with significant cardiac changes should be discussed with a cardiologist.	Adapted	Very low^a^	Moderate
	31. Adolescents with high‐normal BP or hypertension (whether they are receiving antihypertensive treatment or not) should typically have their care transitioned to an appropriate adult care provider by 16–18 years of age (recognising that there may be individual cases in which this upper age limit is exceeded, particularly in the case of youth with special health care needs). There should be a transfer of information regarding hypertension aetiology and past manifestations and complications of the patient's hypertension.	Adapted	N/A	Strong

*Note:* Green is for 'adopted' and blue is for 'adapted' according to the methodology used.

^a^ Certainty of evidence grades that were downgraded based on new evidence or a different interpretation of the existing evidence.

^b^ There are benefits to exercise beyond BP alone. The World Health Organization and Australian Government recommend that young people (5 to 17 years) participate in 60 min of moderate to vigorous physical activity per day on average [33, 34].

^c^ Strength of recommendation upgraded.

^d^ Wording updated for clarity with no change in substance and/or to align with BP classification terms in Table 4.

### 3.1 Identification of Hypertension: Screening in Children and Adolescents

All international blood pressure guidelines that include children and adolescents recommend some form of BP screening in these age groups [23, 25, 26, 27, 35]. However, these guidelines conflict with the UK National Screening Committee who recommended against screening in either adults or children, and the United States Preventative Task Force (USPTF) who reported that there was insufficient evidence to assess the balance of benefits and harms [36, 37]. The latter has been debated widely and clinical groups believe that the level of evidence required by the USPTF was not realistic in the context of childhood hypertension [38, 39]. Randomised trials of BP screening or interventions in childhood linked to endpoints later in life are unlikely to be published in the foreseeable future. This does not negate the value of BP screening given the links between childhood BP and target organ damage, intermediate cardiovascular phenotypes, and cardiovascular and kidney events in adulthood [3, 8, 9, 10, 40, 41]. At a clinical level, BP in children should not be interpreted in isolation. Secondary hypertension is more common among children compared to adults, and any resultant investigations and interventions must account for the degree (stage) of hypertension, presence of target organ damage, and concurrent risk factors impacting the trajectory towards future clinical events (Tables 2, 3, 4).

Regarding the timing and frequency of screening, there was unanimous agreement that annual screening from 3 years of age as recommended in the AAP 2017 Guideline is presently not feasible in an Australian context within the limited existing framework for preventative health screening among school aged children. Currently, the healthcare professionals most visited by children are GPs, and those performing routine vaccinations. While both settings are not optimal in ascertaining an accurate BP in children due to the associated stress at the time of presentation, the former is more practical in obtaining a settled measurement and follow‐up of elevated results. Hence, recommendations need to be implementable in the context of current general practice workflows, time demands, and billing constraints in the Australian system. There are good arguments for community‐based screening. Further investment into the resources needed to document and act on abnormal values would be required to enact this in practice. Collaboration across the spectrum of cardiovascular and health promotion is required to continue developing opportunities for proactive identification and intervention for at‐risk children. It is also important that screening recommendations do not detract from broader public health measures to address cardiovascular risk factors across the life‐course.

The recommendation for an initial screening time point of 7 to 9 years of age was based on studies of BP tracking, and clinical experience that children of this age will typically co‐operate with the procedure for accurate BP measurement [42, 43, 44]. Importantly, visit‐to‐visit within‐individual BP variability is the same among children of this age and adults [45, 46]. The developmental stage of adolescence guided the second screening point of 13 to 15 years old, representing an important opportunity to identify and alter adverse health trajectories that without intervention strongly predict BP in mid‐adulthood [47]. The recommendation includes the term ‘approximately’ to indicate the need for some pragmatism when recommending infrequent screening. It may be appropriate to check BP at earlier or later ages in children for whom the opportunity may not otherwise arise.

Children at increased risk of hypertension, including those with obesity or taking stimulant medications, should have BP checked at every healthcare encounter from 3 years of age (Table 3). Other at‐risk children, such as those with kidney disease, a history of diabetes, a history of aortic arch obstruction or coarctation, are likely to be under specialist care which should align with relevant disease specific guidelines. Children with a history of pre‐term birth or a history of intra‐uterine growth restriction are at increased risk [13]. There are specific guidelines addressing BP monitoring as part of the holistic care for those graduating from the neonatal intensive care unit [48].

**TABLE 3:** Conditions associated with an increased risk of hypertension.

Overweight and obesity
Obstructive sleep apnoea
Diabetes mellitus (type 1 or 2)
Chronic kidney disease or congenital abnormalities of the kidney and urinary tract
Solid organ transplant recipients
Use of medications that increase blood pressure (e.g., stimulants, corticosteroids)
Repaired aortic arch abnormalities
Genetic syndromes associated with renovascular disease (e.g., neurofibromatosis type 1, Alagille syndrome, Williams syndrome)
History of prematurity or intra‐uterine growth restriction History of maternal hypertension during pregnancy
Parental history of hypertension with onset prior to 40 years of age

*Note:* This is not an exhaustive list. Other syndromes, medications may result in hypertension. It is important to exercise appropriate caution when managing children with rare diseases or receiving novel pharmacologic agents.

### 3.2 Defining Hypertension

BP in children is related to growth and development, increasing with age, height, and diverging by sex with the emergence of puberty [49]. A continuous relationship exists between BP and the risk of target organ damage and long‐term, downstream events such as myocardial infarction and kidney failure [3, 6, 9]. Data from the i3C consortium indicate that children with a BP in the high‐normal or hypertensive range at a mean age of 11.8 years (standard deviation 3.1 years) have a doubling of risk for fatal or non‐fatal cardiovascular events in adulthood (mean age 47.0 years, standard deviation 8.0 years) [3]. Children with a combined risk score (included BP, body mass index, youth smoking, triglycerides and total cholesterol) above the 95th percentile were more than 5 times as likely to experience a fatal or non‐fatal cardiovascular event before the age of 47 years.

Normalisation of BP for growth and development in childhood is typically achieved with sex‐stratified equations adjusted for age and height, or reference tables derived from these that give the relevant percentile thresholds. European and American guidelines were aligned, until participants with overweight and obesity were removed from the reference data for the AAP Guideline. This allowed for the inclusion of more recent data from populations with an increased prevalence of overweight and obesity, but from an epidemiology perspective the reference values are no longer based on a normal distribution which should include 15% of participants with overweight or obesity. One advantage of the new threshold values was that they were more contiguous with an updated US definition of hypertension among adults of 130/80 mmHg [23, 24, 50, 51]. Whilst subsequent studies have shown that the AAP Guideline thresholds have an increased sensitivity to detect target organ damage in children [52], this is expected because of the continuous relationship between risk and BP into what is typically considered the normal range [3]. Given this relationship, if thresholds are based on the lowest value associated with a statistically significant increase in risk, then the main determinant of thresholds will be the sample size of the included population. Hence, in lieu of evidence for clinically meaningful improvements in overall diagnostic test accuracy at lower thresholds, the panel recommends using the ESH reference for BP percentiles (equivalent to previous United States Fourth Report Guideline). As importantly, these are more consistent with the current definition of hypertension in Australian adults of 140/90 mmHg at 18 years of age [49, 51, 53, 54]. Further research is in progress, including data from the SHIP‐AHOY study and i3C that may lead to this recommendation being updated. The principle of maintaining consistency with guidelines for Australian adults would also require the paediatric definition to be revisited should the threshold for defining hypertension in the adult population change in the future.

The relevant tables of BP percentiles were published with the 2016 ESH guidelines for the management of high blood pressure in children and adolescents and reaffirmed in the combined 2023 ESH guideline [24, 25]. An online calculator relevant to the Australian guideline is available online (https://www.hypertension.org.au/paed‐calculator). The National Heart Foundation definition of hypertension should be used from 18 years of age or any child for whom the 95th percentile is ≥ 140/90 mmHg [20]. The National Heart Foundation Guideline includes thresholds for high‐normal BP and classification of the severity of hypertension that are analogous to those defined by the AAP Guideline for children (Table 4). The panel endorsed the classification of BP according to this approach.

**TABLE 4:** Thresholds for the diagnosis of hypertension.

Clinic BP	Paediatric values	Adult values^a^
Normal BP	≤ 89th percentile	≤ 129 mmHg and ≤ 84 mmHg
High‐normal BP	90th to 94th percentile	130–139 mmHg and/or 85–89 mmHg
Stage 1 hypertension	≥ 95th percentile	140–159 and/or 90–99 mmHg
Stage 2 hypertension	≥ 95th percentile +12 mmHg	160–179 and/or 100–109 mmHg
Severe hypertension^b^	≥ 95th percentile +30 mmHg	≥ 180 and/or ≥ 110 mmHg

*Note:* When a patient's systolic and diastolic stages are discordant, the higher diagnostic category applies.

^a^ This is the threshold for people ≥ 18 years of age or those in whom the percentile‐based value is greater. These values are taken from the National Heart Foundation of Australia 2016 Guideline [20].

^b^ Symptoms are an important part of assessing children with severe hypertension. Acute severe hypertension with symptoms may occur at lower values with a rapid increase in blood pressure (see Section 3.15).

For the purposes of screening in primary care, it was decided to adopt simplified, age‐stratified thresholds from the Hypertension Canada 2020 Guideline. These are 120/80 mmHg for children aged 6 to 11 years and 130/85 mmHg for children aged 12 to 17 years. The panel agreed that the potential advantages in implementation outweigh the potential disadvantages in reduced sensitivity for the detection of hypertension in children whose BP exceeds the 95th percentile by a small amount at the lower end of the relevant age ranges [26]. Once a child has been identified to exceed the simplified screening thresholds, then the BP percentile should be calculated to help quantify the extent of BP elevation, guiding further investigation and management. This recommendation may be updated as more evidence about the relationship between BP in childhood and cardiovascular events in adult life becomes available. The recommendation to include BP alerts for children in digital medical record systems was adopted, noting the need for careful implementation and benefit of working with providers (e.g., development of standardised templates) to avoid alert overload.

It is important to confirm that BP measurements exceeding the threshold for hypertension are persistent prior to initiating further investigations or treatment (Figure 1). For children with stage 1 hypertension, the AAP Guideline recommends doing so on three separate occasions over a three‐month period. Children with stage 2 hypertension should have a second visit in a short time prior to referral; the AAP Guideline recommends 1 week, although this may not always be practical and depends on the degree of elevation. Children with acute severe hypertension require urgent review by an appropriate specialist.

**FIGURE 1:**
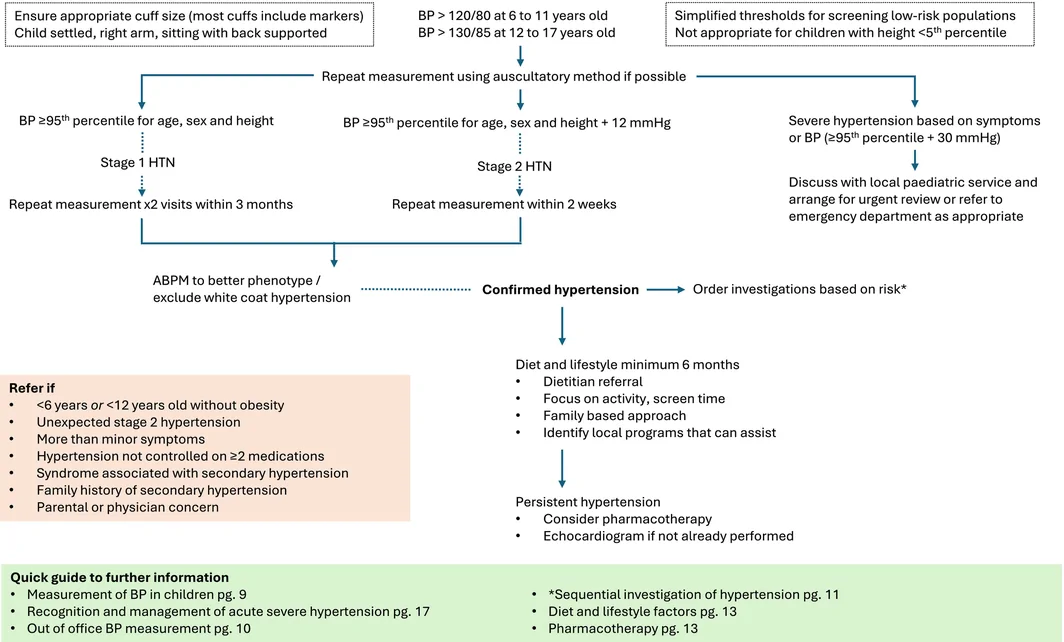
Clinical pathway for the identification and management of hypertension in children and adolescents.

### 3.3 Measurement Methods

It remains true that optimal methods of BP measurement are not always consistently applied in routine clinical practice [55]. Variations in technique such as using an undersized cuff or not waiting until a child is adequately settled may produce artificially high readings [56]. Regardless, repeated values exceeding the 95th percentile are frequently not recognised, with or without an electronic medical record [57, 58]. Limitations in implementing traditional age, sex, and height adjusted paediatric definitions were one motivating factor for the adoption of simplified screening thresholds for screening children 6 years and older. However, these do not eliminate the issue of needing to standardise methods of BP measurement for all children [59].

The panel endorsed the AAP 2017 recommendations regarding BP measurement methodology. Cuff selection and placement is important to obtain an accurate BP (Table 5) [60]. The bladder length should be 80%–100% of the circumference of the arm, and the width should be at least 40%. The former is similar to the ISH 2020 Global Hypertension Practice Guidelines (bladder length 75%–100% of arm circumference), the latter has been demonstrated to be more accurate in children and remains commonly used [56]. A cuff width to arm circumference ratio of 0.45 to 0.55 is also recommended in infants as it produces more accurate readings [61]. BP should be measured on the right upper arm. This reflects the methods used to derive the reference values defining hypertension and allows for the detection of hypertension due to coarctation of the aorta. Arm positioning is important, with randomised crossover trial data confirming that arm positioning by the side or lap increases BP potentially resulting in over‐diagnosis of hypertension [62]. If a leg BP is indicated (e.g., vascular access or injuries to upper limbs) the expected systolic BP value is 10%–20% higher than for the upper limbs [63, 64]. Regarding caffeine intake, current guidelines for BP measurement refer to abstaining from caffeine immediately before up or ≤ 30 min prior to measurement [55, 65]. This is a balance between the degree of blood pressure elevation after consumption of a typical caffeinated beverage (2–5 mmHg) and practicality of obtaining office BP when patients have not been advised to fast, against peak plasma levels being achieved at ~60 min post ingestion and a half‐life of caffeine of 4 to 6 h (less among young children) [66, 67, 68, 69]. The panel recommended maintaining consistency with standard recommendations, but for confirmatory readings to ensure abstinence from caffeine for 4 to 6 h prior to measurement.

**TABLE 5:** Measurement of blood pressure in children.

An automated oscillometric device is appropriate for screening but it is important to ensure the device has been validated for accuracy in children^a^.
In an otherwise well child, prior to making a formal diagnosis or initiating treatment^b^:
− The blood pressure should be measured on three separate occasions.
− The blood pressure should be confirmed by the auscultatory method using a well‐maintained manual device.
The right upper arm is the preferred measurement location, followed by the left upper arm. Leg blood pressure should only be used if there are medical contraindications (e.g., injuries or devices) to upper arm measurement. The arm should be supported on a desk or table, with the cuff positioned at heart level. Wrist and cuffless blood pressure devices are not currently recommended.
The bladder length should be 80%–100% of the arm circumference and the bladder width at least 40%.
In infants, the arm circumference to bladder width ratio should be 0.45 to 0.55.
The child should be in the seated position, with the back supported and feet resting flat on the floor^c^.
Ideally, infants should not be sucking/feeding at the time of measurement.
Avoid exercise or caffeine 30 min prior to measurement^d^.
The child should be settled, having rested for at least 3 to 5 min.
Take at least 2 measurements ≥ 30 s apart, ideally 3, discarding the first measurement and averaging the 2nd and 3rd values obtained.

*Note:* Advice regarding cuff size and positioning, and patient preparation, applies to both oscillometric and auscultatory methods.

^a^ See STRIDE BP for a current list—https://www.stridebp.org.

^b^ See Table 2 for detail on diagnosis and treatment.

^c^ If an appropriate chair is not available, then lying BP is preferred.

^d^ For confirmatory measurements, it is recommended to avoid caffeine for 4 to 6 h prior to measurement.

Oscillometric devices produce a similar average BP value to auscultatory methods at the population level (average difference systolic BP + 2.5 mmHg) [70]. They measure the mean arterial pressure by proxy of the amplitude of oscillations in pulse pressure, and then use proprietary equations to estimate systolic and diastolic values. Oscillometric devices are increasingly the predominant methods for BP measurement in adults and calibrated oscillometric sphygmomanometers are more readily available in most settings, making them an appropriate choice for BP screening. However, there are some important caveats and manual methods are recommended for confirming hypertension in children and adolescents prior to detailed investigations or treatment. It is important that oscillometric devices are validated according to a recognised international standard such as ISO81060:2. Relatively few devices have met the validation criteria in children (considered a ‘special population’ for BP device validation) and high heterogeneity is observed among validation studies [71]. The best contemporary source of validated devices is maintained by STRIDE BP (https://www.stridebp.org). In a clinical setting, significant differences may be observed between oscillometric and auscultatory methods. In addition, manual (i.e., auscultatory) BP performed by an experienced operator is often better tolerated by young children. As such, the panel agrees with the AAP, ESH and Hypertension Canada guidelines in recommending confirmation of elevated readings by manual measurement for children and adolescents. Cuffless devices and wrist‐based devices are not recommended at this time given limited data on accuracy.

### 3.4 Out of Office BP Measurement

Ambulatory blood pressure monitoring (ABPM) involves measuring BP at regular intervals over ≥ 24 h using approved devices and standardised methods to obtain information about out of office BP, providing further data on BP variation including during sleep. It is the reference standard for evaluating BP because it better predicts clinical outcomes, including target organ damage among children and adolescents [72, 73, 74, 75]. Including ABPM as a routine diagnostic procedure also facilitates the identification of children with white coat hypertension, who do not need extensive investigation or treatment [76]. Normative ABPM data for children have been established by the German Working Group on Pediatric Hypertension in 2002 and the resultant tables of BP percentiles continue to be used by both the AAP and ESH guidelines [25, 77, 78]. These data include children from ≥ 5 years of age and/or ≥ 120 cm tall. The percentile tables provided are sex‐stratified and adjusted for age or height; however, the height adjusted tables are preferred [78]. The use of a percentile‐based definition for adolescents has been shown to better predict LVH than fixed thresholds, although what defines an optimal balance of sensitivity and specificity remains open to debate and fixed thresholds have also been proposed [52, 79]. The panel endorsed using the 95th percentile by sex and height provided these are less than the relevant adult thresholds to define hypertension by ABPM (Table 6). It is useful to note that, unlike adults, mean daytime ABPM values for children are higher than clinic or home blood pressure (HBP) values, which is reflected in the daytime ABPM percentiles [77, 80, 81]. ABPM for children and adolescents is currently accessed via paediatric nephrology units operating in tertiary hospitals. However, upcoming changes to the Medicare Benefits Schedule to include age‐appropriate percentile criteria for children should lead to improved community access.

**TABLE 6:** Out‐of‐office thresholds defining hypertension.

Measure	Value
ABPM—day average	95th percentile^a^ or ≥ 135 mmHg and/or ≥ 85 mmHg
ABPM—night average	95th percentile^a^ or ≥ 120 mmHg and/or ≥ 70 mmHg
ABPM—24‐h average	95th percentile^a^ or ≥ 130 mmHg and/or ≥ 80 mmHg
Home blood pressure	≥ 135 mmHg and/or ≥ 85 mmHg

*Note:* Static thresholds are taken from the National Heart Foundation of Australia 2016 Guideline [20].

Abbreviation: ABPM, ambulatory blood pressure monitoring.

^a^ Use the 95th percentile (Wuhl et al. [77]) or static cut‐off, whichever is lower.

An American Heart Association update to the AAP 2017 Guideline for ABPM in Children and Adolescents was published in 2022 [78]. This document provides a comprehensive description of how and why to perform ABPM in children. There was one important change to the diagnostic criteria for hypertension by ABPM in that guideline, with the removal of load criteria based on new data that failed to demonstrate benefit by the inclusion of BP load to predict LVH in children [52, 82]. As a result, the panel removed AAP KAS 9. AAP KAS 10 was adapted (Item 9, Table 2) to allow for the use of HBP to diagnose white coat hypertension, although ABPM will mostly be preferred for this purpose unless a family already has experience with HBP and appropriate equipment available.

HBP monitoring is further discussed as it relates to monitoring ongoing treatment later. HBP is commonly used in adults, supported by high quality evidence linking HBP to clinical outcomes including mortality [20]. Relatively few studies have been performed in children regarding the use of HBP for the diagnosis of hypertension compared to ABPM [72]. These have generally reported poor sensitivity (52% to 55%) of HBP for the diagnosis of hypertension and masked hypertension, but better sensitivity and specificity (89% and 92%, respectively) for the detection of white coat hypertension when compared to ABPM [83, 84]. Studies in children have also shown higher concordance between daytime ABPM measurements and HBP compared to clinic BP, and that HBP better predicts target organ damage than clinic BP [73, 75]. Additionally, in clinical practice HBP monitoring is becoming increasingly readily available, with more households having access to home BP measurement devices, making it a practical way of assessing adequacy of treatment compared to repeated ABPM. Recommendations for performing HBP in children mirror those for adult populations [65]. HBP should be performed for 7 days (minimum 5) in a manner similar to that for optimal clinical BP; resting, feet flat on the floor, with an appropriately sized cuff and validated machine [85]. Further resources including a HBP patient diary and advice for accurate measurement are available via the Hypertension Australia website (https://www.hypertension.org.au/children/).

### 3.5 General Investigation of Hypertension

The AAP 2017 and ESH 2016 Guidelines represented an important change in recommendations for investigating childhood hypertension. Previously, essential hypertension was considered uncommon and all children with an increased BP underwent a broad range of investigations for possible secondary causes. However, this is no longer the case and more directed investigations are now recommended for those with Stage I hypertension, guided by a thorough history and examination [86]. This change was brought about by population‐based research demonstrating that most hypertension in youth is essential, and in many places the prevalence of hypertension is increasing along with the prevalence of obesity [87]. Young children, those with severe hypertension, children of a normal weight, children with a family history of secondary hypertension, or patients from selected populations (e.g., with diseases or syndromes predisposing to hypertension) may all have a higher likelihood of secondary causes and thus require additional investigations [88, 89]. Children with a normal nocturnal BP and dipping pattern are unlikely to have a secondary hypertension [90, 91, 92, 93, 94]. In addition to investigating for possible causes of secondary hypertension, it is important to consider screening for concurrent cardiometabolic risk factors and target organ damage. Routine ophthalmology review is not recommended as part of the initial evaluation of children with stage I or early‐stage II hypertension, hypertensive retinopathy being rare in children without severe hypertension and/or an identified secondary cause [95].

Table 7 summarises the recommended sequential investigation of childhood hypertension. This pathway aligns with the AAP 2017 Guideline, aside from the recommendation for routine kidney ultrasound. Accurate data on the diagnostic yield of screening ultrasound are hard to obtain, with studies including children referred to specialist hypertension clinics indicating that contributory ultrasound findings are common among children without other abnormalities, and that approximately half of children with chronic kidney disease (CKD) do not have proteinuria [96, 97]. Yet, among 321 children enrolled in trials of valsartan, renal tract ultrasound abnormalities were uncommon among those ≥ 6 years of age [88]. Detailed discussion about the indications for and rationale for specific investigations can be found in other sources, along with the following sections reviewing cardiac and renovascular investigations [29, 86]. The AAP 2017 and ESH 2016/2023 Guidelines recommended the removal of routine renin and aldosterone testing for children with hypertension at low risk of secondary causes. These tests have a low yield even among children presenting to tertiary hypertension clinics and poorly predict undetected renovascular disease [96, 98, 99]. Primary aldosteronism is an area of active research; however, data indicate a continuum of aldosterone dysregulation and diagnostic thresholds vary widely [100, 101]. Measurement of systemic renin and aldosterone remain part of screening for young children or those with severe hypertension. Low renin values should prompt investigation for monogenic disorders of hypertension, all of which are associated with low renin aside from autosomal dominant hypertension with brachydactyly [29].

**TABLE 7:** Sequential evaluation of childhood hypertension.

All children	Urea and electrolytes, serum calcium/magnesium/phosphate, serum creatinine
	Proteinuria—uACR, uPCR. First morning measurement necessary if random spot value is elevated^a^
	Renal tract ultrasound with Doppler^b^
	Echocardiography^b^
Risk stratification and detection of metabolic syndrome	Non‐fasting lipids
	HbA1c (plus fasting glucose in high‐risk)
	Liver function tests (liver ultrasound in high‐risk)
Consider based on history and examination, or any child with severe or difficult to control hypertension^e^	Direct renin concentration and serum aldosterone
	Thyroid function tests
	Urine or plasma metanephrines
	Cortisol testing—any of: low‐dose dexamethasone suppression test, 24‐h free urinary cortisol, and late‐night salivary cortisol^c^
	Dimercaptosuccinic acid scan
	Cross sectional imaging or digital subtraction angiography^d^
	Sleep study
	Full blood picture
	Testing for monogenic disorders: genetics, urine and plasma steroid profile
	Inflammatory markers
	Drug screen

*Note:* Adapted from Larkins N, Roebuck D (2022). Diagnostic Evaluation of Pediatric Hypertension. In: Flynn JT, Ingelfinger JR, Brady T (eds) Pediatric Hypertension. Springer, Cham [29].

^a^ Orthostatic proteinuria is common in children and adolescents (~20% prevalence) [30, 31, 32]. uACR, urine albumin‐to‐creatinine ratio; uPCR, urine protein‐to‐creatinine ratio. A negative or trace result by dipstick may substitute providing urine specific gravity is ≥ 1.005.

^b^ If not routinely, then in young children (< 6 years) and those being considered for pharmacotherapy.

^c^ Urinary or salivary cortisol should be measured twice to minimise the impact of day‐to‐day variability.

^d^ The latter being preferable where there is a high index of suspicion.

^e^ Severe or difficult to control hypertension—any of: unexpected grade II hypertension, more than minor symptoms, hypertension not controlled on two or more medications.

### 3.6 Cardiac Investigations

The detection of LVH remains an important component of care, helping to identify children with substantial target organ damage that require a more extensive diagnostic workup, and as a serial measure in assessing the efficacy of treatment. For children with stage I hypertension, demonstrating target organ damage can be a strong motivating factor to initiate lifestyle modifications, or conversely may support the role of pharmacologic treatment in children whose BP has not responded to non‐pharmacologic approaches. The relevant AAP statements were adopted (including that ECG is not a useful test to screen for LVH), except for 15–2 regarding the definition of LVH [102]. The definition of LVH used by the AAP 2017 Guideline is based on recommendations from the American Society of Echocardiography and the European Association of Cardiovascular Imaging that link left ventricular parameters to clinical outcomes among adults [23, 103]. However, there remains controversy about the optimal measure to define LVH and no singular metric on echocardiography has been found to outperform clinical interpretation utilising all available information. This is not to say that systematic reporting of left ventricular markers such as body surface area adjusted left ventricular mass (LVMI) is not useful, but they should be interpreted in context and the overall clinical interpretation incorporating all available measures prioritised.

### 3.7 When and How to Investigate for a Renovascular Cause

Renovascular disease is among the most common secondary causes of hypertension in children and remains important to detect because directed interventions may be available (e.g., angioplasty). The likelihood of renovascular disease increases with increasing BP and reducing age, or the presence of syndromes known to be associated with vascular disease in children (Table 8). Kidney ultrasound with Doppler imaging is the appropriate initial test when renovascular disease is suspected. It carries less cost and burden of care than other methods. Some findings, such as tardus parvus waveform are considered relatively specific. However, the overall sensitivity for ultrasound remains low (73%) and it is relatively operator dependent [104]. Cross sectional imaging modalities, such as computed tomography (CT) and magnetic resonance imaging (MRI), have a role in the investigation of renovascular disease. However, differences in aetiology, the distribution of disease and vessel size mean cross sectional imaging remains less sensitive as a diagnostic test in children compared to adults [104, 105]. Hence, direct angiography is still required when there is a high index of suspicion for renovascular disease [29, 86]. There are specific situations in which CT or MRI can be helpful (e.g., monitoring disease progression, in larger patients with a low pre‐test probability, MRI to assess vessel wall involvement in vasculitis) and in centres without access to direct angiography they are more likely to form part of the diagnostic work‐up [105, 106].

**TABLE 8:** Potential indications for further imaging after a normal renal ultrasound.

Young age
Unexplained stage II hypertension
Hypertension not controlled on two or more medications
Presentation with acute severe hypertension or sequalae (e.g., heart failure, facial nerve palsy or posterior reversible encephalopathy syndrome)
Biochemical evidence of hyperaldosteronism (e.g., hypokalaemia)
Known or suspected vasculitis (e.g., Takayasu arteritis or polyarteritis nodosa)
Syndromes associated with renovascular disease
− Neurofibromatosis type 1
− Alagille syndrome
− Williams syndrome
− Other
Abdominal bruit
Risk factors for an iatrogenic vascular anomaly on history
− Umbilical artery catheterisation or renal artery thrombosis
− Retroperitoneal surgery (e.g., neuroblastoma resection)
− Abdominal radiotherapy
End‐organ damage
− Left ventricular hypertrophy
− Hypertensive retinopathy

*Note:* Adapted from Larkins N, Roebuck D (2022). Diagnostic Evaluation of Pediatric Hypertension. In: Flynn JT, Ingelfinger JR, Brady T (eds) Pediatric Hypertension. Springer, Cham [29].

### 3.8 Albuminuria as a Routine Test

The KAS advising against the use of albuminuria as a screening test in children was not adopted, to maintain consistency with other guidelines and avoid confusion for general practitioners implementing them. Notable organisations such as Kidney Health Australia actively promote albuminuria as the test of choice for kidney health checks and there was concern that such a recommendation advising against the use of albuminuria as a screening test could create confusion, conflict with international (Kidney Disease: Improving Global Outcomes) guideline recommendations for initial testing of albuminuria and proteinuria in children, and undermine the role of albuminuria testing in adults.

Testing for low‐level albuminuria (sometimes termed microalbuminuria) is typically not recommended for children outside of specific situations, such as diabetes mellitus when there are clear reference ranges and treatment implications [107]. Albuminuria is a fraction of total proteinuria, recommended in screening adult populations for kidney disease. However, given the analytic accuracy at low levels is less relevant, and differences in the aetiology of kidney disease among children and adolescents, total protein is the preferred measure for people < 18 years of age [23]. Urine dipstick is sufficiently sensitive to rule out significant proteinuria in most settings, provided the sample is adequately concentrated (specific gravity ≥ 1.005). A result of ≥ 1+ should prompt urine protein‐to‐creatinine measurement, < 20 mg/mmol being a normal result. The timing of spot urine samples is important and elevated random measures need confirmation on a first‐morning test. This is because orthostatic variation is exaggerated in children and adolescents, particularly among children without obesity, and the most common cause of erect proteinuria in this age group is benign orthostatic proteinuria [30, 31, 32, 108].

### 3.9 Treatment Targets in the General Paediatric Population

The panel endorsed the recommendation to target a BP < 90th percentile when treating children and adolescents with hypertension. While there is limited evidence to support specific treatment targets in young people, this level of treatment control is usually achievable in clinical practice and aligns with the 130/80 mmHg Roadmap of the National Hypertension Taskforce of Australia for adults [19]. Out of office measurements are preferred (see Section 3.4). However, for clinical values a time‐average approach is also recommended, such that if the majority of measurements are below the target value, then control is considered sufficient. Among adults at high risk of cardiovascular events and children with CKD, lower treatment targets have demonstrated net benefit in randomised controlled trials (RCTs) (further discussed below in relation to Item 24, Table 2). The balance between cumulative risk associated with hypertension and exposure to potential adverse effects from interventions remains to be tested by RCT in children. Acknowledging the inherent difficulty in performing such a trial, we do not expect further direct evidence to be available in the near future.

### 3.10 Non‐Pharmacologic Interventions

Emerging evidence for the non‐pharmacological treatment of hypertension in children and adolescents is comparable to that observed and recommended in adults and is focussed on nutrition and physical activity with follow‐up every 3–6 months (see evidence review in Table A4). The panel therefore endorsed that at the time of diagnosis of elevated BP, clinicians should provide advice on the DASH (Dietary Approaches to Stop Hypertension) diet and recommend moderate to vigorous physical activity at least 3–5 days per week (30–60 min per session) to help lower BP. The benefits of physical activity for physical and mental health extend beyond BP alone, and children should be encouraged to gradually increase physical activity to meet the current World Health Organization guidelines, which recommend an average of at least 60 min moderate to vigorous physical activity per day [33]. It is estimated that 25% of younger children and ≤ 10% of Australian adolescents are meeting this at a population‐level [109]. Care within multi‐disciplinary teams and/or the engagement of allied health professionals in the community is encouraged given the importance of these components of care to long‐term outcomes.

The DASH diet results in lower BP in children and adolescents, particularly with overweight and obesity, metabolic syndrome and type 2 diabetes mellitus [110, 111, 112, 113]. With considered modifications, the DASH diet can be effectively translated across different ethnicities and among culturally and linguistically diverse groups, although the effect size may vary [114, 115, 116]. The DASH diet is typically instituted in combination with a low‐sodium diet per the seminal trial demonstrating independent and combined benefits to these interventions for adults [117]. Trials among children specifically targeting salt intake demonstrate similar benefit, and observational data are also consistent with the impact being greatest among those starting with a higher BP [118, 119]. In some settings, such as children with salt losing tubulopathies or living remote conditions in hot environments, standard salt recommendations may need to be adjusted upwards due to increased salt losses. While there are data from diverse populations indicating that the principles of the DASH diet are broadly generalisable, it is important to recognise that the emphasis remains on delivering a healthy, culturally appropriate nutrient intake. Engaging a dietitian as part of the healthcare team is recommended to allow for more detailed assessment of diet than is usually possible in a standard medical appointment and to provide personalised, culturally appropriate advice.

Higher levels of physical activity are associated with lower BP independent of diet or dietary interventions, and total physical activity volume may be more important than intensity, although both contribute [120, 121]. Interventions from 30 min of aerobic exercise, 3 times per week have demonstrated benefit [122]. Observational data support a reduction in systolic BP with up to 150 min of at least moderate physical activity per week, noting that there are other benefits to more frequent physical activity and the current recommendation for children (5 to 17 years old) generally is to aim for 60 min of moderate to vigorous physical activity per day [34, 123]. Item 20 (Table 2) relating to physical activity was adopted, which sets lower minimum targets initially (30–60 min, 3–5 times per week), acknowledging that for many children meeting the 60‐min broader recommendations represent a large increase in physical activity and usually best achieved and maintained by incremental improvements. Additionally, it is recommended that children from birth to 2 years not spend time in front of screens, from 2 to 5 years limit screen time to 1 h per day, and from 5 to 17 years limit screen/sedentary time in general [33, 34].

Family‐centred interventions to improve BP and cardiovascular risk are typically more effective than those that target children alone [124, 125]. Family‐centred counselling may involve a dietitian and/or exercise physiologist, and techniques to promote positive and sustainable behaviour change (e.g., motivational interviewing) [126]. Parental smoking is an independent risk factor for hypertension, and eliminating environmental exposure to smoking and vaping is recommended [127]. While data are limited, it is suggested to optimise sleep hygiene [128]. Other approaches such as breathing‐awareness meditation and mindfulness are also being explored as potential components of treatment [129]. Non‐pharmacologic therapy is summarised in Table 9, but we would note that strength of evidence varies by modality and the evidence‐base continues to expand. Non‐pharmacologic interventions may demonstrate effect modification dependent on the societal, geographic, social, and cultural context of care.

**TABLE 9:** Non‐pharmacologic therapy.

Diet^a^
Dietary approaches to stop hypertension (DASH)
Low‐salt diet
Limit intake of highly caffeinated beverages (‘energy drinks’)
Activity
Moderate to vigorous physical activity at least 3–5 days per week (30–60 min per session)
World Health Organization recommends an average of at least 60 min moderate to vigorous exercise per day for overall health and wellbeing
Limit screen time—none below 2 years old, and 1 h per day from 2 to 5 years of age.
Environmental factors
Parental or child smoking
Sleep hygiene

*Note:* Care within multidisciplinary teams employing a whole of family approach accounting for socioeconomic and social context, health literacy, and culture is more likely to promote clinically significant and durable lifestyle modifications.

^a^ More detailed dietary advice for families and healthcare providers is provided at https://www.hypertension.org.au/children. Children with congenital and tubular abnormalities require a specific salt prescription based on their condition and progression of glomerular filtration rate.

Early referral to services providing support for children above a healthy weight should be considered for all children above an obesity cut‐point with essential hypertension. Effective support to address obesity requires a multidisciplinary and team‐based approach, incorporating medical, dietetic, and psychological expertise to address the complex physiological and societal drivers of obesity [130]. While specific clinical pathways vary by state, service delivery typically follows a tiered model: community‐based healthy lifestyle programs (e.g., Go4Fun) provide family‐centred support for children and young people without significant weight‐related comorbidities, whereas tertiary hospital obesity clinics offer intensive, specialised interventions for children affected by severe obesity or comorbidities requiring ongoing management. Both hospital and community‐based services will continue to evolve to further incorporate evidence of the impact of specific programs on BMI and BP, along with the expanding pharmacologic options such as glucagon‐like peptide‐1 receptor agonists [131, 132, 133]. Hypertension is often incorporated into referral criteria, along with other cardiometabolic risk factors that should be routinely screened for: dyslipidaemia, dysglycaemia and diabetes, and metabolic dysfunction‐associated steatotic liver disease. Beyond the aforementioned impact on BP measurement, the provision of an appropriate range of blood pressure cuffs is an important component of providing a clinical environment that minimises weight stigmatisation [130].

### 3.11 Pharmacologic Management

The criteria for initiation of pharmacotherapy are summarised in Table 10, the Practice Points box highlights considerations for selecting an anti‐hypertensive agent, and commonly used agents and dosing are outlined in Table A10. The AAP 2017 Guideline does not recommend a different treatment initiation threshold for children with diabetes mellitus or CKD, which is consistent with the current National Heart Foundation of Australia Guideline [20, 23]. However, as with the initiation of any long‐term pharmacotherapy, a personalised, shared decision making approach is endorsed. Specific cardiovascular risk calculators, such as those used for adults > 30 years of age in the 2023 Australian guideline for assessing and managing cardiovascular disease risk, are not designed for use in young people [21]. Factors influencing the decision to start pharmacotherapy will include the presence of other risk factors for downstream events (mainly cardiovascular and kidney disease), family history, response to non‐pharmacologic treatment and BP trajectory over time. As for diagnostic procedures, when considering interventions, it is important to recognise the continuous relationship between BP and risk. The thresholds introduced in this guidelines and others aim to simplify care to facilitate clinical management and inform good decision making, rather than introduce significant differences in management for patients that fall one side or another of a threshold; for example, an adult sized patient with a systolic BP of 138 is not markedly different from one with a BP of 141 mmHg, and the decision to treat or not will depend on all of the factors discussed, and the patient and family approach to risk from treatment compared to observation after being given all the relevant information.

**TABLE 10:** Criteria for pharmacologic antihypertensive therapy.

Symptomatic hypertension
Target organ damage
Stage 2 hypertension without an obvious cause (e.g., obesity)
Hypertension (Stage 1 or 2) without target organ damage that persists ≥ 6 months despite a trial of non‐pharmacologic therapy^a^

^a^ See text for more detailed description about personalised decision making for patients in this category.

Limited short‐ and medium‐term trial data exist to guide the specific choice of pharmacologic agents in children [134]. Hence, the recommendation from the AAP and adult populations favouring angiotensin converting enzyme inhibitors (ACEi) / angiotensin receptor blockers (ARB), and long‐acting calcium channel blockers was adopted, with the exception of thiazide diuretics, which are typically reserved as a 2nd or 3rd line agent to counteract salt retention in a multi‐modal anti‐hypertensive regimen. In practice, some healthcare providers favour long‐acting calcium channel blockers as the 1st line agent in children because these do not require serial monitoring of electrolytes and have a favourable safety profile. However, there are situations when ACEi/ARB may be preferred due to a better renoprotective profile, for example in children with kidney disease or disease processes in which glomerular hyperfiltration is a key component of disease progression (e.g., diabetes mellitus). The teratogenicity of ACEi/ARB on foetal kidney development is well described. Advice about the use of contraception, and using an alternate anti‐hypertensive agent if planning a pregnancy or when a pregnancy is confirmed, are essential components of care. Renin‐angiotensin system blockade via ACEi/ARB may have specific benefits with regards to cardiac remodelling in some settings, although patients with a significant cardiac history (anything other than mild or moderate LVH) should be managed in conjunction with a cardiologist [135, 136]. When (glomerular) proteinuria is present, then a ACEi/ARB will typically be indicated given the likelihood of additional independent anti‐proteinuric benefit regarding the risk of future cardiovascular and kidney disease [137, 138, 139]. Novel therapies have emerged for the treatment of resistant hypertension [140]. As with any new pharmacotherapeutic agent caution is required with initial use in children to ensure there are no age‐dependent side effects, effect modification, and that differences in pharmacokinetics are considered. As such, these are initially in the setting of clinical trials or severe, multi‐agent resistant hypertension.

Evidence from adults supports the use of low‐dose combination medications over monotherapy initiation [141, 142]. BP control is better, and the frequency of adverse events is similar overall, probably because of a dose‐dependent relationship with side effects for calcium channel blockers and thiazide diuretics [141]. The Roadmap of the Hypertension Taskforce of Australia endorses the use of single‐pill combinations and the Pharmaceutical Benefits Scheme (PBS) has recently been updated to allow the use of dual‐agent combinations as first‐line pharmacologic therapy [19]. However, in children, evidence for this approach is lacking, aside from a 12‐week randomised, blinded, placebo‐controlled trial of bisoprolol‐hydrochlorothiazide [143]. Therefore, the panel endorsed the AAP 2017 recommendation to start with a low‐dose single agent when pharmacotherapy is required. When treating older children with essential hypertension, consideration should be given to adding a second agent rather than single‐agent dose escalation when required.

### 3.12 The Role of ABPM in Assessing Treatment Efficacy

The panel endorsed the KAS that ABPM may be performed to monitor treatment efficacy. ABPM is the best measure of treatment control. However, it is a more resource‐intensive test than office BP, carries a burden of care in terms of discomfort and inconvenience, and is not uniformly accessible across all healthcare settings. There are also regulatory barriers with funding only available for diagnostic purposes, and limited to one test per year per patient. There also remains some uncertainty about the quantum of incremental clinical benefit derived from repeated ABPM as a part of routine care in any age group. An ABPM sub‐study of the Systolic Blood Pressure Intervention Trial (SPRINT) trial indicated no difference in the prevalence of masked uncontrolled hypertension between groups (20%–30% depending on the threshold used), despite the greater prevalence of sustained controlled hypertension in the intensive treatment arm [144]. This is concerning because masked uncontrolled hypertension is associated with adverse clinical outcomes in the same way as masked hypertension prior to treatment [145, 146]. However, the additional utility of escalating drug therapy to treat masked hypertension is less certain and along with limited reproducibility data for ABPM in children, it is difficult to make definite recommendations regarding the frequency of ABPM when monitoring treatment efficacy. The risks associated with masked uncontrolled hypertension by HBP are similar to those for ABPM [146, 147]. HBP and increasingly automated unattended office BP measurements are becoming an important adjunct in real‐world care and reduce some of the gap between reference standard, ABPM and clinic BP measurements [148, 149]. Regardless, ABPM remains the reference standard when there are differences between measurement methods and should be repeated after 3 to 6 months to confirm treatment adequacy when there is uncertainty about the reliability of other methods in a given patient.

### 3.13 Treatment of Children With Hypertension and Kidney Disease

Cardiovascular disease is among the most common causes of mortality in children with CKD, accounting for ~1/3 of deaths among those with kidney failure [150]. Blood pressure is intrinsically linked to kidney function via the role of the kidney in maintaining salt and water homeostasis. However, there is a bidirectional relationship between BP and kidney function, such that hypertension may be an important early indicator of kidney disease and also hastens the progression of CKD. The Effect of Strict Blood Pressure Control and Angiotensin Converting Enzyme Inhibition on the Progression of Chronic Renal Failure in Pediatric Patients (ESCAPE) trial demonstrated improved kidney outcomes with intensive blood pressure targets, comparing an ABPM target < 50th percentile to targeting between the 50th and 95th percentiles, a finding that translated to adult populations with CKD stage ≥ 4 [151, 152]. Recently, the Hypertension Optimal Treatment in Children with Chronic Kidney Disease (HOT‐KID) trial was conducted to determine if a similar treatment effect could be observed for left ventricular remodelling among children with CKD and LVH, but using auscultatory, office BP to monitor treatment efficacy [153]. The primary outcome (ΔLVMI) was not met, although the population enrolled had a lower mean LVMI than anticipated and a significant reduction in relative wall thickness (secondary outcome) was observed, in addition to directions of effect generally favouring intensive treatment. The lack of difference in primary outcome left in HOT‐KID leaves uncertainty about the net benefit of treating to SBP by auscultatory, office BP. The panel recommended that the treatment target should be to lower 24‐h mean arterial pressure < 50th percentile by ambulatory BP monitoring. In‐lieu of recent ambulatory BP data, a target office BP of the 50th percentile (acceptable range 25th to 75th percentile) measured by auscultation may substitute.

Trials of intensive BP lowering among adults have used a variety of BP measurement methods, including automated office BP in the SPRINT trial [154]. Regardless of the method used the general principles about the reliability of different measures described elsewhere apply, and the more accurate and representative the BP measurement of total load, the more likely intensive BP lowering, as demonstrated by trials, are to translate to routine clinical care. Children with CKD should receive an ACEi or ARB as 1st line treatment; in contrast to the recommendation for the general population that includes long‐acting calcium channel blockers as an option. There is a large body of evidence that ACEi and ARB are renoprotective (reduce the rate of future decline in kidney function and progression to kidney failure) when initiated in people with early CKD and particularly those with proteinuria [155, 156, 157]. There are less data to support a benefit in terms of kidney protection among those with more advanced CKD (GFR ≤ 30 mL/min/1.73m^2^) and there is currently clinical equipoise in that setting for ACEi and ARB compared to other agents; as is commonplace generally, the exclusion of children from trials limits certainty about translation of recent findings to children and diseases over‐represented in childhood (e.g., renal dysplasia and genetic kidney disease) [158, 159].

### 3.14 Treatment of Children With Diabetes Mellitus

Kidney disease is a major contributor to the burden of disease for patients with diabetes mellitus. The prevalence of both type 1 and type 2 diabetes is increasing among children and adolescents [160, 161]. The long‐term trajectory for type 1 diabetes is well described; however, early markers of kidney disease are equally or more common at presentation in children with type 2 diabetes, and thus both populations require careful attention [162, 163, 164]. Endothelial dysfunction and activation of the renin‐angiotensin aldosterone system, along with single nephron hyperfiltration, are core components of both diabetic nephropathy and hypertensive nephrosclerosis [165, 166]. ACEi or ARB is recommended as 1st line treatments in children with type 1 or type 2 diabetes when not contraindicated. This is based on the pathophysiology of diabetic kidney disease and long‐term risk of kidney disease. What data are available among adult populations indicate that the beneficial effect of ACEi or ARB on progression of CKD is not substantially modified by albuminuria status among people with DM [107, 150, 167]. Currently, there is a lack of data supporting a lower treatment initiation threshold in diabetes mellitus compared to the general population; noting the International Society for Paediatric and Adolescent Diabetes (ISPAD) Consensus Guideline follows the AAP 2017 threshold of 130/80 mmHg [107, 168]. Albuminuria is the preferred measure of urinary protein in diabetic nephropathy because lower levels of albuminuria are clearly associated with diabetic kidney disease progression and may benefit from early intervention [169, 170, 171]. The ISPAD Consensus Guideline should be followed with regards to monitoring albuminuria [107].

### 3.15 Treatment of Acute Severe Hypertension

The definition and treatment of acute severe hypertension (hypertensive crisis) in children is largely driven by expert consensus, noting that there are strong similarities between guidelines. The AAP definition of severe hypertension was endorsed (95th percentile +30 mmHg) to a limit of 180/110 mmHg per the National Heart Foundation of Australia 2016 Guideline. However, it is important to note that hypertensive emergencies and the risk of developing clinical sequalae such as posterior reversible encephalopathy syndrome (PRES) are also influenced by the rate of BP rise. PRES is more common among certain patient groups (e.g., kidney disease, including glomerulonephritis, and oncology patients) [172, 173, 174, 175]. Children with no or minimal symptoms may be treated with oral antihypertensive agents (hypertensive urgency), but those with clear symptoms related to acute hypertension (hypertensive emergency) should be treated in the intensive care unit with intravenous antihypertensive agents (see Table A11) [23, 24, 176]. Common symptoms include blurred vision, headache, vomiting and seizures in association with raised intracranial pressure and/or PRES. Hence, a thorough neurologic examination is important, including for papilloedema when possible. Infants tend to present with non‐specific symptoms (e.g., lethargy, poor feeding) and/or heart failure [177, 178, 179]. The use of intravenous agents when treating patients with symptoms allows for careful titration to ensure a controlled reduction in BP (target: < 25% in the first 6 to 8 h of treatment). A more rapid reduction in BP carries greatest risk in children with longstanding hypertension among whom vascular adaptations may lead to inadequate end organ perfusion (e.g., brain, retina, kidneys) with large drops in BP, regardless of the absolute level of BP [180]. The choice of agent will depend on local preference and medication availability, along with patient‐specific factors. Acute severe hypertension and treatment in the Australian context have recently been reviewed; see Table A11 for suggested treatment options [176].

### 3.16 Sports Participation

The absolute risk for cardiac events in children with hypertension is likely to be very low and events among children during sports participation are most commonly due to arrhythmogenic syndromes [181]. Conversely, engagement in sports is likely to benefit the BP and cardiovascular risk profile for most children with hypertension. Exclusion from sports participation also has negative impacts upon physical and mental health [182]. Physical activity in early life specifically is important, being associated with the development of motor skills, and the persistence of physical activity and sports participation among school‐aged children as a predictor of physical activity in adulthood [183, 184, 185]. For these reasons, the panel combined and adapted AAP KAS 28 and 29 to a baseline position of inclusion, in the absence of severe hypertension or significant cardiac changes (Item 30, Table 2). Children with cardiac abnormalities should be reviewed with a cardiologist. The AUSactive pre‐exercise screening tools provide further advice on broader considerations for exercise among those beginning an exercise program from a sedentary or low baseline, or when significantly upgrading exercise intensity (https://ausactive.org.au/pss‐yp).

### 3.17 Transition of Care

Item 31 (Table 2) was adapted to reflect differences in the Australian healthcare system regarding the age of transition, which typically occurs between 16 and 18 years of age. Paediatricians and families strongly believe that transition should be tailored to individual capacity and healthcare status. The principles of good transition, including preparation, communication, co‐developed transition plans, and empowering families as healthcare consumers are universal. These actions begin in early adolescence through encouraging the patient voice and fostering positive engagement with health, while supporting a graded increase in patient autonomy and responsibility prior to transfer of care. Setting patients up for lifelong care is one of the reasons why early inclusion of a GP in the care team is important for children with any chronic condition.

## 4 Specific Considerations in a Rural and Remote Context

Two‐thirds of Australia's population live in capital cities, most of which have direct access to tertiary‐level paediatric care [186]. Larger rural centres may have visiting subspecialist or general paediatric care, but this is not uniform. The distances to access specialist care for patients in many rural and remote settings may be vast, increasing the burden of care for those with chronic illness. The prevalence of risk factors such as obesity and downstream clinical events is more common in rural and remote areas [186]. This correlates with higher BP among children in rural and remote Australia compared to those living in urban centres, regardless of ethnicity [14]. It is important to balance principles of equity of access with the burden of care associated with investigations and treatments otherwise recommended in urban settings. Table 11 outlines general considerations in rural and remote settings, but individual decisions will depend on patient‐level factors such as distance from an urban centre or the pre‐test probability when ordering diagnostic tests. Children meeting the indications outlined in Table 8 should undergo further testing to identify secondary hypertension. Healthcare providers in rural and remote settings often provide care under an expanded scope of practice reflecting their specific skill set in caring for patients in these settings. Patients with severe hypertension or evidence of end organ damage should be managed in collaboration with a paediatrician.

**TABLE 11:** Considerations in rural and remote settings.

Paediatric ABPM is not routinely available in rural settings. For adolescents ≥ 16 years or ≥ 170 cm tall, ABPM can be performed and reported according to adult protocols
Home blood pressure may play a greater role in the identification and management of hypertension in remote patients, with appropriate family support
The threshold for specific investigations may differ based on the potential benefit and burden of care (e.g., performing a CT angiogram instead of direct angiography may be preferred as part of the work‐up for renovascular disease)
We should continue to advocate for improved services outside of cities, acknowledging that some components of care will necessarily be centralised to tertiary settings

*Practice point* selecting an anti‐hypertensive agent	
Cohort	Advice
All children	Start with a long‐acting calcium channel blocker or ACEi/ARB.
	Titrate to the maximum recommended dose before adding additional agents.
Adolescent females	Confirmation of contraception prior to initiation of ACEi/ARB is advised, noting the need to balance the potential teratogenicity with kidney benefits for women of reproductive age.
Diabetes	An ACEi or ARB is recommended as the initial agent.
Kidney disease	For children with glomerular proteinuria, an ACEi or ARB is recommended as the initial agent.
2nd or 3rd line agent	A diuretic (thiazide type unless advanced CKD) should be introduced as the 2nd or 3rd agent to combat reflexive salt retention.
	Single pill combinations improve adherence when multiple medications are need for control, which is common in secondary hypertension or for adolescents with essential hypertension.

Abbreviations: ABPM, ambulatory blood pressure monitoring; ACEi, angiotensin converting enzyme inhibitor; ARB, angiotensin receptor blocker; CKD, chronic kidney disease.

## 5 Improving Care for Aboriginal and Torres Strait Islander Children

Aboriginal and Torres Strait Islander peoples are the first custodians of Australia (hereafter, respectively referred to as Indigenous). The diversity of Indigenous peoples within Australia reflects the diverse landscape and > 60 000 years of continuous culture. While there have been improvements in rates of cardiovascular and kidney disease for Indigenous people in Australia, this has mirrored those of the total population, such that the gap in healthy life years and life expectancy has remained largely unchanged in recent years [187, 188]. Cardiovascular disease is estimated to be the greatest contributor to this disadvantage, and kidney disease is in the top 3 to 5 causes [187]. Hypertension is a key risk factor for both and is more common among Indigenous adults and children at a population‐level [13, 14, 15, 187]. Studies comparing the cardiovascular health of Indigenous and non‐Indigenous children in rural and remote settings have shown a smaller gap in the prevalence of hypertension [189, 190]. These differences compared to an urban environment could reflect the importance of connection to land to physical, mental and cultural health. In some parts of Australia unsafe drinking water and other environmental factors may also contribute [191]. Beyond rurality, other early life factors that account for some of the observed gap in cardiovascular and kidney health include perinatal disadvantage leading to pre‐term birth and intra‐uterine growth restriction, along with high rates of infection‐related glomerulonephritis (particularly in the north of Australia) [192, 193]. The disproportionate burden of adverse childhood experiences among Aboriginal and Torres Strait Islander children, including exposure to racism, bereavement, and family disruption rooted in the ongoing impacts of colonisation, is increasingly recognised as a structural driver of cardiovascular and metabolic health inequities [194, 195, 196]. In communities where chronic kidney disease and kidney failure among young adults are highly prevalent, chronic disease can become normalised, reducing the urgency of prevention and early intervention [197, 198]. The transition from traditional diets, which sustained low rates of cardiometabolic disease over thousands of years, to highly processed foods with significant hidden salt content has been a major contributor to the rising burden of hypertension and cardiovascular disease in Indigenous communities [199, 200]. Strategies that harness the intrinsic strength, resilience, and learnings of Indigenous people have the potential to improve outcomes for Indigenous and non‐Indigenous people. Health promotion initiatives should actively engage fathers in modelling positive health behaviours and understanding good nutrition, rather than placing this responsibility primarily on mothers [201]. Meaningful engagement of Elders is also essential, as health knowledge in many Indigenous communities is shared through distinct cultural pathways that differ from Western models of health education, and harnessing these strengthens the reach and cultural legitimacy of prevention efforts [202, 203]. Community‐led programs in Australia including arts‐health initiatives and cultural yarning have demonstrated the effectiveness of embedding cardiovascular health promotion within culturally meaningful practice [204].

## 6 Next Steps and Implementation

We envisage that most children with hypertension will continue to be referred to specialist clinics initially. However, it is also important that these clinics and bodies such as Hypertension Australia and BPOzKids continue to provide educational opportunities and outreach for general paediatricians and practitioners that facilitate the de‐centralised care of less complex patients. As an example, the older adolescent with obesity and stage I hypertension is a common clinical scenario and one which is arguably best addressed by primary care. The most important component of long‐term care of such patients is the development of a longitudinal relationship optimising opportunities to facilitate positive behavioural change, which are dependent on the patient and their individual psychosocial environment, and the available local resources/multidisciplinary interventions. In this situation, general practitioners are best placed to provide the individualised, whole of life care required. General practitioners already have significant expertise in the management of metabolic syndrome, obesity, and hypertension among older people.

There are currently limitations in the remuneration provided for components of this guideline that were considered best practice by the CAP and GSC members. Recent progress has been made regarding the use of ABPM for diagnosis in children and single‐pill combination medications. Hypertension Australia and BPOzKids will continue advocating for change and engaging relevant stakeholders to address system‐wide problems.

The Chair of the CAP will take responsibility for annual review of guideline currency relative to (a) any updates to the source AAP Guideline or relevant AAP recommendations (e.g., ABPM), (b) changes to the relevant guidelines for the Australian adult population that impact consistency with the recommendations in this guideline, (c) any new randomised controlled trials or high‐quality observational data likely to change the recommendations made.

## Funding

The authors have nothing to report.

## Conflicts of Interest

The authors declare no conflicts of interest.

## Supporting information


**Data S1:** jpc70491‐sup‐0001‐Supinfo.docx.
**Table A1:** AGREE II comparison of AAP and ESH guideline methodology and reporting.
**Table A2:** Implementation and equity considerations in key settings.
**Table A3:** AAP key action statements (KAS) that were not included in the Australian guideline.
**Table A4:** Effects of lifestyle interventions on blood pressure in children and adolescents: review of meta‐analyses (2016–2026).
**Table A5:** Project team.
**Table A6:** Guideline Steering Committee.
**Table A7:** Clinical Advisory Panel.
**Table A8:** Comparison of the Australian guideline with other international guidelines.
**Table A9:** Criteria for screening.
**Table A10:** Commonly used oral antihypertensive agents for management of chronic hypertension in children and adolescents.
**Table A11:** Oral agents used for the treatment of acute severe hypertension.

## Data Availability

Data sharing not applicable to this article as no datasets were generated or analysed during the current study.
