# Supplementary material for: Australian Guideline for the Identification and Management of Hypertension in Children and Adolescents
Source: J Paediatr Child Health. 2026 Jul 14;62(10):1822–47. doi: 10.1111/jpc.70491 (PMC13671249; doi:10.1111/jpc.70491)
Supplement: Supplementary file 1 [file JPC-62-1822-s001.docx]

Australian Guideline for the Identification and Management of Hypertension in Children and Adolescents

Supplemental Material

[1	Introduction	2](#_Toc225237559)

[2	Setup Phase	3](#_Toc225237560)

[3	Adaptation Phase	4](#_Toc225237561)

[3.1	Scope and Purpose	4](#_Toc225237562)

[3.2	Search and Screen	4](#_Toc225237563)

[3.3	Assessment	5](#_Toc225237564)

[3.3.1	Guideline Quality	5](#_Toc225237565)

[3.3.2	Guideline Currency	5](#_Toc225237566)

[3.3.3	Guideline Content and Consistency	7](#_Toc225237567)

[3.3.4	Guideline Acceptability and Implementability	7](#_Toc225237568)

[3.3.5	Decision and Adaptation Process	11](#_Toc225237569)

[4	Finalisation	13](#_Toc225237570)

[4.1.1	Consumer and User Consultation	13](#_Toc225237571)

[4.1.2	Endorsement	13](#_Toc225237572)

[4.1.3	Aftercare and Review	13](#_Toc225237573)

[5	Effects of lifestyle interventions on blood pressure	15](#_Toc225237574)

[6	DASH Eating Plan Resource Development	18](#_Toc225237575)

[7	Composition of the Guideline Committees	19](#_Toc225237576)

[8	Committee Member Disclosure of Interests	22](#_Toc225237577)

[9	Comparison with International Guidelines	23](#_Toc225237578)

[10	Criteria for Screening	31](#_Toc225237579)

[11	Pharmacological management of chronic hypertension in children and adolescents	32](#_Toc225237580)

[12	Treatment of acute severe hypertension	34](#_Toc225237581)

[13	Reviewers and organisations involved the consultation process	35](#_Toc225237582)

[14	Responses to comments from consultation	36](#_Toc225237583)

[14.1	Identification of hypertension: screening in children and adolescents; Recommendations 1-2; Table 3: Conditions associated with an increased risk of hypertension	36](#_Toc225237584)

[14.2	Defining hypertension; Recommendations 3-4; Table 4: Thresholds for the diagnosis of hypertension	41](#_Toc225237585)

[14.3	Measurement methods; Recommendation 5; Table 5: Measurement of blood pressure in children	43](#_Toc225237586)

[14.4	Out of office BP measurement; Recommendations 6-9; Table 6: Maximal out-of-office equivalents for thresholds defining hypertension	45](#_Toc225237587)

[14.5	General investigation of hypertension; Recommendations 10-12; Table 7: Sequential evaluation of childhood hypertension	47](#_Toc225237588)

[14.6	Cardiac investigations; Recommendations 13-16	49](#_Toc225237589)

[14.7	When and how to investigate for a renovascular cause; Recommendations 17-18; Table 8: Potential indications for further imaging after a normal renal ultrasound	50](#_Toc225237590)

[14.8	Albuminuria as a routine test; Recommendation 19	52](#_Toc225237591)

[14.9	Treatment targets in the general paediatric population; Recommendation 20	53](#_Toc225237592)

[14.10	Non-pharmacologic interventions; Recommendation 21; Table 9: Non-pharmacologic therapy	54](#_Toc225237593)

[14.11	Pharmacologic management" (page 16)- Recommendation 22 (Table 2) - Table 10: Criteria for pharmacologic antihypertensive therapy	57](#_Toc225237594)

[14.12	The role of ABPM in assessing treatment efficacy; Recommendation 23	59](#_Toc225237595)

[14.13	Treatment of children with hypertension and kidney disease; Recommendations 24-28	60](#_Toc225237596)

[14.14	Treatment of children with hypertension and diabetes mellitus; Recommendation 29	61](#_Toc225237597)

[14.15	Treatment of acute severe hypertension; Recommendation 30	61](#_Toc225237598)

[14.16	Sports participation; Recommendation 31	62](#_Toc225237599)

[14.17	Transition of care; Recommendation 32	63](#_Toc225237600)

[14.18	Specific considerations in a rural and remote context; Table 11: Considerations in Rural and Remote Settings	64](#_Toc225237601)

[14.19	Improving care for Aboriginal and Torres Strait Islander children" (page 23)	64](#_Toc225237602)

[14.20	Any other parts of the manuscript; Table 1: Summary of key principles from the Australian guideline; The Supplement	65](#_Toc225237603)

[15	References	69](#_Toc225237604)

# 1 Introduction

To date, childhood and adolescence (i.e. ages < 18 years) have not been addressed by existing hypertension and cardiovascular risk guidelines in Australia [1, 2]. Given this significant gap, members of the BPOzKids network and Hypertension Australia identified the need for an Australian paediatric hypertension guideline and published the rationale for a guideline development initiative [3].

Given the limited resources, the existence of several reputable international guidelines [4-7], and the low likelihood of substantial new evidence in the field since the publication of these guidelines, the decision was made to adapt one or more existing guidelines to the Australian context. This guideline was therefore developed using the ADAPTE Process [8], which specifies three phases: 1) Set-up, 2) Adaptation, and 3) Finalisation.

# 2 Setup Phase

The initial set-up phase involved formation of a Project Team (Table A5) under the governance of the Murdoch Children’s Research Institute (Melbourne, Australia). The Project Team then formed a Guideline Steering Committee (GSC), consisting of senior multidisciplinary experts and consumer representatives, whose expertise and representation are outlined in **Table A6**. Terms of Reference were developed by the Project Team, which outlined the context, role of the GSC members, time commitment, and governance structure, along with conflict of interest, confidentiality and authorship policies. The GSC members were required to complete a Declarations of Interest Form^[1]^, and any conflicts of interest were identified and (where necessary) a management plan created.

Through consensus discussion at regular meetings during 2023-2024, the GSC established the following:

- Guideline Topic: Clinical diagnosis and management of hypertension
- Population: All paediatric ages up to 18 years, with comment on the young adult transition period
- Guideline Adaptation Process (ADAPTE)
- List of Endorsement Bodies to approach
- Guideline Authorship policy
- Dissemination Plan
- Implementation Plan, discussed and developed throughout the guideline adaptation process

In May 2023, the GSC and Project Team distributed an Expression Of Interest call for membership of a Clinical Advisory Committee (CAP), whose task would be to conduct the guideline adaptation and vote on recommendations. Members of the CAP (**Table A7**) were identified through EOI applications or through direct invitation by the GSC, to ensure coverage of all relevant disciplines, as well as gender diversity and geographical representation. Terms of Reference were developed for the CAP and each member completed a Declaration of Interest form. No conflicts of interest requiring a management plan were identified.

# 3 Adaptation Phase

## 3.1 Scope and Purpose

The health questions defined by the American Academy of Paediatrics 2017 guideline [4] were adopted, namely:

1. How should systemic hypertension (eg, primary hypertension, renovascular hypertension, white coat hypertension, and masked hypertension) in children be diagnosed, and what is the optimal approach to diagnosing hypertension in children and adolescents?
2. What is the recommended workup for paediatric hypertension? How do we best identify the underlying aetiologies of secondary hypertension in children?
3. What is the optimal goal systolic blood pressure (SBP) and/or diastolic blood pressure (DBP) for children and adolescents?
4. In children 0 to 18 years of age, how does treatment with lifestyle versus antihypertensive agents influence indirect measures of cardiovascular disease risk, such as carotid intima-media thickness, flow mediated dilation, left ventricular hypertrophy, and other markers of vascular dysfunction?

Two additional questions were added that are relevant to the Australian context:

5. Should modifications be made to recommendations to facilitate the care of children in resource limited (e.g. remote) settings?
6. Are all of the recommendations relevant to all groups in Australia, including First Nations Peoples?

## 3.2 Search and Screen

A systematic literature search was conducted to identify existing guidelines that address these health questions. Details of this process will be published separately. Briefly, the major guidelines identified were:

- American Academy of Paediatrics (AAP) (2017) [4]
- European Society of Hypertension paediatric guideline (2016) [5] and adult guideline with paediatric section (2023) [9]. Consensus statements from the European Society of Cardiology (2022)[10], and HyperChildNET and the European Academy of Pediatrics (2023) [11, 12] were also noted.
- Hypertension Canada (2020) [6]
- Japanese Society of Hypertension (2019) [7]

The Canadian and Japanese guidelines were published as sections within a broader guideline covering adults, and therefore contained insufficient detail to adapt. A number of other guidelines were identified, but were themselves either explicit or apparent adaptations or summaries of the AAP guidelines (e.g. 7^th^ Brazilian Guideline of Arterial Hypertension [13], Royal Children’s Hospital Clinical Practice Guideline [14]). The AAP and European Society of Hypertension guidelines were therefore identified as the best candidates for adaptation.

The Appraisal of Guidelines for Research & Evaluation II (AGREEII) tool [15] was used to evaluate the AAP and European guidelines. Three members of the CAP independently scored these guidelines and average scores for each of the 23 domains were then summed to provide a final summary score, for which the AAP guideline scored 143 and the ESH guideline scored 109. Detailed results will be published separately. In brief, the AAP guideline scored 70-100% and the European guideline scored 35**-**92% across the six domains (Scope and Purpose, Stakeholder Involvement, Rigour of Development, Clarity of Presentation, Applicability, and Editorial Independence, see Table A1). Based on this assessment, and after discussion amongst the GSC and CAP committees, the consensus opinion was to primarily adapt the AAP guideline, whilst also taking into consideration any diverging recommendations in the other guidelines (European, Canadian, Japanese).

## 3.3 Assessment

### 3.3.1 Guideline Quality

The AAP guideline was assessed for quality using the AGREEII tool, as described in the previous section, with very high inter-rater consistency (96% of questions had a range of 0 or 1 across three raters, on a 7-point scale). Scores were excellent for domains ‘Scope and Purpose’ (98%), ‘Clarity of Presentation’ (94%), ‘Editorial Independence’ (100%); very high for domains ‘Rigour of Development’ (86%) and ‘Applicability’ (80%); and acceptable for the domain ‘Stakeholder Involvement’ (70%). The AAP guideline was therefore deemed to have a high degree of quality, suitable for adaptation.

### 3.3.2 Guideline Currency

The AAP guideline was published in 2017, raising the possibility that relevant new evidence may have been generated in the last 7 years. This was investigated in several ways.

First, the evidence referenced in other recent guidelines was reviewed for relevance to the AAP Key Action Statements being reviewed (snowballing).

**Table A1.** AGREE II Comparison of AAP and ESH Guideline Methodology and Reporting

Scores are the mean of three reviewers who scored each guideline independently and then conferred to minimise the chance of information being missed across the multiple documents reviewed (ESH 2023, ESH 2016, AAP 2017 main document and methodology supplements).

AAP: American Academy of Pediatrics; AGREE II: Appraisal of Guidelines for Research & Evaluation; ESH: European Society of Hypertension.

Second, the CAP consulted with a globally renowned expert and the lead author of the AAP guideline (Prof Joseph Flynn, Seattle Children’s Hospital) to ascertain whether he was aware of new evidence that might affect the recommendations. This led to updates of recommendations relating to ambulatory blood pressure monitoring. The CAP also consulted with a leading international expert in neonatal hypertension (A/Prof Janice Dionne, BC Children’s Hospital, Vancouver) regarding recent evidence or guidance in this sub-population. No recommendations pertinent to the neonate were identified as needing revision.

Third, focussed searches for randomised controlled trials or high-quality observational studies were conducted for any new evidence regarding the use of home BP, and pharmacological treatment for paediatric hypertension.

The new evidence was considered by the CAP when formulating the recommendations.

### 3.3.3 Guideline Content and Consistency

Where multiple guidelines form the basis for adaptation, the ADAPTE process involves assessing their content and consistency through recommendation matrices. However, since the CAP primarily based adaptation on a single guideline (AAP), and since many of the recommendation specifics are based on expert consensus, formal recommendation matrices were not developed. However, a comparison of key recommendation areas from the AAP, European, Canadian and Japanese guidelines was performed. Section 9 of this Appendix contains a summary of this comparison, and highlights differences and similarities with the new Australian recommendations.

### 3.3.4 Guideline Acceptability and Implementability

Acceptability and implementability were discussed extensively throughout the guideline development process. An implementation plan was formulated in parallel with the guideline development, drawing from the updated Consolidated Framework for Implementation Research (CFIR). The GSC and CAP held a joint day-long workshop and discussed the broad “innovations” (what will be implemented) and the “inner settings” (contexts where the innovations could be implemented). The committee members considered the following questions:

1. What are the facilitators for [innovation] in [setting] and what implementation ideas would lead to the easiest wins?
2. What are the barriers to implementing [innovation] in [setting]?
3. What positive or negative equity considerations may exist for [innovation] in [setting]?

Four broad innovations were considered in relation to the patient journey:

1. Screening for hypertension
2. Ambulatory blood pressure monitoring (confirming diagnosis)
3. General diagnostic work-up and management (usually for primary hypertension)
4. Specialist diagnostic work-up and management (usually for secondary hypertension)

Settings were ranked (using a confidential poll) from the most to least promising regarding acceptability and implementation (Table A2).

**Table A2.** Implementation and equity considerations in key settings

| **General Practice** | |
| --- | --- |
| Innovations | Blood pressure screening (+++)  Ambulatory blood pressure monitoring (++)  General diagnostic work-up and management (++) |
| Facilitators and Opportunities | Practice nurses – availability/ability to follow standard procedures  Potential for recall program  Parents can play a role in asking for BP screening  One-pager decision tool  Long-standing relationship with patient  Introduction of a well-child check  Software / GP resource updates/integrations  GP/nurse education / CPD modules |
| Barriers | Limited recognition of the issue  Competing priorities  Limited workforce / time  Children visit when unwell |
| Equity positives | Access is easier than for paediatricians or hospitals |
| Equity negatives | CALD groups and those with poor health literacy are likely to have lower rates of screening  Rural/remote access may be limited |
| **Dedicated Paediatric Hypertension Service** | |
| Innovation | Ambulatory blood pressure monitoring (+++)  General diagnostic work-up and management (+++) |
| Facilitators and Opportunities | One-stop shop, all investigations in one place (more convenient)  Potential for telehealth  Expertise - trusted source of information  Opportunities for research (e.g. EMR-embedded data collection)  Co-location with other services  Could develop decision support tools, patient resources etc |
| Barriers | Limited hospital space  Potential for poor attendance at appointments  Funding and reimbursement |
| Equity positives | Shared care model with primary care  Use of communications/technology |
| Equity negatives | Geographic inequity - limited access for rural/remote families, first nations and CALD |
| **School-based Programs** | |
| Innovations | Blood pressure screening (++)  Ambulatory blood pressure monitoring (-) |
| Facilitators and Opportunities | Captive audience / potential for high coverage  Positive attitudes to promoting health / education by school staff  Children are interested to learn about their heart  Could be integrated into learning programs on health, science or PE  School nurses  Normalises the experience when everyone is involved  Opportunities to partner with existing school-based programs  Opportunities to make screening fun and engaging  Good chance to achieve repeat measures |
| Barriers | Resource intensive  Multiple school systems to navigate (private, state, Catholic)  Communication with parents  Would require system change and culture change  School non-attendance may be highest in those with highest risk  Rural/remote access may be especially resource intensive and some use online schooling |
| Equity positives | Highest chance for serving CALD groups and those with poor health literacy  Potential to access rural/remote areas  Potential for follow-up |
| **General Paediatrics** |  |
| Innovations | Blood pressure screening (-)  Ambulatory blood pressure monitoring (+)  General diagnostic work-up and management (+) |
| Facilitators and Opportunities | Established relationship with the patient  Highly motivated to support health in children  Nursing and allied health links |
| Barriers | Limited time, very stretched workforce  Very long waiting times, high demand  Lack of familiarity with guidelines  Competing clinical priorities  Insufficient time to perform repeat measures  Challenging to justify treating apparently well children vs treating sick children |
| Equity positives | (none identified) |
| Equity negatives | Access is limited |
| **Pharmacies** |  |
| Innovation | Blood pressure screening (+) |
| Facilitators and Opportunities | Highly accessible  Screening with kiosk systems |
| Barriers | Few validated devices available for this setting  Dependent on parents to initiate |
| Equity positives | Widely accessible |
| Equity negatives | CALD groups and those with poor health literacy may be less likely to access |
| **Paediatric Hospital Clinics** | |
| Innovation | Blood pressure screening (-) |
| Facilitators and Opportunities | Healthcare assistants available to measure BP alongside height and weight  Child-friendly environment and child-focused staff  Expertise and workforce present |
| Barriers | Inadequate equipment and workflow  Limited time  Siloed work areas  May not be appropriate use of doctor’s time  Maintenance of equipment |
| Equity positives | (none identified) |
| Equity negatives | Poor accessibility |

Settings ranked as most promising (+++), second-most promising (++), third-most promising (+) or ranked fourth or lower (-) to start implementation of the specified innovation. CALD, Culturally And Linguistically Diverse

As reflected in Table A2, the committee considered general practice or school programs to be the most promising avenues for implementing population-level blood pressure screening in Australian children and adolescents. A dedicated clinic or general practice were considered the most viable settings for confirming hypertensive status with 24-hour ambulatory blood pressure monitoring and for diagnostic work-up and clinical management of uncomplicated primary hypertension. As per current practice, patients with complex co-morbidities or secondary hypertension require referral to nephrology, cardiology and/or endocrinology specialists.

### 3.3.5 Decision and Adaptation Process

The Clinical Advisory Panel discussed the 30 Key Action Statements (KAS) from the AAP guideline, considering factors including 1) currency with respect to recent evidence, 2) relevance to and acceptability in the Australian healthcare system, 3) implementability, and 4) special considerations related to rural/remote contexts and First Nations communities.

#### Modified Delphi Process

A modified Delphi process was conducted using custom and private REDCap questionnaires. Each page related to one or a cluster of Key Action Statements, and contained a summary of each KAS with

a) the AAP recommendation wording,

b) level of evidence,

c) an explanation of the evidence/rationale for the KAS provided in the AAP guideline,

d) an interpretation of the KAS in the Australian context,

e) an outline of any new evidence that might justify modifying the recommendation,

f) a copy of the KAS table from the AAP guideline (detailing evidence quality, benefits, risks/harms/costs, balance of benefit-harm assessment, intentional vagueness, role of patient preferences, exclusions and strength of recommendation), and

g) key references as attachments.

CAP members were asked: “Do you agree that we adopt this Key Action Statement without modification?” using a 7-point Likert scale (7=Strongly Agree to 1=Strongly Disagree). A free text field was provided to allow for comments. If the response was not “Strongly Agree” or “Agree”, members were also asked whether they recommend modifying or rejecting the KAS.

KAS items that attained a median score of 6 or more were considered accepted, although free text comments were also taken into consideration and discussed by the panel. Items to be adapted were then distributed in subsequent Delphi rounds, where proposed revised wording was contrasted with the original AAP wording, and the question posed: “Do you agree that we adopt the revised Key Action Statement?” The process was repeated until all items were accepted. Table A3 lists the AAP Key Action Statements that were not included in the Australian guideline, along with the reason for exclusion. Further explanation is provided in the main guideline document.

**Table A3.** AAP Key Action Statements (KAS) that were not included in the Australian guideline

| **Removed Key Action Statement** | **Reason** |
| --- | --- |
| Children and adolescents with suspected white coat hypertension should undergo ABPM. Diagnosis is based on the presence of mean SBP and DBP <95th percentile and SBP and DBP load <25%. | Diagnosis based on the load component of ABPM no longer recommended due to new evidence; remainder of recommendation is covered elsewhere. |
| Left ventricular hypertrophy should be defined as left ventricular mass >51 g/m^2.7^ (boys and girls) for children and adolescents older than age 8 years and defined by left ventricle mass >115 g/body surface area for boys and left ventricular mass >95 g/body surface area for girls. | Optimal definition of LVH is controversial. Consensus was to rely on expert clinical interpretation based on all available information. |
| Routine testing for microalbuminuria is not recommended for children and adolescents with primary hypertension. | Removed to maintain consistency with other guidelines and avoid confusion for general practitioners |
| Children and adolescents with hypertension may participate in competitive sports once hypertensive target organ effects and cardiovascular risk have been assessed. | Multiple statements regarding sport participation combined and adapted into a single statement. |

#### Recommendation Wording

To facilitate clarity, where possible, recommendations were formulated according to the standard format: “In <population>, perform <intervention>, with <clarifiers>”. Most recommendations that were adopted from the AAP guideline were also re-worded according to this standard format, without changing the substance of the recommendation.

# 4 Finalisation

### 4.1.1 Consumer and User Consultation

Consumers were represented on the Guideline Steering Committee and consumer input was obtained from a wide range of stakeholders for the accompanying resources, including the “Kidney Crew” consumer advisory group (childhood kidney disease lived experience) at the Murdoch Children’s Research Institute.

Feedback was also provided by some members the Adult Hypertension Guideline expert group from the National Heart Foundation, which includes consumers.

A yarning circle was held with 3 Borloo/Perth Elders from the Healthy Lifestyle Program Cultural Advisory Group, facilitated by Stephen Paull and Yvonne Anderson of Curtin University to refine and expand the section on ‘Improving care for Aboriginal and Torres Strait Islander children’.

Consumer input was obtained from more than 30 stakeholders (dietitians, hypertension nurses, nephrologists, lipids nurses, parents, adolescents and GPs) for the DASH eating plan resources that accompany the guideline.

The consultation process included an open call for reviewers via social media as well as targeted invitations to peak medical bodies, consumer/community organisations and individual clinicians with relevant expertise. With the consent, a full list of reviewing individuals and organisations is included in Section 13, while comments provided on the consultation draft and the responses from the guideline committee are published in full in Section 14.

### 4.1.2 Endorsement

At the time of writing, the guideline has been endorsed by the following organisations:

- Australia New Zealand Paediatric Obesity Network
- Australia and New Zealand Paediatric Nephrology Association
- Cardiac Society of Australia and New Zealand
- Hypertension Australia
- Kidney Health Australia
- National Heart Foundation
- Royal Australasian College of Physicians

### 4.1.3 Aftercare and Review

The Chair of the CAP will take responsibility for annual review of guideline currency relative to a) any updates to the source AAP Guideline or relevant AAP recommendations (e.g. ABPM), b) changes to the relevant guidelines for the Australian adult population that impact consistency with the recommendations in this guideline, c) any new randomised controlled trials or high-quality observational data likely to change the recommendations made.

# 5 Effects of lifestyle interventions on blood pressure

Table A4 summarises results of meta-analyses published from 2016-2026 that examined the effects of lifestyle interventions on blood pressure in children and adolescents. Although there is significant heterogeneity with respect to intervention methodology, overall physical activity (PA) interventions alone in a general population reduced BP by 2-4 mmHg in studies that incorporated adolescents [16-19], but had no or weak effect in younger children [19, 20]. However, larger effects (up to 6-9 mmHg for SBP) were generally reported in younger children with overweight/obesity or elevated blood pressure [21, 22], and when PA was combined with nutrition and behaviour change intervention [20]. Mediterranean diet was associated with ~5 mmHg (SBP) and ~2 mmHg (DBP) improvement [23], and there was evidence that high consumption of sodium and sugar-sweetened beverages contributes to high BP [24-26], with high vs low salt intake associated with an odds ratio of 2.0 for high BP [26]. There was weak evidence of no effect of Vitamin D supplementation on SBP in non-hypertensive children and adolescents [27].

**Table A4.** Effects of lifestyle interventions on blood pressure in children and adolescents: review of meta-analyses (2016-2026)

| **Study** | **Study design** | **Study characteristics** | **Findings** |
| --- | --- | --- | --- |
| Yuan, Shao [16] | Meta-analysis evaluating relationship between PA dose and BP lowering effect. Interventions >4 weeks. | 22 RCTs, n=3456, 6-24 years old  Most participants had elevated BP or hypertension  Mean intervention time: 21.6 weeks | Mean effect (mmHg):  SBP: −4.1 (−6.1, −2.1)  DBP: −2.0 (-3.1, −0.8)  Maximal effect at 700 MET-minutes per week  SBP: Larger effect in overweight (−6.1), obesity (−7.4), and adolescents (−7.1) |
| Tozo, Tadiotto [17] | Meta-analysis of the effect of different exercise programs on BP in overweight children and adolescents | 22 RCTs and nRCTs, n=1,125, 6-17 years old | Mean SBP / DBP effect (mmHg):  MICT: −3.1 (−5.2, −1.0) / −3.5 (−5.3, −1.8)  HIIT: −4.1 (−7.7, −0.5) / −4.5 (−7.7, −1.3)  CT: −0.1 (−0.4, 0.3) / −2.2 (−4.6, 0.3)  Other: −1.8 (−7.5, 3.9) / −1.3 (−4.6, 1.9) |
| Hassan, Zhou [20] | Network meta-analysis of RCTs and controlled quasi-experimental studies using physical activity or exercise as the major influence | 27 studies, n=15,220, 6-12 years old | Mean SBP effect (mmHg):  PA only: +0.6 (0.1, 1.2)  PA + Education: −0.3 (−1.2, 0.6)  PA + Nutrition: −0.4 (−1.1, 0.2)  Multiple components: −1.4 (−1.9, −0.8)  PA + Nutrition  + Behaviour change: −8.6 (−11.4, −5.8) |
| Hernandez-Martinez, Perez-Carcamo [18] | Meta-analysis of RCTs evaluating effect of exergaming (active video games) on BP in children and adolescents | 4 studies (for BP outcome), n=143, mean age 13.9 years | Effect size**:  SBP: −0.45 (−0.12, −0.78)  DBP: −0.08 (−0.83, 0.667) |
| Lopez-Gil, Garcia-Hermoso [23] | Meta-analysis of RCTs assessing the effect of Mediterranean diet | 9 RCTs, n=577, 3-18 years old | Mean SBP / DBP effect (mmHg):  −4.8 (−9.0, −0.5) / −1.9 (−4.0, 0.2) |
| García-Hermoso, Alonso-Martinez [21] | Meta-analysis of RCTs evaluating effectiveness of exercise interventions on BP in children <6 years old | 4 RCTs, n=7,843, 1-6 years old  General population - could include overweight/obesity but not samples with diagnosed medical condition | Mean Hedges’ g (overall):  SBP: −0.17 (−0.41, 0.07)  DBP: −0.14 (−1.35, 0.18)  Sub group analysis lean vs obese in 1 study (SBP / DBP change, mmHg):  Lean (n=31): −2.91 ± 4.3* / −3.9 ± 6.2*  Obese (n=21): −­8.3 ± 6.6* / −2.9 ± 8.3 |
| Abboud [27] | Meta-analysis of Vitamin D supplementation intervention studies | 3 RCTs, 2 nRCTs (low quality), 4 to 18 years old  Mostly healthy, non-hypertensive subjects | Mean SBP / DPB effect (mmHg):  2 nRCTs: 0.4 (0.9, 1.7) / −1.9 (−3.0, −0.7)  3 RCTs: −2.0 (−5.1, 1.0) / 0.0 (−1.1, 1.1) |
| Farhangi, Nikniaz [24] | Meta-analysis of observational studies evaluating the association between sugar-sweetened beverages (SSB) and BP in children and adolescents | 14 studies, n=93,873, 5 to 22 years old  Most studies performed in healthy children/adolescents | High SSB consumption effect:  SBP / DBP: 1.7 (1.0, 2.3) / 0.3, (−0.1, 0.8)  Odds of hypertension for high vs low SSB consumers: 1.36 (1.15, 1.63) |
| Rios-Leyvraz, Bloetzer [25] | Meta-analysis of studies assessing the association between sodium intake and BP in children with clinical conditions | 16 studies, n=2902, 0 to 18 years old  Conditions: Elevated BP (10 studies), Familial hypertension (2), other (4) | Mean effect in studies including children with elevated BP (mmHg/g sodium per day):  SBP / DBP: 6.3 (2.9, 9.6) / 3.5 (1.2, 5.7) |
| Leyvraz, Chatelan [26] | Meta-analysis of experimental and observational studies on sodium intake in children and adolescents | 85 studies, n=58,531, 0 to 18 years old  General population | Increase per additional gram of salt intake per day:  SBP: 0.8 mmHg  DBP: 0.7 mmHg  Odds of high BP for high vs low salt consumption: 2.00 (1.38, 2.62) |
| Pozuelo-Carrascosa, Cavero-Redondo [19] | Meta-analysis of RCTs of school-based, PA-only interventions | 19 RCTs, n=11,988, 3-12 years old  General school population | Effect size:  SBP: −0.14 (−0.31, 0.03)  DBP: −0.21 (−0.42, −0.01)  Stronger effect with increasing age |
| Oosterhoff, Joore [22] | Meta-analysis assessing the impact of school-based lifestyle interventions on BP | 23 RCTs, n=72,934, 4-12 years old  General school population  Mix of PA, Education, Diet, & multi-component interventions | Effect size:  SBP: −0.151 (−0.253, −0.049)  DBP: −0.103 (−0.206, 0.001)  Stronger effect with increasing age, higher baseline BP, longer intervention time, and auscultatory measurement |

Data presented as mean (confidence interval), effects expressed in mmHg where possible, otherwise as standardised effect sizes (SMD), unless otherwise stated. An SMD of −0.1 corresponds to 1.0 mmHg benefit for a BP group standard deviation of 10 mmHg.

Abbreviations: BP, blood pressure, CT, combined training; DBP, diastolic blood pressure; HIIT, high-intensity interval training; MICT, moderate intensity continuous training; PA, physical activity; nRCT, non-randomised controlled trial; RCT, randomised controlled trial; SBP, systolic blood pressure, SSB, sugar-sweetened beverages

* p < 0.05

# 7 DASH Eating Plan Resource Development

A working group consisting of two paediatric dietitians, two researchers (one a dietitian, one a public health nutritionist) was formed to develop a DASH dietary advice resource for use by general practitioners and health care professionals to support the management of high blood pressure in children. A scoping process was completed to identify existing usable resources. This included grey literature searches (i.e. Google and professional databases and organisations) and review of available DASH diet smartphone applications [28], as it is recognised that applications may engage adolescents to achieve positive dietary changes [29]. Initially a generalised Google search was conducted using search terms DASH and child* or kid. An advanced Google search subsequently specified restrictions to domains .gov, .org and .edu. Further restrictions to PDF documents and then Australian sites (.au) were applied to narrow down to more consumer-friendly resources that could potentially be distributed within the Australian health-care setting. Across these searches, the first five pages of returned results were investigated. Resources retrieved came from various health and medical institutions and peak bodies, and universities and were collated in an excel spreadsheet.

No resources specific for following a DASH eating plan for children within the Australian setting were found. The search returned a relatively limited number of resources specific to children, instead many resources provided more generic information on the DASH eating plan tailored to adults. Those that did focus on specific requirements for children (e.g. DASH food group recommendations adjusted for reduced energy intakes or family friendly tips and advice) were from the USA and used context specific language for the US food environment. Searching across professional online databases (e.g. PEN Nutrition) and relevant Australian health organisations (e.g. Queensland Health NEMO, Heart Foundation, Baker Institute) yielded general educational information and fact sheets related to the topic (e.g. salt reduction, eating for a healthy heart and supermarket shopping guides for general healthy eating), but nothing specific to support adoption of a DASH eating plan during childhood. Finally, a recent review of available DASH diet apps [28] identified one app to be best in terms of quality, privacy, theoretical underpinning and evidence of effectiveness [28], however, none were designed for children and therefore not suitable for recommendation within this project. Given the potential for apps to incorporate behaviour change theory to support dietary changes (e.g. goal setting, tracking of food intake) future work could consider the feasibility of DASH eating plan apps for adolescents.

Due to the lack of ready-to-use resources for supporting DASH diet adoption in Australian children diagnosed with high blood pressure, a brief, printable educational resource was developed for use in the Australian setting. The resource was informed by existing materials and evidence on the DASH diet’s effectiveness in children and adolescents [30-33]. The resource was developed to align with screening age checkpoints recommended in the guidelines, 7-9 years and 13-15 years. The educational resource provides a brief overview of the DASH Eating Plan. However, recognizing the complexity of the eating plan (e.g. suggested changes across multiple food groups), dietary advice was simplified to focus on dietary changes in three key food groups: increased fruit, low-fat dairy and nuts or seeds. This is combined with advice on reducing sodium intake. Importantly, small changes within these food groups and associated DASH nutrients, achieved by free-living adolescents with high blood pressure educated on a DASH eating plan, have been shown to significantly reduce blood pressure BP [30, 31]. Behavioural change theory is incorporated, with goal setting actions to support relevant dietary changes.

# 8 Composition of the Guideline Committees

**Table A5.** Project Team

| **Name** | **Primary Affiliation** | **Role** |
| --- | --- | --- |
| A/Prof Jonathan Mynard | Murdoch Children’s Research Institute, VIC | Project Lead, BPOzKids Convenor |
| Dr Tanya Medley | Murdoch Children’s Research Institute, VIC | Project Co-lead |
| Dr Nick Larkins | Perth Children’s Hospital, WA | Clinical Advisory Panel Chair |
| A/Prof Cathy Quinlan | Royal Children’s Hospital, VIC | Guideline Steering Committee Chair |
| Mr Jonathan Glenning | Murdoch Children’s Research Institute, VIC | Literature reviews and writing assistance |
| Dr Freya Sheeran | Murdoch Children’s Research Institute, VIC | General research assistance |
| A/Prof Kristy Bolton | Deakin University, VIC | DASH diet resource working group |
| Dr Carley Grimes | Deakin University, VIC | DASH diet resource working group |
| Arielle Hyland | Perth Children’s Hospital, WA | DASH diet resource working group |
| Dr Sherly Li | Murdoch Children’s Research Institute, VIC | DASH diet resource working group |

**Table A6.** Guideline Steering Committee

| **Name** | **Primary Affiliation** | **Expertise / Representation** |
| --- | --- | --- |
| A/Prof Catherine Quinlan (Chair) | Royal Children’s Hospital, VIC | Paediatric Nephrology |
| Ms Amanda Black | None, WA | Parent consumer representative |
| Dr Swasti Chaturvedi | Sydney Children's Hospital, NSW | Paediatric Nephrology, First Nation’s Health |
| A/Prof Malcolm Clark | University of Melbourne, VIC | General Practice, RACGP representative |
| Dr Genevieve Gabb | Acute and Urgent Care, Central Adelaide Local Health Network | Adult General and Cardiovascular Medicine |
| Dr Nick Larkins | Perth Children’s Hospital, WA | Paediatric Nephrology, Clinical Epidemiology |
| Dr Sarah McNab | Royal Children’s Hospital, VIC | General Paediatrics |
| Dr Tanya Medley | Murdoch Children’s Research Institute, VIC | Guideline development methodology |
| Dr Anastasia Mihailidou | Royal North Shore Hospital, NSW | Hypertension Services and Research |
| A/Prof Jonathan Mynard | Murdoch Children’s Research Institute, VIC | Paediatric hypertension research, blood pressure measurement, BPOzKids, Hypertension Australia |
| Mrs Manjuri Pathak | None, WA | Parent consumer representative |
| Prof Markus Schlaich | Royal Perth Hospital, WA | Adult Nephrology and hypertension research, Hypertension Australia |
| Dr Christine Jeffries-Stokes | Rural Clinical School, University of Western Australia, WA | Paediatrician, rural/remote settings, First Nations health |

**Table A7.** Clinical Advisory Panel

| **Name** | **Primary Affiliation** | **Expertise / Representation** |
| --- | --- | --- |
| Dr Nick Larkins (Chair) | Perth Children’s Hospital, WA | Paediatric Nephrology, Clinical Epidemiology |
| Dr Siah Kim | The Children' Hospital at Westmead, NSW | Paediatric Nephrology, Public Health, First Nations Health |
| Dr Sheena Kaul | Royal Children’s Hospital, VIC | Paediatric Nephrology, RACP Representative |
| A/Prof Steven McTaggart | Children's Health Queensland, QLD | Paediatric Nephrology, Medical Services Leadership |
| Dr David Metz | Royal Children’s Hospital, VIC | Paediatric Nephrology, Clinical Pharmacology |
| Dr Jennie Monin | Sydney Kids Clinic, NSW | General Paediatrics |
| Prof Mark Nelson | University of Tasmania, TAS | General Practice, cardiovascular risk |
| Dr Rochelle Sleaby | University of Melbourne, VIC | General Practice |
| Dr Remi Kowalski | Royal Children’s Hospital, VIC | Paediatric Cardiology |
| Ms Lisa Kalman | National Heart Foundation, VIC | Guideline development and implementation, National Heart Foundation Representative |
| A/Prof Rae-Chi Huang | Telethon Kids Institute, WA | General Paediatrics, cardio-metabolic disease, childhood obesity |
| Prof Derek Roebuck | Perth Children’s Hospital, WA | Paediatric Interventional Radiology, Renovascular Disease |
| A/Prof Jonathan Mynard (Project Lead, non-voting) | Murdoch Children’s Research Institute, VIC | Paediatric hypertension research, blood pressure measurement, BPOzKids, Hypertension Australia |
| Dr Tanya Medley (Project co-lead, non-voting) | Murdoch Children’s Research Institute, VIC | Guideline development methodology |

# 9 Committee Member Disclosure of Interests

Dr Genevieve Gabb declared prior participation on an advisory board and receiving speaker fees to Novartis; and paid travel or receipt of meals from Asia-Pacific Congress of Cardiology, Novartis and Amgen.

Dr Siah Kim declared relationships with organisations with financial links or affiliations with industry groups which stand to benefit from or may be affected by guideline recommendations, and which advocate known industry or policy positions: member of International Pediatric Nephrology Association Best Practice Committee, Transplantation Society of Australia and New Zealand Scientific Program and Education Committee, KDIGO Anaemia Guideline working group, and Australia and New Zealand Society of Nephrology Quality and Indicators and Registries Sub-Committee.

Dr Anastasia Mihailidou declared receipt of honorary and paid travel for delivering education sessions for Medtronic and podcast.

A/Prof Jonathan Mynard declared receipt of research grants/contracts from Uscom and Tournicare; consulting fees from Tournicare, Brain Protection Company, Baxter Healthcare and Rockley Photonics; license fees from Masimo Corporation; board membership for Hypertension Australia, and leadership of the BPOzKids network.

Prof Mark Nelson declared that he had a relationship with organisations which advocate known industry or policy positions (Hypertension Australia, Cardiac Society of Australia and New Zealand, National Heart Foundation of Australia and Stroke Foundation guideline and research committees)

A/Prof Catherine Quinlan declared receiving honoraria from Academic CME for delivering online tutorials.

Prof Derek Roebuck declared that he had a relationship with organisations which advocate known industry or policy positions (Secretary to the Australian and New Zealand Society of Paediatric Radiology and member of Interventional Radiology Society of Australasia)

Prof Markus Schlaich declared research grants from NHMRC, NHF, DRWA, Medtronic, Recor, and Abbott Boehringer Ingelheim; advisory board participation for Medtronic, Abbott, Viatris, and Idorsia (related to Renal denervation, Hypertension, sympathetic nervous system, and endothelin antagonists); speaker fees/honoraria from Medtronic, Abbott, Novartis, Merck, and Servier; and paid travel from Medtronic, Idorsia, and Merck. He also declared Relationship with organisations with financial links or affiliations with industry groups which stand to benefit from or may be affected by guideline recommendations (President of Hypertension Australia, Co-chair of the National Hypertension Taskforce, and Director of Western Australia Cardiovascular Research Alliance).

All other committee members declared no interests.

# 10 Comparison with International Guidelines

**Table A8.** Comparison of the Australian guideline with other international guidelines

|  | Australia (2026) | AAP (2017) | ESH (2016) | Canada (2020) | Japan (2019) |
| --- | --- | --- | --- | --- | --- |
| Frequency and age for BP screening in the absence of other risk factors | Screen at approximately 7-9 and 13-15 years of age (may be done opportunisti­cally at other ages) | Annually from 3 years | Every 2 years from the age of 3 years | Regularly from 3 years | Annually (no minimum age specified) |
| BP measurement in the presence of risk factors | From 3 years of age at every healthcare encounter | Same | NSR | NSR | NSR |
| Simplified screening thresholds for high blood pressure | Ages 6-11 yr: 120/80 mmHg  Ages 12-17: 130/85 mmHg | See Table 6 of AAP guideline (age-based threshold for each year integer to 13 years of age, stratified by sex based on shortest child in the relevant strata) | NSR | Same | Preschool: 120/70  Grade 1-3: 130/80  Grade 4-6: 135/80  Junior high (girls): 135/80  Junior high (boys): 140/85  High school: 140/85  (mmHg) |
| Thresholds for defining elevated blood pressure (‘prehyperten­sion’ or ‘high-normal’ BP) | 1-17 yr: 90^th^ percentile*  OR 130/85 mmHg (whichever is lower) | 1-13 yr: 90^th^ percentile† OR 120/80 mmHg (whichever is lower)  ≥ 13 yr: 120/80 mmHg | 0-15 yr: 90^th^ percentile*  ≥16 yr: 130/85 mmHg | 90^th^ percentile | NSR |
| Diagnostic thresholds for Stage I hypertension | 1-17 yr: 95^th^ percentile*  OR 140/90 mmHg (whichever is lower)  at 3 different visits | 1-13 yr: 95^th^ percentile†  ≥13 yr: 130/80 mmHg  at 3 different visits | 0-15 yr: 95^th^ percentile*  ≥16 yr: 140/90 mmHg  at 3 different visits | 1-5 yr: 95^th^ percentile*  6-11 yr: 95^th^ percentile* OR 120/80 mmHg  12-17 yr: 95^th^ percentile* OR 130/85 mmHg  at 3 different visits | School children: 95^th^ percentile‡  at 3 different visits |
| Diagnostic thresholds for Stage II hypertension | 1-17 yr: 95^th^ percentile* + 12 mmHg  OR 160/100 mmHg (whichever is lower) | 1-12 yr: 95^th^ percentile† + 12 mmHg OR 140/90 mmHg (whichever is lower)  ≥13 yr: 140/90 mmHg | 0-15 yr: 99^th^ percentile* + 5 mmHg  ≥16 yr: 160/100 mmHg | 95^th^ percentile* + 12 mmHg | NSR |
| Definition of a hypertensive crisis | 1-17 yr: 95^th^ percentile* + 30 mmHg  OR 180/110 mmHg (whichever is lower) | Stage II hypertension + symptoms  OR BP is >95^th^ percentile + 30 mmHg  OR BP is >180/120 mmHg | “Although there is no absolute level of BP to define severe HTN, some authors suggest to use a cutoff of 20% above the stage 2 HTN limit.” | NSR | NSR |
| Method for office-based diagnosis of hypertension | May screen with validated automated device, but confirm diagnosis with the auscultatory method | Same | Same | Same | “blood pressure measurement by auscultation is ideal” |
| Use of ABPM in diagnosis | Perform ABPM to confirm diagnosis if office BP is in Elevated for ≥1 year or Stage I over 3 clinic visits | Same | Confirm diagnosis with ABPM before starting antihypertensive therapy | “For children with elevated office BP readings, ABPM should be guided by a physician with expertise in pediatric hypertension; ABPM is useful to classify BP” | “24-h ambulatory blood pressure monitoring (ABPM) should be performed to rule out the possibility of white coat hypertension” |
| Diagnostic criteria for ambulatory hypertension | 95^th^ percentile by sex and height provided these are less than the relevant adult thresholds to define hypertension by ABPM | “Diagnosis is based on the presence of mean SBP and DBP < 95^th^ percentile and SBP and DBP load <25%” | Same | “ABPM levels should be interpreted with appropriate pediatric normative data for children 5 years of age or older or height of ≥120 cm” | NSR |
| Role of home BP | Should not be used to diagnose hypertension  May be a useful adjunct to office and ambulatory BP measurement after hypertension has been diagnosed | Should not be used to diagnose hypertension, masked hypertension, or white coat hypertension  May be a useful adjunct to office and ambulatory BP measurement after hypertension has been diagnosed | Indications: Patients receiving anti-hypertensive medication; suspicion of white coat hypertension; strict blood pressure control requirements; clinical trials | NSR | NSR |
| ABPM in children with repaired aortic coarctation | Perform ABPM to detect masked hypertension | Same | “ABPM should be performed regularly from the age of seven” | NSR | NSR |
| General examinations in patients with high BP | Perinatal history, nutritional history, physical activity history, psychosocial history, family history and perform a physical examination for signs of secondary hypertension | Same | Same | Same | NSR |
| Electrocardiography to detect left ventricular hypertrophy | Do not use | Same | Same | NSR | NSR |
| Echocardiography to assess left ventricular mass, geometry and function | Perform echocardiography when considering pharmacological treatment | Same | Perform echocardiography in all children with confirmed diagnosis of hypertension | Perform echocardiography in all children with confirmed diagnosis of hypertension | NSR |
| Doppler renal ultrasound | Should be used as a non-invasive screening study for the initial evaluation of possible renal artery stenosis in normal-weight children and adolescents ≥8 years of age. | May be used as a non-invasive screening study for the evaluation of possible RAS in normal- weight children and adolescents ≥8 y of age who are suspected of having renovascular HTN and who will cooperate with the procedure | Required in every child with hypertension | Required in every child with hypertension | NSR |
| Diagnosis of renal artery stenosis | In children and adolescents suspected of having renal artery stenosis, direct angiography is the preferred diagnostic test. | In children and adolescents suspected of having renal artery stenosis, either CTA or MRA may be performed as noninvasive imaging studies. Nuclear renography is less useful in pediatrics and should generally be avoided. | “[Can be] detected by renal Doppler ultra- sound, magnetic resonance tomography or direct renal angiography, the gold standard” | NSR | NSR |
| Testing for microalbuminuria | Only in children with diabetes mellitus | Same | Yes +/- total proteinuria quantification | Same | NSR |
| Treatment goal for non-pharmacological or pharmacologic therapy (SBP and DBP) | <90th percentile | <13 yr: <90th percentile  ≥13 yr: <130/80 mmHg | <95th percentile is recommended (140/90 for ages ≥16 yr)  <90th percentile should be considered | <95th percentile  <90th percentile for patients with risk factors, target organ damage or diabetes (Type 1 or 2) | <90^th^ percentile or <130/80 mmHg (whichever is lower) |
| Treatment goal for children with CKD | <50^th^ percentile (mean arterial BP by ABPM)  <50^th^ percentile SBP by auscultation may substitute in lieu of ABPM | <50^th^ percentile (mean arterial BP by ABPM) | <75th percentile (nonproteinuric CKD)  <50th percentile (proteinuric CKD) | NSR | NSR |
| Recommended non-pharmacological interventions | DASH diet  Moderate to vigorous physical activity at least 3 to 5 days per week (30–60 min per session)  There are additional benefits to additional exercise and avoiding sedentary time per the World Health Organisation recommendations.  Involve families in behaviour change | DASH diet  Moderate to vigorous physical activity at least 3 to 5 days per week (30–60 min per session)  Involve families in behaviour change | At least 60 min of moderate-to-vigorous-intensity physical activity daily (mostly aerobic), strength-based activity at least 3 times per week.  Avoid more than 2 hours of daily sedentary time  Avoid intake of excess sugar, excess soft drinks, saturated fat and salt and recommend fruits, vegetables and grain products Limit salt intake  Encourage smoke-free environment  Involve families in behaviour change | “A comprehensive approach should include dietary education and increased physical activity” | Reduce salt intake  Restrict energy intake, reduce overeating  Moderate to vigorous physical activity at least 3 to 5 days per week (30–60 min per session)  Involve families in behaviour change |
| First line pharmacological agents | For primary hypertension: ACEi/ARBs, long-acting or calcium channel blocker. | ACEi/ARBs, long-acting calcium channel blocker or thiazide diuretic | Dependent on clinical conditions | ACEi/ARBs or long-acting calcium channel blocker | ACEi/ARBs or long-acting calcium channel blocker |
| Criteria for pharmacological treatment | Symptomatic hypertension  Target organ damage  Stage 2 hypertension without an obvious cause (e.g. obesity)  Hypertension that persists ≥6 months despite trial of non-pharmacological therapy, employing personalised decision-making | Symptomatic hypertension  Stage 2 hypertension without an obvious cause (e.g. obesity)  Any stage of hypertension associated with CKD or diabetes mellitus | Symptomatic hypertension  Secondary hypertension  Target organ damage  Diabetes (Type 1 or 2)  Hypertension that persists for about 1 year despite trial of non-pharmacological therapy  Consider if BP is high-normal and if hypertensive TOD is already present | Symptomatic hypertension  Target organ damage  Stage 2 hypertension  BP ≥90th percentile associated with diabetes mellitus (type 1 or 2), CKD, or heart failure  Hypertension that persists ≥6 months despite trial of non-pharmacological therapy | Symptomatic hypertension  Target organ damage  Presence of CKD or diabetes mellitus  Secondary hypertension requiring drug therapy  Persistent hypertension despite non-drug therapy involving lifestyle modifications |
| CKD-specific recommendations | Evaluate for hypertension at each medical encounter  Perform regular ABPM if history of hypertension (regardless of apparent BP control)  Treat hypertension with ACE inhibitor or ARB | Same (except perform ABPM at least yearly) | Regular ABPMs at 6–12-month intervals | NSR | “renal prognosis can also be improved by maintaining blood pressure within the normal range using ACE inhibitors” |
| Diabetes-specific recommendations (Type 1 and 2) | Evaluate for hypertension at each medical encounter  Treat if BP ≥95th percentile or >140/90 mmHg | Evaluate for hypertension at each medical encounter  Treat if BP ≥95th percentile or >130/80 mmHg | Regular ABPMs at 6–12-month intervals  Treat if BP ≥95th percentile or >140/90 mmHg | NSR | NSR |
| Management of acute severe hypertension and life-threatening symptoms | Immediate treatment with short-acting antihypertensive medication  Reduce BP by no more than 25% of the planned reduction over the first 8 hours | Same | “BP should be lowered by no more than 25% of the planned BP reduction over the first 6–8h, followed by a further gradual reduction over the next 24–48h” | NSR | NSR |
| Sports participation for children/ adolescents with hypertension | The routine exclusion of children with hypertension from sports participation is not recommended.  Children with significant cardiac changes should be discussed with a cardiologist. | Children and adolescents with hypertension may participate in competitive sports once hypertensive target organ effects and cardiovascular risk have been assessed.  Children and adolescents with hypertension should receive treatment to lower BP below stage 2 thresholds before participation in competitive sports. | Competitive sports participation should be limited only in the presence of uncontrolled stage 2 hypertension | NSR | NSR |
| Transition to adult care providers | Typically by 16-18 years of age | By 22 years of age | NSR | NSR | NSR |

ABPM, Ambulatory Blood Pressure Monitoring; BP, blood pressure; CTA, Computed Tomographic Angiography; DBP, diastolic blood pressure; MRA, Magnetic Resonance Angiography; NSR, no specific recommendation for children; SBP, systolic blood pressure

‘Same’ means that the recommendation is the same as the Australian guideline

* Percentiles as per the 1999–2000 National Health and Nutrition Examination Survey (NHANES) [34]

† Percentiles from NHANES (as above) calculated after excluding individuals with overweight or obesity [4]

‡ 95th percentiles to be used for management, listed per school grade (Table 10-2) in the 2009 Japanese Society of Hypertension Guideline [35]

# 11 Criteria for Screening

**Table A9.** Criteria for screening

| **Condition** | | Criteria  Met | References |
| --- | --- | --- | --- |
|  | The condition should be an important health problem. | **✓** | 1-10 |
|  | There should be a recognisable latent or early symptomatic stage. | **✓** | 2, 3, 5-7, 36-41 |
|  | The natural history of the condition, including development from latent to declared disease, should be adequately understood. | **✓** | 3-10, 26, 28 |
| **Test** | |  |  |
|  | There should be a suitable test or examination. | **✓** | 3-10, 33, 39 |
|  | The test should be acceptable to the population. | ? | * |
| **Treatment** | |  |  |
|  | There should be an accepted treatment for patients with recognised disease. | **✓** | Guideline |
| **Screening Program** | |  |  |
|  | There should be an agreed policy on whom to treat as patients. | **✓** | Guideline |
|  | Facilities for diagnosis and treatment should be available. | **✓** |  |
|  | The cost of case-findings (including diagnosis and treatment of patients diagnosed) should be economically balanced in relation to possible expenditure on medical care as a whole. | ? |  |
|  | Case-findings should be a continuing process and not a ‘once and for all’ project. | **✓** | Guideline |

Criteria from Australian Government Department of Health. Population based screening framework. Updated August 2018. Canberra: Australian Government Department of Health; 2018. Available from: <https://www.health.gov.au/resources/publications/population-based-screening-framework>

These follow the WHO Wilson and Jungner screening criteria. Further discussed on pages 5 to 7 of the manuscript.

References listed according the main manuscript document.

* Data from the Melbourne-based Healthy Hearts @ School study (in preparation for publication) indicate >90% acceptability ratings from parents/guardians for school-based BP screening, and consistently positive feedback from students.

# 12 Pharmacological management of chronic hypertension in children and adolescents

**Table A10.** Commonly used oral antihypertensive agents for management of chronic hypertension in children and adolescents

| **Medicine** | **Class** | **Dose** | **Dosing interval** | **Formulations** | **Adverse effects‡** |
| --- | --- | --- | --- | --- | --- |
| **Calcium channel blocker** | | | | | |
| *Relative contraindication: heart failure* | | | | | |
| Amlodipine^§^ | CCB (dihydropyridine) | **Initial:** 1–5 y: 0.1 mg/kg/day (max 5 mg); ≥6 y: 2.5 mg/day **Maximum:** 0.6 mg/kg/day (max 10 mg/day) | Daily | Tablet: 2.5, 5, 10 mg Extemporaneous liquid: 1 mg/mL | Peripheral oedema (3–14%; dose related), headache (8–13%; dose-related), dizziness (1–8%), flushing (<1–10%), fatigue (<1–7%), palpitations (1–5%) |
| Isradipine^¶^ | CCB (dihydropyridine) | **Initial:** 0.05–0.1 mg/kg/dose **Maximum:** 0.6 mg/kg/day (max 10 mg/day) | Capsule: BD–TDS Tablet (ER): daily | Capsule: 2.5, 5 mg Tablet (ER): 5, 10 mg |  |
| **ACE inhibitors (ACEi) and angiotensin receptor blockers (ARBs)** | | | | | |
| *Contraindications: pregnancy, (relative – subspecialist only) bilateral renal artery stenosis, angioedema (ACEi)* | | | | | |
| *ACE inhibitors* | | | | | |
| Enalapril^§^ | ACEi | **Initial:** ≥1 mo: 0.08 mg/kg/day (max 5 mg/day) **Maximum:** 0.6 mg/kg/day (max 40 mg/day) | Daily to twice daily | Tablet: 2.5, 5, 10, 20 mg Solution: 1 mg/mL | Cough (1–5%), dizziness (2–8%), headache (2–5%), fatigue (≤6%), nausea (>1%), hyperkalaemia (0.1–6%), angioedema (0.1–0.7%), acute kidney injury (<0.1%) |
| Lisinopril^§^ | ACEi | **Initial:** ≥6 y: 0.07 mg/kg/day (up to 5 mg/day) **Maximum:** 0.6 mg/kg/day (up to 40 mg/day) | Daily | Tablet: 2.5, 5, 10, 20, 30, 40 mg Solution: 1 mg/mL |  |
| Ramipril | ACEi | **Initial:** 1.6 mg/m²/day **Maximum:** 6 mg/m²/day | Daily | Capsule: 1.25, 2.5, 5, 10 mg |  |
| *Angiotensin receptor blockers* | | | | | |
| Candesartan^§^ | ARB | **Initial:** 1–5 y: 0.02 mg/kg/day (up to 4 mg/day); ≥6 y: <50 kg: 4 mg/day, ≥50 kg: 8 mg/day **Maximum:** 1–5 y: 0.4 mg/kg/day (up to 16 mg/day); ≥6 y: <50 kg: 16 mg/day, ≥50 kg: 32 mg/day | Daily to twice daily | Tablet: 4, 8, 16, 32 mg Extemporaneous liquid: 1 mg/mL | Headache (3–11%), dizziness (2–8%), fatigue (2%), abdominal pain (1–4%), nausea (<2%), hyperkalaemia^*^, angioedema^*^, acute kidney injury^*^ |
| Olmesartan^§^ | ARB | **Initial:** ≥6 y: <35 kg: 10 mg/day; ≥35 kg: 20 mg/day **Maximum:** <35 kg: 20 mg/day; ≥35 kg: 40 mg/day | Daily | Tablet: 5, 20, 40 mg Extemporaneous liquid: 2 mg/mL |  |
| Valsartan^§^ | ARB | **Initial:** ≥1 y: 1.3 mg/kg/day (max 40 mg/day) **Maximum:** 2.7 mg/kg/day (max 160 mg/day) | Daily | Tablet: 40, 80, 160, 320 mg Extemporaneous liquid: 4 mg/mL |  |
| **Thiazide diuretics** | | | | | |
| *Relative contraindication: impaired glucose tolerance* | | | | | |
| Chlorothiazide^§¶^ | Thiazide diuretic | **Initial:** 10 mg/kg/day **Maximum:** 20 mg/kg/day (max 375 mg/day) | Daily to twice daily | Tablet: 250, 500 mg Suspension: 250 mg/5 mL *IV formulation available* | Hyperuricaemia (≥10%), hypokalaemia (1–10%), hyponatraemia (1–10%), dizziness (1–10%), headache (1–10%), nausea (1–10%), hyperglycaemia (1–10%), hypercalcaemia (1–10%), photosensitivity^†^ |
| Hydrochlorothiazide^§^ | Thiazide diuretic | **Initial:** 0.5–1 mg/kg/day **Maximum:** 2 mg/kg/day (up to 50 mg/day) | Daily to twice daily | Tablet: 12.5, 25, 50 mg |  |
| **Potassium-sparing diuretics** | | | | | |
| *Contraindication: hyperkalaemia* | | | | | |
| Amiloride | Potassium-sparing diuretic (ENaC blockade) | **Initial:** 0.4–0.6 mg/kg/day **Maximum:** 20 mg/day | Daily | Tablet: 5 mg (as Moduretic® combination in Australia) | Hyperkalaemia (∼10% monotherapy; 1–2% with thiazide), headache (3–8%), nausea (3–8%), diarrhoea (3–8%), vomiting (3–8%), dizziness (<3%), muscle cramps (<3%) |
| **Beta-adrenergic blockers** | | | | | |
| *Contraindications: asthma (relative), heart block, decompensated heart failure* | | | | | |
| Atenolol | β₁-selective blocker | **Initial:** 0.5–1 mg/kg/day **Maximum:** 2 mg/kg/day (up to 100 mg/day) | Daily to twice daily | Tablet: 25, 50, 100 mg Oral solution: 50 mg/10 mL | Fatigue (≤10%), dizziness (≤10%), bradycardia (≤10%), cold extremities, bronchospasm, GI disturbance |

**‡** All antihypertensive agents may cause hypotension. Adverse effect frequencies are derived from approved product information where available and may reflect adult data. Frequency ranges represent the range observed across individual agents within each class.

***** Post-marketing report (frequency not established from clinical trials).

**†** Thiazide frequencies from hydrochlorothiazide product information (EMA frequency categories). Chlorothiazide product information does not specify individual adverse effect frequencies.

**§** Not approved by the TGA for paediatric use in Australia. Has FDA and/or EMA paediatric indication: amlodipine (FDA ≥6 y), enalapril (FDA/EMA ≥1 mo), lisinopril (FDA/EMA ≥6 y), candesartan (FDA/EMA ≥1 y), olmesartan (FDA/EMA ≥6 y), valsartan (FDA/EMA ≥6 y), hydrochlorothiazide (FDA), chlorothiazide (FDA).

**¶** Not registered on the Australian Register of Therapeutic Goods (ARTG). No FDA or EMA paediatric indication (isradipine). Chlorothiazide has FDA paediatric indication but is not on the ARTG.

**Abbreviations:** ACEi, angiotensin-converting enzyme inhibitor; ARB, angiotensin receptor blocker; BD, twice daily; CCB, calcium channel blocker; ENaC, epithelial sodium channel; ER, extended release; GI, gastrointestinal; TDS, three times daily.

**Sources:** Dosing: Flynn JT et al. *Pediatrics* 2017;140(3):e20171904 (AAP 2017); Lurbe E et al. *J Hypertens* 2016;34:1887–1920 (ESH 2016). Adverse effects: Approved product information.

# 13 Treatment of acute severe hypertension

**Table A11.** Oral agents used for the treatment of acute severe hypertension

| **Medicine** | **Mechanism** | **Onset** | **Peak**  **effect** | **Half-life†** | **Dose** | **Adverse effects‡** |
| --- | --- | --- | --- | --- | --- | --- |
| Nifedipine | CCB | 20 min | 30-45 min | 4 h | 0.1-0.25 mg/kg/dose, may repeat dose after 1 h in acute setting (maximum daily dose 2 mg/kg) | Hypotension (5%), tachycardia (<1%), flushing (25%) |
| Isradipine | CCB | 30-60 min | 2-3 h | 6-8 h | 0.05-0.1 mg/kg/dose up to 5 mg/dose QID | Peripheral oedema (1-9%; dose related) |
| Amlodipine | CCB | 30-60 min | 6-8 h | 36 h | 0.1 mg/kg daily (max 2.5 mg), increasing to 0.4 mg/kg as required (max 10 mg) | Peripheral oedema (2-11%; dose related) |
| Clonidine | Central α-agonist | 30-60 min | 1-4 h | 6-8 h | 2-5 mcg/kg/dose (max 100 mcg), may give doses up to 1 hourly PRN in acute setting, no more than eight doses in 1 day | Sedation (2-16%), rebound hypertension and agitation on weaning |
| Hydralazine | Direct vasodilator | 20-30 min | 1-2 h | 3-7 h | 0.25 mg/kg/dose up to 25 mg/dose QID | Flushing (1-10%), headache (≥10%), dizziness (0.1-1%), tachycardia (≥10%), palpitations (≥10%), oedema (0.1-1%), blood dyscrasias (0.1- <1%) |
| Prazosin | Alpha adrenergic blocker | 30-90 min | 2 h | 2-4 h | 0.05-0.1 mg/kg/day in three divided doses, increasing to 0.5 mg/kg/day as required | Dizziness (10%), headache (8%), orthostatic hypotension (1-4%), lethargy and palpitations (5%) |
| Captopril | ACEi | 20-30 min | 1 h | 2-3 h | 0.3 to 0.5 mg/kg/dose (max 25 mg) TDS, increasing to 6 mg/kg/day as required (max 150 mg per dose) | Hyperkalaemia, acute kidney injury (<1%) |
| Minoxidil | Direct vasodilator | 5-30 min | 2-3 h | 3-4 h | 0.1-0.2 mg/kg/dose BD-TDS, increasing to 50 mg/day in children <12 years to 100 mg/day in older children. | Hypertrichosis (80%), pericardial effusion (3%) |
| Labetalol | α- and β-blocker | 20 min to 2 h | 2-4 h | 6-8 h | 1-3 mg/kg/day, increasing to 12 mg/kg/day in two divided doses (max 1200 mg/day) | Dizziness (20%), oedema (2%), bronchospasm (<1%), bradycardia (<1%) |
| Propranolol | β-blocker | 1-2 h | 1-4 h | 3.5-6.4 h | 1-2 mg/kg/day, increasing to 4 mg/kg/day in two or three divided doses (max 640 mg/day) | Sleep disorder (infants: 16-18%), bronchitis (infants: 8-13%), drowsiness (infants: 5%) and decreased appetite (infants: 3-4%) |

† The relevance of this is to the time to reach steady state with continued use. The progression to steady state is not a linear one. However, it takes three half-lives to reach 90% of the final serum concentration. ‡ All peripheral vasodilators without substantial chronotropic or inotropic effects may cause tachycardia, hypotension and fluid retention. Some medications, such as calcium channel blockers and hydralazine, are known to produce idiosyncratic reductions in BP. Frequency of adverse effects: rare (<0.1%), infrequent (0.1-1%) and common (>1%). ACEi, angiotensin-converting enzyme inhibitor; CCB, calcium channel blocker. Reproduced from Hijazzi S, Moon K, Larkins NG. Oral agents for acute severe hypertension in children with minimal or no symptoms, J Peditr Child Health, 2022; 58(11):1935-1941, with permission from John Wiley & Sons.

# 15 Reviewers and organisations involved the consultation process

| **Reviewing Organisations** | |
| --- | --- |
| National Heart Foundation of Australia | |
| Royal Australasian College of Physicians (RACP) | |
| Kidney Health Australia  Royal Australian College of General Practitioners (RACGP)  Cardiac Society of Australian and New Zealand (CSANZ)  Australian New Zealand Paediatric Obesity Network (ANZPON)  Australia and New Zealand Paediatric Nephrology Association (ANZPNA) | |
| **Individual Reviewers** | |
| Prof Jeanie Cheong | Neonatologist, MCRI and Royal Women’s Hospital, Melbourne. |
| Fiona Mackie | Consultant Paediatric Nephrologist, Sydney Children’s Hospital, Sydney. |
| Nephrology Department | Women and Children’s Hospital, Adelaide |
| Prof Shirley Alexander | Paediatrician, Medical co-chair/Dep. Head Westmead, University of Technology Sydney, NSW |
| Prof Yvonne Anderson | Associate Dean, Head of Medicine and Professor of Community Child Health, Curtin University, Perth, WA |
| A/Prof Ralph G Audehm | General Practitioner, Honorary Clinical Associate Professor, Department of General Practice, University of Melbourne, VI |
| Dr Simon A Carter | Consultant Paediatric Nephrologist, Royal Children’s Hospital, Melbourne, VIC. |
| Dr Melanie Clarke | Exercise Physiologist, Southern Cross University, Coffs Harbour, QLD |
| A/Prof Anna Francis | Paediatric Nephrologist, Queensland Children’s Hospital, Brisbane, QLD |
| Prof Joseph Flynn | Professor of Pediatrics, University of Washington, Seattle, Washington, USA |
| Dr Leah Krischock | Consultant Paediatric Nephrologist, Sydney Children’s Hospital, Sydney, NSW |
| Dr Joshua Kausman | Consultant Paediatric Nephrologist, Royal Children’s Hospital, Melbourne, VIC |
| Dr Beatrice Leuthold | Paediatrician, Growing Healthy Kids, NSW |
| Dr Natalie Lister | Chair of ANZPON, Senior Research Fellow (Dietitian), University of Sydney, NSW |
| Dr Payal Mandaliya | Staff Specialist Paediatric Respiratory and General Paediatrics, Maitland Hospital, Conjoint Lecturer, University of Newcastle, Newcastle, NSW |
| Dr Andrew Martin | General Paediatrician, Perth Children's Hospital, Perth, WA |

# 16 Responses to comments from consultation

The committee would like to warmly thank all the reviewers of the guideline.

Comments and responses have been organised according to sections of the guideline. Comments from all reviewers have been collated and de-identified. Minor comments (regarding typographical or wording) have been actioned but are not included below. Where parts of the guideline have been quoted, references are removed (see the guideline manuscript for references).

## 16.1 Identification of hypertension: screening in children and adolescents; Recommendations 1-2; Table 3: Conditions associated with an increased risk of hypertension

Comment: The two-point screening strategy (approx. 7–9 y and 13–15 y) is pragmatic and better aligned with current Australian general-practice workflows than the annual approach in the AAP guideline. The accompanying narrative clearly explains why annual screening is infeasible under the current Medicare Benefits Schedule and school-health infrastructure, and why childhood BP tracks into adult cardiovascular risk. Suggestion: Authors to specify how opportunistic screening (e.g. during immunisation visits) should be documented to ensure follow-up of abnormal values.

*Response: We have expanded following feedback from stakeholders:*

*‘Currently, the healthcare professionals most visited by children are GPs, and those performing routine vaccinations. While both settings are not optimal in ascertaining an accurate BP in children due to the associated stress at the time of presentation, the former is more practical in obtaining a settled measurement and follow-up of elevated results. Hence, recommendations need to be implementable in the context of current general practice workflows, time demands, and billing constraints in the Australian system. There are good arguments for community-based screening. Further investment into the resources needed to document and act on abnormal values would be required to enact this in practice. Collaboration across the spectrum of cardiovascular and health promotion is required to continue developing opportunities for proactive identification and intervention for at-risk children. It is also important that screening recommendations do not detract from broader public health measures to address cardiovascular risk factors across the life-course.’*

Comment: This recommendation contradicts the guidance in the RACGP’s Guidelines for preventive activities in general practice (Red book), which states ‘Screening for high blood pressure (BP) in children and adolescents is generally not recommended’. Asymptomatic screening of a whole population needs to meet the definitions for a universal screening program to be warranted for use for the entire population. The adoption of the AAP guideline is not sufficient to support such an approach in the Australian context—particularly for blood pressure measurement, where factors like cuff size, patient positioning, and emotional state can significantly influence results.

*Response: We note the RACGP Red Book guidance that does not recommend universal BP screening in asymptomatic children and appreciate the importance of addressing this discrepancy. During development, a dedicated workshop facilitated by clinical epidemiologists evaluated the evidence for screening against the WHO Wilson and Jungner criteria. The results of this evaluation are summarised in the supplementary material (Table A9 in this Supplement).*

*We agree that BP measurement in very young children can be challenging. However, evidence demonstrates that from age 7–9 years, within-person visit-to-visit variability and BP tracking over time is equivalent to that observed in adults, supporting the feasibility of accurate measurement from this age [36-39]. This was the rationale for selecting 7–9 years as the lower age limit for screening. Regarding equipment, many healthcare providers would consider BP a vital sign that GPs seeing unwell children should be equipped to measure. Appropriate cuffs for existing machines are relatively inexpensive to source; however, we acknowledge that the equipment for manual measurement of BP is less commonly available and thus advised that oscillometric BP measurement is appropriate for initial measurements. We also note that 20,000 Omron HEM 907 oscillometric devices were distributed for free by the High Blood Pressure Research Council to general practitioners, which are still in wide use, and were provided with a paediatric cuff.*

Comment: The recommendation to screen is primarily based on the 2017 AAP guideline, which cites Level C evidence (defined as low-quality or conflicting observational studies). The descriptive text indicates the USPSTF evidence review was discounted in the AAP’s screening recommendations. Before implementing a major shift in practice, a thorough review of the evidence regarding both harms and benefits is essential. Additionally, modelling data showing the number needed to screen to prevent a serious outcome that would otherwise go undetected should be clearly presented.

*Response: The consensus reached by the guideline Clinical Advisory Panel to recommend screening children for hypertension is based on recent evidence provided by the International Childhood Cardiovascular Cohort (i3C) consortium which was published in NEJM in 2022, and has been expanded upon in more recent analyses to determine direct and indirect risks of childhood hypertension [40, 41]. This cohort study involving 38,589 children across 7 countries, including Australia, demonstrated that childhood BP predicts fatal and non-fatal cardiovascular events in mid-adulthood (mean age 47.8 years, SD 8 years), independent of BMI and lipid status. Similar to adult populations, a systolic blood pressure at the 90th percentile was associated with a doubling of the hazard for future clinical events [42]. Moreover, mediation analysis from this dataset revealed that almost 20% of the risk conferred by high blood pressure on cardiovascular disease is ‘laid down’ in childhood and adolescence (i.e. not mediated by the adult risk factor), with the remainder of the attributable risk due to BP tracking [41]. The i3C data demonstrating direct associations with fatal and non-fatal cardiovascular events has since been externally validated by two additional studies [43, 44]. These data were not available at the time of the USPSTF recommendation and fill one of the major evidence gaps raised by the taskforce.*

*We appreciate that the evidence for screening childhood BP does not fully meet the same robust criteria as other national screening programs such as that for cervical, breast and colon cancer screening. We acknowledge the request for modelling data on the number needed to screen. However, consistent with all international guideline groups (Europe, Canada, Korea, Japan and Brazil), we concluded that the multi-decade randomised interventional trials needed to quantify treatment benefit to inform accurate modelling will never be feasible or ethical. Similarly, it is not anticipated that randomised data evaluating a screening program, as is possible among adult populations, will be available within a reasonable timeframe. Given these practical limitations, we have recommended screening based on the (now very strong) longitudinal data demonstrating the impact of childhood BP on cardiovascular events in adult life, the increasing prevalence and earlier onset of hypertension and related clinical events, and need for a whole of life approach to cardiovascular risk. The best available data has been used to inform the recommendation, and the evidence-base is now at a point where additional data, short of a large randomised controlled trial, is unlikely to result in substantial changes to the synthesis in the short- to medium-term.*

Comment: There should be a differentiation in the guidelines between universal screening, which has a weaker evidence base, and targeted screening of high-risk groups, which is far more likely to identify children who would benefit from early intervention.

*Response: We acknowledge the suggestion to differentiate universal from targeted screening. At present, we are not aware of evidence demonstrating benefit for targeted screening compared to universal approaches. Although there is no question that high blood pressure is more common in children with obesity, we note that the association of childhood BP with later fatal and non-fatal cardiovascular events is independent of BMI. Moreover, whilst targeted screening in practice is most likely to focus on body weight, this approach would disadvantage children with high blood pressure related to prematurity, genetic predisposition, or a range of other conditions.*

Comment: The definition for initial screening is too generous particularly for younger children.

*Response: This was debated by the panel, that included general practitioners and paediatricians from health services where EMR are not available. Aside from being linked to left ventricular hypertrophy (initially proposed based on prediction of in the Bogalusa cohort), the thresholds given are at the 95^th^ percentile for a child on the 50^th^ percentile of height at the lower end of the age range (i.e. for most children in the range they are lower than the 95^th^ percentile). We agree there is a risk of implementation for shorter children and/or practitioners inadvertently missing that these are intended for low-risk populations. A flowchart has been added, which includes specifically pointing this out. We have also developed an online calculator for BP values that classifies BP for a given patient and provides the relevant person-specific 95^th^ percentile.*

Comment: Should be able to implement age/gender based values into primary practice software, EMR or easy link to online calculator.

*Response: Yes, we plan to work with EMR and primary practice software providers to facilitate implementation.*

Comment: The simplified thresholds for primary care screening make a lot of sense and I am supportive of this pragmatic approach. Reinforcement of the following caveats should be considered around the need for percentile based assessment for: 1. Children with comorbidities that raise the risk of HT. 2. Very small children eg Height or BMI <5th percentile.

*Response: We have addressed these points at the top of a clinical flow diagram, which will aid providers in recognising these scenarios.*

Comment: Suggest considering starting this section with a brief comment re clinical recognition of symptomatic/urgent hypertension in children, then follow that with the section on screening. From a clinical point of view it is vital that urgent situations are recognised.

*Response: We have added a flowchart to guide use of the thresholds in practice. This includes specific advice about the urgency of follow-up according to symptoms and BP.*

Comment: Suggest considering if there are any potential caveats re taking the screening approach to blood pressure in kids, for example will screening/detection approach take away, or detract from population public health measures to lower blood pressure in ALL kids? Do population public health measures remain important?

*Response: We agree that public health measures are critical. We have expanded the discussion about the logistics of implementation per the earlier suggestion.*

Comment: Re Table 1 ABPM - how does the current MBS item work for kids? Are the BP threshold for eligibility suitable for children, or do they mean that some kids with BP's of concern may not be eligible for a MBS supported ABPM?

*Response: Currently all ABPM are performed via tertiary hospitals, almost exclusively paediatric nephrology services. We agree this is a major concern and have made submission to MSAC to consider amending the relevant item to facilitate equitable access for children (pending budget review).*

Comment: Table 1 is good, table 2 of evidence is very long - really need a summary guide

*Response: We agree that Table 2 is not an easy read. Table 1 is intended as the summary guide. We have added also a flowchart highlighting possible patient flows resulting from a high BP reading. This includes references to relevant sections and we hope makes the guideline easier to navigate in practice. Table 2 is needed to highlight the specific recommendations that were adopted/adapted/rejected from the Key Action Statements of the 2017 AAP Guideline under the ADAPTE framework.*

Comment: It is recommended that blood pressure (BP) monitoring for patients on stimulant medications be conducted every six months, rather than at every health encounter. Given that stimulant prescriptions are issued for a maximum duration of six months, patients are required to see their paediatrician or GP (in shared care arrangements) at least twice a year. Aligning BP checks with these visits is a more practical and sustainable approach. As noted on page 6 of the document: "Recommendations need to be implementable in the context of current general practice workflows, time demands, and billing constraints in the Australian system." The same principle applies to monitoring children prescribed stimulants. A recent Cochrane review (Chan et al., 2025), which included 56 RCTs, reported an average increase in BP of 2 mmHg and heart rate of 4 bpm in both children and adults on stimulant medications. While the review considered this statistically significant, the clinical impact-particularly in children without underlying cardiac or other risk factors-is minimal. Moreover, minor variations such as a 2 mmHg difference may result from factors like machine variability, operator technique, or time of day, further supporting a more measured approach to BP monitoring. Ref: Chan M, Chan JJ, Wright JM. Effect of amphetamines on blood pressure. Cochrane Database Syst Rev. 2025 Mar 28;3(3):CD007896. doi: 10.1002/14651858.CD007896.pub4. PMID: 40152309; PMCID: PMC11951410 Agree with BP check with every health counter in children or adolescents who are on systemic steroids

*Response: The recommendation to check BP with every care encounter is intended to reflect routine practice. For children on stimulants that would mean every six months. We believe this reflects current practice and should not increase care requirements. We agree that the average increase in BP with stimulant medications is modest and it is important not to overplay the risk. However, not infrequently stimulant medications are enough to push a child with concurrent risk factors into the hypertensive range and there is variation about the mean effect. It is also difficult to advise against in the context of the Australian PI for commonly used stimulants such as dexamphetamine and lisdexamphetamine, which advise regular monitoring of BP in all patients.*

Comment: Consider further discussion on the role of epigenetics with increased risk of hypertension in children. i.e., maternal and paternal lifestyle factors contributing to increased risk of hypertension in child pre development by altering expression in genes related to the renin-angiotensin-aldosterone system, endothelial function, and inflammation. While touched upon in the Table 3, it may demonstrate forward thinking compared with other recommendations/guidelines and considerations of the family unit pre-conception.

*Response: We agree that this would be an interesting discussion, but we have avoided from expanding upon the pathophysiology of hypertension for this guideline. It will be accompanied by commentary pieces, including for general paediatricians, in which we can elucidate this further as it is an important, forward looking consideration that reflects what we know already supporting a life-course approach to BP and CV risk.*

Comment: “The latter has been debated widely and clinical groups believe that the certainty of evidence required by the USPTF was not realistic in the context of childhood hypertension.” Perhaps "level" or "type" might be better words than “certainty”. The commentary you cite was for the 2013 USPSTF report. See also Flynn, JAMA Network Open 2020;3:e2027964 for comments on the more recent report.

*Response: We agree. Amended. Reference added.*

Comment: “… presence of target organ damage, and concurrent risk factors impacting the trajectory towards downstream disease”. “Downstream disease” might be confusing to the general reader - it was for me.

*Response: Simplified this to ‘clinical events’.*

Comment: “The developmental stage of adolescence guided the second screening point of 13 to 15 years old …” Although probably published after your evidence review was completed, I would cite this recent paper as it provides additional support for your approach: Meng Y, Sharman JE, Iiskala F, Wu F, Juonala M, Pahkala K, Rovio SP, Fraser BJ, Kelly RK, Hutri N, Kähönen M, Laitinen T, Jula A, Viikari JSA, Raitakari OT, Magnussen CG. Tracking and Transition Probability of Blood Pressure From Childhood to Midadulthood. JAMA Pediatr. 2025 Jan 1;179(1):34-45. PMID: 39495520;

*Response: Noted and added.*

Comment: It is noted that BP screening for all children is recommended twice during childhood in those without other risk factors, at approximately 7 to 9 and 13 to 15 years of age. We can see as per table A1 and comments in section 3.3.4 the recommendation is primary care or school-based implementation is the preferred option of the taskforce. We would like to note that for this to be implemented it will require a major upskilling / awareness of the primary care workforce that would require resources and funding allocation which may need further feasibility investigation.

*Response: We agree that implementation will be a major challenge and that these guidelines would represent a major shift toward acknowledging our ability to identify people at risk of future cardiovascular and kidney disease in childhood and adolescence. We are working with GPs, dieticians, and other healthcare providers to develop a package of resources to be published with or soon after this guideline. We do expect that it will take some time for the guidelines to be widely implemented. However, in the initial stage, we think it is important to outline what the interdisciplinary group clinicians, researchers and consumers involved in this guideline see as best practice. It is then our responsibility to continue working with stakeholders and the community to take a proactive approach towards making this possible, acknowledging that the relevant process will take several years to build.*

## 16.2 Defining hypertension; Recommendations 3-4; Table 4: Thresholds for the diagnosis of hypertension

Comment: The choice of ≥ 95th percentile or ≥ 140/90 mmHg (whichever is lower) for diagnosis maintains continuity with adult definitions and the 2016 Heart Foundation adult guideline. However, please note the Heart Foundation is currently updating the hypertension guideline and this definition of hypertension may change in 2026 following review of the literature and consensus from the expert steering group. The simplified screening cut-offs (120/80 and 130/85 mmHg) derived from Hypertension Canada 2020 are well justified as an implementation aid.

- Suggest to clarify in Table 4 that percentile references are the 2016/2023 ESH data sets and provide a web link to the proposed Australian calculator.

*Response: A link will be active by the time the guideline is released. Placeholder in text added.*

Comment. Recommendation 4 (EHR flagging) is sensible but the strength of recommendation is graded ‘weak’; elaborate on potential unintended consequences (alert overload) and propose a standardised template for paediatric BP entry to mitigate this.

*Response: We agree that alert overload is a concern and have added discussion about the importance of supporting implementation.*

*‘The recommendation to include BP alerts for children in digital medical record systems was adopted, noting the need for careful implementation and benefit of working with providers (e.g. development of standardised templates).’*

Comment: Concern that immediate action not required for moderate hypertension and for severe only

*Response: Stage 2 hypertension is relatively common and without symptoms is best managed in a sequential fashion, with avoidance of overly aggressive BP reduction. Moderate hypertension is analogous to a BP of 160/100 mmHg in the Heart Foundation Guidelines and would not typically warrant immediate action in that population. Children are no different in this regard.*

Comment: Page 7, suggest providing further information re the rationale for the changed US definition of hypertension for adults, which is where the risk of CV disease is elevated 2 fold, rather than where there is a well-established threshold for treatment. I do not think there is a substantial body of evidence that initiating BP lowering therapy at this level in lower risk groups has an effect on CV outcome events. As suggested previously shift, and slightly expand on the section regarding symptomatic hypertension requiring urgent review, and put it at the beginning of this section. Although this is an uncommon problem, it is essential that it is clinically recognised as an urgent condition requiring immediate attention.

*Response: We have avoided delving too deeply into the US adult definition of hypertension, as we agree the discussion is nuanced once and based on events in adult populations. Our initial efforts as a combined paediatric collaborative are to maintain consistency with the Australian adult definition. A flowchart has been added to aid in distinguishing the urgency of action for different stages of hypertension and recognition of acute severe hypertension.*

Comment: The simplified approach is welcomed with the follow up of ABPM being standard - accessing appropriate ABPM may be more problematic. Highlight this in detail.

*Response: We have made a submission to MSAC to improve equity of access to ABPM for children. A decision from the related budget review process is expected in mid-2026. Added to the manuscript:*

*‘ABPM for children and adolescents is currently accessed via paediatric nephrology units operating in tertiary hospitals. Broadening access represents an important component of guideline implementation and efforts have begun to advocate for expanded out of hospital access.’*

Comment: Please clarify if all three measurements of either systolic or diastolic need to be breached x3 to be diagnostic. i.e. either systolic >thresholdx3, and/or diastolic >threshold x3 measurements. Also if the initial reading was oscillometric clarify that 3x subsequent auscultatory readings need to be raised (not including the initial raised oscillometric reading). Perhaps explicitly state what should be done to diagnose hypertension in an inpatient as compared with the outpatient setting?

*Response: We agree that this could be clearer. Flowchart added to aid use of the guidelines and with specific recommendations regarding the timing of repeat measurements + more urgent action for stage II hypertension or acute severe hypertension.*

Comment: “European and American guidelines were aligned, until overweight and obese participants were removed from the reference data for the AAP Guideline producing threshold values that were more contiguous with an updated US definition of hypertension among adults of 130/80 mmHg.” No, this is not why we did this. We did this because right after the Fourth Report was published, Paul Muntner published a paper in JAMA showing that obesity was driving higher BP values in US children. We realized that these higher BP values were unhealthy. This led to Rosner's 2008 paper in the AM J Epi (which you need to cite) showing how obesity affected the Fourth Report normative data. So, when we did the AAP guideline we knew we had the opportunity to correct this mistake and publish normative data reflecting healthy weight children only.

*Response: Thank you for this insight. We made changes to the relevant section.*

*‘European and American guidelines were aligned, until participants with overweight and obesity were removed from the reference data for the AAP Guideline. This allowed for the inclusion of more recent data from populations with an increased prevalence of overweight and obesity, but from an epidemiology perspective the reference values are no longer based on a normal distribution which should include 15% of participants with overweight or obesity. One advantage of the new threshold values was that they were more contiguous with an updated US definition of hypertension among adults of 130/80 mmHg.[45-48] Whilst subsequent studies have shown that the AAP Guideline thresholds have an increased sensitivity to detect target organ damage in children,[49] this is expected because of the continuous relationship between risk and BP into what is typically considered the normal range.[40] Given this relationship, if thresholds are based on the lowest value associated with a statistically significant increase in risk, then the main determinant of thresholds will be sample size of the included population. Hence, in-lieu of evidence for clinically meaningful improvements in overall diagnostic test accuracy at lower thresholds the panel recommends using the ESH reference for BP percentiles (equivalent to previous United States Fourth Report Guideline). As importantly, these are more consistent with the current definition of hypertension in Australian adults of 140/90 mmHg at 18 years of age.[48, 50-52] Further research is in progress, including data from the SHIP-AHOY study and i3c that may lead to this recommendation being updated. The principle of maintaining consistency with guidelines for Australian adults would also require the paediatric definition to be revisited should the threshold for defining hypertension in the adult population change in the future.’*

Comment: “Without evidence of clinically meaningful improvements in overall diagnostic test accuracy at lower thresholds the panel recommends using the ESH reference for BP percentiles (equivalent to previous United States Fourth Report Guideline), given these are more consistent with the current definition of hypertension in Australian adults of 140/90 mmHg at 18 years of age.” I understand that you need to align with the Australian adult GL - this is why the ESH and ESC thresholds are different from those in the AAP guideline. I hope we can get away from this approach once the pediatric community at large has absorbed and embraced the data from SHIP-AHOY.

*Response:* We have added reference to the SHIP-AHOY study. We agree there is work to do here. but we see light on the horizon in international paediatric hypertension collaboration. Concurrent projects examining the impact of BP thresholds on future cardiovascular events are also likely to be informative.

Comment: Table 4. Fail to see the purpose of including a stage 3 hypertension category - evaluation and management will differ little from stage 2 hypertension.

*Response: This to align with the Heart Foundation wording. The difference is that stage III hypertension would usually warrant admission for an urgent work-up, whereas not all patients with stage II hypertension require this. Symptoms remain important. Flowchart added to clarify.*

## 16.3 Measurement methods; Recommendation 5; Table 5: Measurement of blood pressure in children

Comment: The manuscript accurately reflects the evidence favouring auscultation for diagnostic confirmation and stresses cuff selection and device validation. Table 5 is clear and implementable.

- Add an explicit statement that wrist-based and cuffless devices are not recommended (this appears in text but not in Table 5).

- Provide an appendix linking to the STRIDE-BP paediatric-validated device list to facilitate procurement.

*Response: Added to Table 5:*

‘Wrist and cuffless blood pressure devices are not currently recommended.’

*We have provided reference to the STRIDE-BP website in the manuscript because this is the most current and complete repository of devices. A published list risks being out of date at the time of usage.*

Comment: Suggest that in addition to the information re the cuff and the device, that some comment is made re the general set up of the PERSON ie BP usually taken in seated position, in a chair with back and arm supported, feet flat on floor, legs not crossed. My understanding is that it is important that the back is supported, so I am not sure if using a stool to sit on is adequate. Table 5 - see above, I am not sure a stool is ok. It wouldn't be for adults.

*Response: We agree that sitting on a stool is unlikely to be equivalent. Clarified as follows:*

*‘The child should be in the seated position, with the back supported and feet resting flat on the floor. ’*

Comment: It may be worthwhile discerning whether instructions such as cuffs sizing and arm at heart level are instructions regardless of measurement method- auscultatory vs oscillometric.

*Response: Clarification added to Table 5.*

*‘Advice regarding cuff size and positioning, and patient preparation, applies to both oscillometric and auscultatory methods.’*

Comment: Table 5: For infants, perhaps specific ideally not sucking or feeding during measurement

*Response: Added: ‘Ideally, infants should not be sucking / feeding at the time of measurement.’*

Comment: Might you mention that oscillometric devices do not actually measure BP? I think there is a great deal of misunderstanding about how they work and what the numbers they spit out actually are.

*Response: Added to discussion about the use of oscillometric devices and their limitations:*

*‘They measure the mean arterial pressure by proxy of the amplitude of oscillations in pulse pressure, and then use proprietary equations to estimate systolic and diastolic values.’*

Comment: Also include validatebp.org

*This includes only one paediatric monitor at present and might give a false impression of availability.*

## 16.4 Out of office BP measurement; Recommendations 6-9; Table 6: Maximal out-of-office equivalents for thresholds defining hypertension

Comment: The endorsement of ABPM as the diagnostic reference standard is strongly evidence-based. Table 6 re-uses adult ABPM thresholds, which are appropriate for ≥ 18 y but may over- or underestimate risk in younger adolescents.

*Response: We have amended the title of the relevant table for clarity, which we now realise was somewhat confusing. The table has been updated and is now titled:*

***‘Table 6*** *Out-of-office thresholds defining hypertension’*

- Recommendation 9 indicates HBP is not suitable for diagnosis; emphasise that HBP may nevertheless be valuable for ongoing monitoring when ABPM is unavailable, with education on proper technique.

*Response: In principle we agree and have taken a more pro-active approach to HBP than other guidelines for children. However, there are a scarcity of data to support the accuracy of HBP in children despite the panel and consumer representatives acknowledging they find them useful in clinical practice. A handout based on the Hypertension Australia patient information for adults will be released at publication, which includes education on technique.*

Comment: The simplification of ABPM interpretation makes sense, however the statement “...new data that failed to demonstrate benefit by the inclusion of BP load to predict LVH in children.35,59” is somewhat misleading. Ref 35 only refers to adolescents >13yrs, while ref 59 only refers to children with CKD.

*Response: This is the best available data currently. In-lieu of evidence to support the inclusion of load we believe that the simplification proposed is reasonable and in-line with the updated US guidelines, which effectively serve as the global reference for 24-hour BP monitoring in children.*

Comment: Can you briefly describe the difference between ABPM and HBP for the non-specialist? I had to look up as I wasn't sure, as not tests I currently request.

*Response: Thank you for the insight. We have added the following to the beginning of the ABPM section:*

*‘Ambulatory blood pressure monitoring (ABPM) involves measuring BP at regular intervals over ≥24 hours using approved devices and standardised methods to obtain information about out of office BP, providing further data on BP variation including during sleep.’*

Comment: Re ABPM levels being higher than clinic in children, is this for both day and night time readings, or only for the daytime?

*Response: Thank you for the question. This only applies during the daytime, night values are lower. Clarified:*

*‘It is useful to note that, unlike adults, mean daytime ABPM values for children are higher than clinic or home blood pressure (HBP) values, which is reflected in the daytime ABPM percentiles.[53-55]’*

Comment: In the title for Table 6 please qualify this is for adults

*Response: The content and title of this table have been updated for clarity.*

Comment: Highlight this in detail: (are you planning a brief guide for general practice) HBP should be performed for 7 days (minimum 5) in a manner similar to that for optimal clinical BP; resting, feet flat on the floor, with an appropriately sized cuff and validated machine. Further resources including a HBP patient diary and advice for accurate measurement are available via the Hypertension Australia website (https://www.hypertension.org.au/health-professionals/#professionals-resources).

*Response: We have a draft template and advice for families prepared using the existing Hypertension Australia materials as a template. This highlights the importance of methods and will be accessible on publication.*

Comment: Consider explicitly stating whether an arm resting on a table is acceptable for any form of measurement- for example- at home measurements vs 'in office' measurements. If it is not acceptable, it may be worth denoting. Especially if medical and healthcare practitioners are relying on these for appropriate BP measurement methods.

*Response: We have added the following to table 5 ‘The arm should be supported on a desk or table, with the cuff positioned at heart level’. Reference to Professor Brady’s randomised crossover trial of arm positioning when measuring BP in children has been added to the manuscript text - ‘Arm positioning is important, with randomised crossover trial data confirming that arm positioning by the side or lap increases BP potentially resulting in over-diagnosis of hypertension.[56]’*

Comment: For home BP, some limitations should be mentioned, including the scarcity of devices validated in children, and the lack of normative data outside of 600 children from Greece.

*Response: We agree that there are limitations to HBP, but also had feedback from other stakeholders, consumers and clinicians that they find it a useful and part of routine clinical practice. There are similar limitations to normative data for ABPM in children. The manuscript includes the following discussion when introducing HBP:*

*‘Relatively few studies have been performed in children regarding the use of HBP monitoring on the diagnosis of hypertension when compared to ABPM.[57] These have generally reported poor sensitivity (52% to 55%) of HBP for the diagnosis of hypertension and masked hypertension, but better sensitivity and specificity (89% and 92%, respectively) for the detection of white coat hypertension when compared to ABPM.[58, 59]’*

## 16.5 General investigation of hypertension; Recommendations 10-12; Table 7: Sequential evaluation of childhood hypertension

Comment: The tiered approach in Table 7 is comprehensive. The decision to forego extensive secondary work-up in adolescents with obesity or familial primary hypertension is sensible and supported by recent cohort data.

-Re-order Table 7 to separate “tests for all” from “tests based on clinical suspicion” to avoid overuse of specialist investigations.

*Response: Table 7 begins with tests for ‘all children’, through to ‘Consider based on history and examination, or any child with severe or difficult to control hypertension’.*

Comment: Plasma renin activity and serum aldosterone – would be better as Direct renin concentration and plasma aldosterone. (Plasma renin activity is only measured by one lab in Australia; most others measure renin concentration)

*Response: Amended.*

Comment: -Please look at the papers referenced in the attached documents.

-In addition, there is no mention of screening for endocrine causes of hypertension, including adrenal aetiologies, which – although uncommon – remain important differential diagnoses, particularly in younger populations.

The following reference would be used to cite: Pellegrini B, Bonaventura I, Hasenmajer V, Simeoli C, Pivonello C, Ferrari D, Criscuolo S, Tomaselli A, Isidori AM, Grossman AB, Lenzi A, De Martino MC, Savage MO. Adrenal causes of endocrine hypertension in childhood or adolescence. J Endocrinol Invest. 2025 Jun 20. doi: 10.1007/s40618-025-02633-1. Epub ahead of print. PMID: 40540150.

-It would be helpful to briefly acknowledge the relevance of endocrine causes, such as adrenal hypertension, within the diagnostic framework.

*Response: Endocrine causes are listed in the table of recommended investigations, including TFT/cortisol/metanephrines/renin and aldosterone. The review cited discusses monogenic causes of hypertension, which we agree should be screened for in selected cases. We have added the following section to clarify the role of renin and aldosterone levels with regards to adrenal causes:*

*‘The AAP 2017 and ESH 2016/2023 Guidelines recommended the removal of routine renin and aldosterone testing for children with hypertension at low-risk of secondary causes. These tests have a low yield in even among children presenting to tertiary hypertension clinics and poorly predict undetected renovascular disease.[60-62] Primary aldosteronism is an area of active research; however, data indicate continuum of aldosterone dysregulation and diagnostic thresholds vary widely.[63, 64] Renin and aldosteronism remain part of screening for young children or those with severe hypertension. Low renin values should prompt investigation for monogenic disorders of hypertension, all of which are associated with low renin aside from autosomal dominant hypertension with brachydactyly.[65]’*

Comment: Renal US should be for all patients, dopplers only if indicated.

Comment: I am not sure of the meaning of the middle paragraph on page 12 - does this mean this guideline recommends or does not recommend kidney ultrasound? Suggest review for increased clarity.

*Response: This issue was debated by the panel and there was a diversity of opinions, evidenced by other feedback on the guideline. The problem is that most studies are from tertiary centres with a selection bias towards secondary hypertension. The best evidence is thought to be from an unselected population included in trials of anti-hypertensive agents (Flynn et al 2012), which is the basis for the 2017 AAP recommendation to restrict screening to children <6 years old.* However, in practice many clinicians ordered routine ultrasound and thus we have attempted to present a balanced recommendation that is not overly prescriptive to allow for different approaches within the range of accepted clinical practice:

*‘If not routinely, then in young children (<6 years) and those being considered for pharmacotherapy.’*

*The discussion has been updated to expand on this section:*

*‘This pathway aligns with the AAP 2017 Guideline, aside from the recommendation for routine kidney ultrasound. Accurate data on the diagnostic yield of screening ultrasound is hard to obtain, with studies including children referred to specialist hypertension clinics indicating that contributory ultrasound findings are common among children without other abnormalities, and that approximately half of children with chronic kidney disease (CKD) do not have proteinuria.[61, 66] Yet, among 321 children enrolled in trials of valsartan, renal tract ultrasound abnormalities were uncommon among those ≥6 years of age.[67]’*

Comment: Opthalmology review (ECHO is there to assess for end organ damage and table 8 mentions LVH and HT retinopathy, but no assessment of retina?)

*Response: This is no longer recommended as standard practice. The following has been added to clarify:*

*‘Routine ophthalmology review is not recommended as part of the initial evaluation of children with stage I or early-stage II hypertension, hypertensive retinopathy being rare in children without severe hypertension and/or an identified secondary cause.[68]’*

Comment: General comment re terminology - in some places in the document the term 'essential' hypertension is used, in other places it is 'primary' hypertension. Suggest review document for consistency of terminology.

*Response: The term essential hypertension is used throughout the manuscript aside from Table 2, when primary hypertension is kept for consistency with the adopted AAP Key Action Statements.*

Comment: Should lipids be fasting? Whilst agree aHUS, TMA, polycythaemia etc are rare causes of hypertension I wonder if pragmatically we should include FBE in the initial testing tier? I strongly suspect >90% of us and GPs have already long ordered this test before we hit tier 3.

*Response: Non-fasting lipids are appropriate for children, as for adults. Table 7 amended for clarity. While an FBE (analogous to FBP or CBC) may be commonly ordered, we cannot find any evidence to support its inclusion as a universal test in the primary care setting.*

Comment: I think the recommendation to do US as a routine evaluation is wrong. The study cited to support this (Ding et al) had a much lower prevalence of primary hypertension than would be expected in a primary care population, and even then only 20% had abnormal US. I recommend that you look at Nugent et al, J Pediatrics 2022 which gives a more realistic estimate of the prevalence of primary vs. secondary hypertension in primary care settings and comes down on the side of less testing.

*Response: We tend to agree, and have tried to leave this open (Table 7 footnote – ‘If not routinely, then in young children (<6 years) and those being considered for pharmacotherapy’), noting other responses asking why we have not been more definitive in recommending routine ultrasound. We have updated this section as below:*

*‘This pathway aligns with the AAP 2017 Guideline, aside from the recommendation for routine kidney ultrasound. Accurate data on the diagnostic yield of screening ultrasound are hard to obtain, with studies including children referred to specialist hypertension clinics indicating that contributory ultrasound findings are common among children without other abnormalities, and that approximately half of children with chronic kidney disease (CKD) do not have proteinuria.[61, 66] Yet, among 321 children enrolled in trials of valsartan, renal tract ultrasound abnormalities were uncommon among those ≥6 years of age.[67]’*

Comment: I have similar concerns about recommending echocardiograms for all, albeit for different reasons. And the recommendation to do echo contradicts what you say in Table 1, statement 15

*Response: Given the lack of definitive evidence, we have left some room for practice variation about routine echocardiography and ultrasound. With both recommended ‘If not routinely, then in young children (<6 years) and those being considered for pharmacotherapy’. Table 1, statement 15 is intended to be taken in the context of Table 1, statement 14.*

## 16.6 Cardiac investigations; Recommendations 13-16

Comment: The recommendation against routine ECG for LVH aligns with evidence of low sensitivity, whereas echocardiography prior to pharmacotherapy is appropriate.

-Indicate that three-dimensional echo or cardiac MRI may be considered in equivocal cases of concentric remodelling.

*Response: Added as suggested to the cardiac investigations section.*

-Add guidance on reporting standards (e.g. Devereux-indexed LV mass) to improve inter-site consistency.

*Response: The advice of our paediatric cardiology expert is that most centres use device-loaded z-scores, to report echocardiography measurements. Rather than dictating an additional calculation for sonographers to perform, our preference is to allow paediatric cardiology departments to maintain currency with regards to best practice normalisation of measures such as LV mass.*

Comment: Suggest highlight checking femoral pulses, radio-tibial/femoral delay, 4 limb BP more critical part of clinical assessment, use of ECG to look for LVH, echo as able to

*Response: ECG is specifically advised against as it has poor accuracy for LVH, this aligns with current evidence, input from our cardiology experts involved in guideline development, and feedback from the Heart Foundation. What data are available on 4-limb BP suggest this too adds little to examination. It is typically normal in children with mid-aortic syndrome. In children with coarctation, most with significant post-ductal BP differentiation present in the neonatal period.*

Comment: I do not believe there is evidence to support the definitive statement “Children with a normal nocturnal BP and dipping pattern are unlikely to have a secondary hypertension.”

Nondipping is less common in primary hypertension (based on limited studies), however as noted in ref 70, 11% of children with primary hypertension were classified as nondippers.

*Response: This sentence does not relate to the use of non-dipping to identify children at high-risk of secondary causes. It states that children with a normal nocturnal BP and dipping pattern represent a cohort at low-risk of secondary causes.*

Comment: Is it worth a statement that ECG does not have a role in screening for LVH. It is in a table somewhere, but not in this section of text and when reading I was left wondering why wouldn’t just do an ECG to screen (as not always easy to get an ECHO)

*Response: Added to discussion:*

*‘The relevant AAP statements were adopted (including that ECG is not a useful test to screen for LVH), except for 15-2 regarding the definition of LVH.[69]’*

Comment: Is there any suggestion if there is no or limited access to paediatric ECHO? Is there anything else that can be done to investigate for LVH? Or is paediatric ECHO widely available in the community across Australia.

*Response: Paediatric echocardiography is relatively widely available in rural and remote Australia due to the high incidence of rheumatic heart disease.*

Comment: Statement on diagnosis of HT in children and adolescents and need for paediatric review - to do with investigations P12 P13 Echo has been mentioned many times but no statement of appropriateness of who should perform this and are there medicare issues for this?

*Response: There is an unrestricted MBS item for paediatric echocardiography reported by a cardiologist.*

## 16.7 When and how to investigate for a renovascular cause; Recommendations 17-18; Table 8: Potential indications for further imaging after a normal renal ultrasound

Comment: The manuscript sensibly reserves CT angiography/MRA for selected cases after a normal renal ultrasound. Table 8 gives practical red-flag criteria.

-Specify preferred first-line imaging modality (Doppler vs contrast-enhanced ultrasound) in centres without paediatric CT/MRA.

-Emphasise renal-functional considerations when using contrast in young children.

-Another section on When and how to investigate for an endocrine cause?

-Additionally, you could highlight that prevalence of diabetes is increasing in youth and management of hypertension in this population is going to be increasingly important.

*Response: The manuscript is intended to advise against the use of CT/MRA for renovascular disease. In children, the size of the arteries means these techniques lack sufficient point-point resolution to rule out or even accurately delineate disease in many cases. This means that negative imaging does not rule out renovascular disease, and positive imaging requires an angiogram for confirmation of diagnosis and intervention.*

*‘Cross sectional imaging modalities, such as computed tomography (CT) and magnetic resonance imaging (MRI), have a role in the investigation of renovascular disease. However, differences in aetiology, the distribution of disease and vessel size mean cross sectional imaging remains less sensitive as a diagnostic test in children compared to adults.[70, 71] Hence, direct angiography is still required when there is a high index of suspicion for renovascular disease.[65, 72] There are specific situations in which CT or MRI can be helpful (e.g. monitoring disease progression, in larger patients with a low pre-test probability, MRI to assess vessel wall involvement in vasculitis) and, in centres without access to direct angiography, they are more likely to form part of the diagnostic work-up.[70, 73]’*

*Endocrine causes TSH/cortisol/metanephrines/renin and aldosterone are included in the table of investigations for young children, those with severe or resistant hypertension. There have been some questions from non-paediatric doctors about primary aldosteronism, which is understandable give this is an expanding area of research and understanding. However, it remains uncommon in children and more commonly relates to monogenic causes of hypertension. The following has been added to clarify:*

*‘The AAP 2017 and ESH 2016/2023 Guidelines recommended the removal of routine renin and aldosterone testing for children with hypertension at low-risk of secondary causes. These tests have a low yield in even among children presenting to tertiary hypertension clinics and poorly predict undetected renovascular disease.[60-62] Primary aldosteronism is an area of active research; however, data indicate continuum of aldosterone dysregulation and diagnostic thresholds vary widely.[63, 64] Measurement of systemic renin and aldosterone remain part of screening for young children or those with severe hypertension. Low renin values should prompt investigation for monogenic disorders of hypertension, all of which are associated with low renin aside from autosomal dominant hypertension with brachydactyly.[65]’*

Comment: Also add: slow to weight gain (poor growth/FTT), hyponatremia, polyuria/polydipsia and nephrotic range proteinuria to criteria to Ix for renovascular cause

*Response: These non-specific features may also suggest other potential causes of secondary hypertension such as renal dysplasia.*

P14 Back to dipstick - this has been counselled against for some time now and need to highlight proteinuria not just microalbuminuria - this needs to be highlighted

*Response: Dipstick protein on a concentrated sample (any other than SG <1.005) is sufficient to rule out proteinuria for the purpose of screening for CKD. This is discussed in the relevant section. A review covering this topic in more detail is available for further information (Larkins NG, Teixeira-Pinto A, and Craig JC. A narrative review of proteinuria and albuminuria as clinical biomarkers in children. J Paediatr Child Health 2019;2:136-142).*

Comment: “the overall sensitivity for ultrasound remains low (73%)…” I would point out here that the study from GOSH (your ref 77) showed higher sensitivity of CTA than Doppler US and CT may reveal other abnormalities missed on US.

*Response: This is true. However, CT remains insufficiently sensitive to exclude renovascular disease in patients with a high index of suspicion without clear benefit. In some cases MRI can be useful to provide imaging of the vessel wall, in combination with direct angiography.*

## 16.8 Albuminuria as a routine test; Recommendation 19

Comment: Routine albuminuria screening is well justified given its prognostic value in primary and secondary hypertension. State the recommended specimen (early-morning spot ACR) and frequency (annually unless CKD present).

*Response: Albuminuria does not have prognostic value in paediatric hypertension. In children and adolescents with essential hypertension alone, it is almost never observed. Australian studies and a review discussing this in more detail are included (Larkins NG, Teixeira-Pinto A, and Craig JC. A narrative review of proteinuria and albuminuria as clinical biomarkers in children. J Paediatr Child Health 2019;2:136-142). However, to avoid conflict with adult kidney health recommendations we have amended the guideline to include albuminuria as an acceptable test, noting the 20% false positive rate on erect spot samples.*

Comment: The wording in the guidelines around the role of protein on dipstick being the preferred screening tool and why urine albumin is less accurate in children is unclear. The recommendation not to screen children with hypertension for microalbuminuria unless they have chronic kidney disease (CKD) could be misleading. The wording may imply that most cases of childhood CKD will be identified through opportunistic dipstick testing and blood pressure checks, which may not be the case.

*Response: We appreciate the feedback regarding clarity of urine protein testing recommendations. The wording of the relevant Key Action Statement has been amended as follows:*

*“For children and adolescents with primary hypertension, testing for total protein by dipstick or protein-to-creatinine ratio is recommended rather than microalbuminuria.”*

*The intention is to highlight that microalbuminuria is not an appropriate screening test in children, given that the aetiology of childhood kidney disease differs from adult populations (dysplasia and tubular disease being more common), and the exaggerated orthostatic variation in albumin excretion among older children and adolescents. The guidelines do not recommend using dipstick testing to exclude CKD. This is clarified in the Investigations section and relevant table, which recommend a serum creatinine for all children with confirmed hypertension and renal tract ultrasound for most children. However, to avoid conflict with adult kidney health recommendations we have amended the guideline to include albuminuria as an acceptable test, noting the 20% false positive rate on erect spot samples.*

Comment: This is at odds with the routine CKD screening in HT ie that GPs have been informed ie ACR and not to use dipsticks. This has been counselled against for some time now and need to highlight proteinuria not just microalbuminuria - this needs to be highlighted.

*Response: See above. There has been a large effort for GPs treating adult patients but this is very different to treating children, given the very different causes of CKD (diabetic and hypertensive nephropathy are rare in childhood), and lack of certainty about the importance of low-level albuminuria in children. Australian data demonstrate that applying adult methodology (random spot uACR) to children results in 20% being diagnosed with albuminuria. Consistent with this, one of the most common referrals to paediatric nephrology is benign orthostatic proteinuria detected on uACR. However, to avoid conflict with adult kidney health recommendations we have amended the guideline to include albuminuria as an acceptable test, noting the 20% false positive rate on erect spot samples.*

Suggest consider a broader scope for this paragraph that includes recommendations for the timing and role of routine monitoring for proteinuria, rather than differences between measurement methods. i.e. timing and frequency of screening recommended for stage 1/2 populations using either UA/UPCr

Whilst there is understanding of the difference in need for measurement of total urinary protein rather than Microalbuminuria in the paediatric population, we would like the guideline to highlight more clearly in table 2 the reasons why the Urinary ACR, which is now a guideline directed screening for CKD in those over 18 years of age, is not the initial test required for children when screening for CKD. Our education and guidelines for the primary care sector have focused on moving away from using urine dipstick to assess proteinuria and we want to ensure that this does not confuse the messaging. A simple statement is suggested below to define the difference: In patients under 18, total protein is preferred over albumin for screening; a concentrated urine dipstick (≥1+) should prompt a protein-to-creatinine ratio, ideally confirmed with a first-morning sample due to common benign orthostatic proteinuria in adolescents.

*Response: The following change has been made to clarify the Key Action Statement relating to albuminuria based on feedback:*

*‘19. For children and adolescents with primary hypertension, testing for total protein by dipstick or total protein-to-creatinine ratio is recommended rather than microalbuminuria.*

*The main message is that microalbuminuria is not an appropriate test to screen children for kidney disease and that total urinary protein is a more useful test, given the aetiology of kidney disease in children and exaggerated orthostatic variation in protein excretion among children. Australian data confirm that albuminuria is present in 15% of Australian children on a spot urine sample (higher among adolescents). This is discussed in the manuscript and further in the review referenced which delves into this topic in more detail (Larkins NG, Teixeira-Pinto A, and Craig JC. A narrative review of proteinuria and albuminuria as clinical biomarkers in children. J Paediatr Child Health 2019;2:136-142). However, to avoid conflict with adult kidney health recommendations we have amended the guideline to include albuminuria as an acceptable test, noting the 20% false positive rate on erect spot samples.*

## 16.9 Treatment targets in the general paediatric population; Recommendation 19

Comment: A target < 90th percentile or < 130/80 mmHg is consistent with adult guidelines and balances efficacy against over-treatment. Provide a short table showing expected BP reductions with lifestyle versus single-drug therapy to contextualise the target.

*Response: A table has been added to this Supplement reviewing the effects of lifestyle interventions on BP in children and adolescents (Table A4), along with a brief synthesis of the evidence that speak to the expected BP reductions. The BP reduction achieved depends on a range of factors, including physical activity dosage, intervention duration, adherence, baseline levels, and multi-component intervention.*

Comment: An expert recommends defining blood pressure (BP) control in children using a time-based, practical approach—such as requiring multiple valid readings over six months, with all or nearly all below 130/80 mmHg (or below the 90th percentile if age-based). As a pragmatic interim definition, three consecutive readings at target could indicate control. The practice of classifying control based on a single reading should be explicitly discouraged, as it contributes to persistent underestimation of true control rates. Due to intra-individual BP variability, sustained control below 130/80 mmHg generally requires an average systolic BP closer to 120 mmHg over time.

*Response: We agree and have added the following text to the manuscript to clarify:*

*‘The panel endorsed the recommendation to target a BP <90^th^ percentile when treating children and adolescents with hypertension. While there is limited evidence to support specific treatment targets in young people, this level of treatment control is usually achievable in clinical practice and aligns with the 130/80 mmHg Roadmap of the National Hypertension Taskforce of Australia for adults.[74] Out of office measurements are preferred (see measurement of BP section). However, for clinical values, a time-average approach is also recommended, such that if the majority of measurements are below the target value, then control is considered sufficient.’*

## 16.10 Non-pharmacologic interventions; Recommendation 20; Table 9: Non-pharmacologic therapy

Comment: Table 9 appropriately prioritises DASH-aligned diet, salt reduction and 60 min/day moderate-vigorous activity

- Include culturally adapted examples of low-salt foods and family-based cooking resources.

*Response: This has been considered in the development of the DASH eating guide that we are developing as a resource to accompany the guideline. There will also be QR codes in that guide that will link to further resources, such as Heart Foundation recipes.*

- Add screen-time reduction and sleep hygiene, both of which may influence paediatric BP trajectories.

*Response: Screen time and sleep hygiene have been included in Table 9*

Comment: The DASH diet has been recommended in the guidelines, but there is inadequate evidence to support the DASH diet as a universal intervention in children in Australia, particularly for any populations not of Anglo-European heritage.

*Response: The DASH diet recommendation was based on a dedicated evidence review and expert consensus involving paediatric nutrition specialists. While there are data across diverse populations per the manuscript, we accept the importance of emphasising culturally appropriate dietary advice. We have strengthened the guideline text accordingly:*

*“While data from diverse populations indicate that the principles of the DASH diet are broadly generalisable, healthy and culturally appropriate dietary patterns should remain the priority. Referral to a dietician is recommended to provide personalised and culturally tailored advice.”*

*A group of experts, including paediatric dieticians, have developed parent and child resources that will be released with the guideline.*

Comment: From our consumer expert: I am most interested in the recommendations (20 and 21) concerning non pharmacologic interventions in lifestyle, diet and exercise. I was surprised in some ways to learn that WHO recommend 60 minutes of moderate to vigorous exercise per day for children. Whilst I think this may be happening in some households you would have to pry iPads and iPhone out of the hands of many kids where they are used as pacifiers from an early age and run right up to adulthood!

*Response: Yes we strongly agree. Much work needs to be done to encourage children and families to increase physical activity levels.*

Comment: Stage 1 or higher to start pharmacological interventions earlier or if there are other underlying risk factors eg cystic kidney disease

*Response: We are not aware of evidence to support this or other guidelines that take a similar approach.*

Comment: Many families will struggle to engage with multiple health care providers, in part due to financial/logistical barriers. Is it worth also advocating for more community based approaches - ie walk to school, active lifestyle? re terminology - consider use of the term 'activity' vs 'exercise', is it possible to use the term 'activity', rather than 'exercise' in some sections? Activity is a broader term, and may not have the same 'barriers' as the term 'exercise'.

*Response: This is a good point. We have changed the term exercise to physical activity unless referring to data regarding exercise specifically.*

Comment: P15 60min of moderate to vigorous ex (general is 30 min for adults - again needs to be highlighted) as does screen time P16

*Response: We have added to this section:*

*‘Higher levels of physical activity are associated with lower BP independent of diet or dietary interventions, and total physical activity volume may be more important than intensity, although both contribute.[75, 76] Interventions from 30 minutes of aerobic exercise, 3 times per week have demonstrated benefit.[77] Observational data support a reduction in systolic BP with up to 150 minutes of at least moderate vigorous physical activity per week, noting that there are other benefits to more frequent physical activity and the current recommendation for children (5 to 17 years old) generally is to aim for 60 minutes of moderate to vigorous physical activity per day.[78, 79] Item 21 relating to physical activity was adopted, which sets lower minimum targets initially (30-60 minutes, 3-5 times per week), acknowledging that for many children meeting the 60 minute broader recommendations represents a large increase in physical activity and usually best achieved and maintained by incremental improvements. Additionally, it is recommended that children from birth to 2 years not spend time in front of screens, from 2-5 years limit screen time to 1 hour per day, and from 5-17 years limit screen/sedentary time in general.[79, 80]’*

Comment: Consider acknowledging the WHO guidelines for PA for children under the age of five.

Consider acknowledging there is insufficient evidence surrounding the domains of physical activity in terms of effectiveness however the Australian guidelines for PA emphasise the movement does not need to be formal nor organised and can focus on play and sport. Consider acknowledging the recommendations surrounding sleep- especially if sleep hygiene is mentioned. It may be beneficial to discuss the relationship of BP regulation and sleep. If screen time is mentioned then sleep should be considered as another important factor.

*Response: We have adjusted the term exercise to physical activity throughout the manuscript unless referring to data for exercise specifically.*

*Sleep is mentioned but there are concerns with these data regarding the potential for residual and/or unmeasured confounding in the observational associations published. Further research is required to confirm the impact of interventions for sleep on BP.*

Comment: It might be helpful to mention how much BP may be expected to drop with dietary and exercise measures i.e. low salt as a motivating factor? I applaud the inclusion of screen time limits and family centered focus. Any comment on stimulant drinks/caffeine beverages?

*Response: We have added discussion about the impact of caffeinated beverages in the BP measurement section:*

*‘Regarding caffeine intake, current guidelines for BP measurement refer to abstaining from caffeine immediately before up or ≤ 30 minutes prior to measurement.[81, 82] This is a balance between the degree of blood pressure elevation after consumption of a typical caffeinated beverage (2 to 5 mmHg) and practicality of obtaining office BP when patients have not been advised to fast, against peak plasma levels being achieved at ~60 minutes post ingestion and a half-life of caffeine of 4 to 6 hours (less among young children).[83-86] The panel recommended maintaining consistency with standard recommendations, but for confirmatory readings to ensure abstinence from caffeine for 4 to 6 hours prior to measurement.’*

Comment: Recommend mentioning that guidance from a dietitian is needed to implement the DASH diet, and data which mention poor adherence with the DASH diet in adolescents

*Response: Added to the manuscript. There are a group of dieticians working with us to develop resources specific to this manuscript and we agree the time and expertise of a dietician is important to achieve optimal outcomes. The first paragraph of the lifestyle section includes:*

*‘Care within multi-disciplinary teams and/or the engagement of allied health professionals in the community is encouraged, given the importance of these components of care to long-term outcomes.’*

*We have added the following to the paragraph on diet / implementing a DASH diet specifically:*

*‘Engaging a dietician as part of the healthcare team is recommended to allow for more detailed assessment of diet than is usually possible in a standard medical appointment and to provide personalised, culturally appropriate advice.’*

Any data available that mention parental adherence to DASH/other dietary interventions and uptake by their children?

*Response: These are included in the lifestyle section:*

*‘Family-centred interventions to improve BP and cardiovascular risk are typically more effective than those that target children alone. Family-centred counselling may involve a dietitian and/or exercise physiologist, and techniques to promote positive and sustainable behaviour change (e.g. motivational interviewing).’*

## 16.11 Pharmacologic management" (page 16)- Recommendation 21 (Table 2) - Table 10: Criteria for pharmacologic antihypertensive therapy

Comment: First-line ACEi/ARB or long-acting CCB is consistent with international practice; Table 10 criteria for drug initiation are thorough. Include a dose-conversion appendix for liquid formulations commonly used in younger children.

*Response: Dose-conversion for liquid formulations is now included in Table A10.*

Comment: If no FBE prior to starting ACE/ARB hard to know if preexisting neutropenia

*Response: Neutropaenia due to ACEi/ARB are rare in children and it is hard to justify universal testing based on this. If neutropaenia did occur and there were no pre-existing FBE then the agent would be ceased to assess causality .*

Comment: Any guidance on de-prescribing? Easy to start, always harder to stop and non-specialists would benefit from knowing what triggers consideration of withdrawal? and if withdrawing anti-HT, how one does that.

*Response:* We have added the following to the section on pharmacotherapy:

‘Children with essential hypertension are likely to need ongoing treatment unless there is an improvement in concurrent risk factors, for example an improvement in body habitus or intervention for obstructive sleep apnoea. De-prescribing in such situations should occur in a graded fashion, similar to dose escalation, to allow assessment of whether BP control is maintained and to avoid symptoms from rapid relative BP change.’

The considerations for children with secondary hypertension are different depending on the cause and any associated concurrent cardiac or vascular abnormalities. We have not dictated this component as it is self-explanatory and such patients will be cared for by paediatric sub-specialists according to the guidelines.

Comment: ACE/ARB teratogenic and suggestion to use alternative agent in those planning a pregnancy. Many (many!) pregnancies are unplanned - for girls/women starting statins in childhood, national and international FH guidelines suggest that post menarche, recommend a contraceptive. Advice on what contraceptive for those with HT helpful - low oestrogen/progesterone only?

Re choice of agent and recommendations re use of ACE/ARB - many pregnancies are unplanned, and there may be specific issues with unplanned teenage pregnancies re timely recognition/access to medical care, so maybe there should be some caveats around use of ACE/ARB in young women of reproductive potential - or at least advice to let them know these medications are not recommended in pregnancy

*Response: We have added clarification to the relevant section:*

*‘The teratogenicity of ACEi/ARB on fetal kidney development is well described. Advice about the use of contraception, and using an alternate anti-hypertensive agent if planning a pregnancy or when a pregnancy is confirmed, are essential components of care.’*

Comment: Advising females taking ACEi/ARBs to use reliable contraception should be included.

*Response: Relevant section updated as follows:*

*‘The teratogenicity of ACEi/ARB on fetal kidney development is well described. Advice about the use of contraception, and using an alternate anti-hypertensive agent if planning a pregnancy or when a pregnancy is confirmed, are essential components of care.’*

Comment: What combination medications exist on the market and what is recommendation (eg ACE/calcium channel blocker, ARB/calcium channel blocker, etc) - or is that the time when we should be directed to seek renal advice (which I think most generalists will be wanting to do)

*Response: The availability of specific single pill combinations is frequently changing. We would recommend using an up-to-date drug handbook. However, realistically and per the advice on referral of children or adolescents with hypertension not controlled on ≥2 agents, these children will mostly be referred for specialist care.*

Comment: Is there an Australian reference source for dosing information for the paediatric population for BP meds?

*Response: This information is now provided in Table A10 of this Supplement.*

Comment: Page 18 re comment re evidence for low-dose combination medication. What is the nature of the combination BP meds available in Australia at the moment, are these low-dose? This is important for practitioners to understand, in their application of evidence from studies of low-dose combinations.

*Response: We agree that there are challenges in the Australian context and the below discussion is included in the pharmacotherapy section:*

*‘The Roadmap of the Hypertension Taskforce of Australia endorses the use of single-pill combinations but notes that the regulatory framework in Australia would need to change to facilitate these as first-line treatment. An additional barrier in children is a lack of direct evidence for this approach. Therefore, the panel endorsed the AAP 2017 recommendation to start with a low-dose single agent when pharmacotherapy is required. When treating older children with essential hypertension, consideration should be given to adding a second agent rather than single agent dose escalation when required.’*

Comment: No discussion of need for specialist review prior to starting medication!

*Response: This is deliberate based on the input of the general and subspecialist paediatricians involved in developing these guidelines. The recommendation was made given the waitlist for specialist review is typically over 12-24 months depending on location. General practitioners are also expert in the treatment of primary hypertension and are well placed to provide long-term, family centred care for adolescents with hypertension. This is discussed in the implementation section of the manuscript. Guidance about risk factors defining high-risk populations that should prompt referral for specialist investigations or management are given in the relevant sections.*

Comment: Maybe a brief comment about the status of novel therapies, as many providers and well resourced families may want to use or request these and it would be helpful to have a point of reference. i.e. limited evidence, not approved, we don’t recommend routine use at this time

*Response: Added comment to the translation of novel therapies to children:*

*‘Novel therapies have emerged for the treatment of resistant hypertension.[87] As with any new pharmacotherapeutic agent caution is required with initial use in children to ensure there are no age-dependent side effects, effect modification, and that differences in pharmacokinetics are considered. As such, these are initially in the setting of clinical trials or severe, multi-agent resistant hypertension.’*

Comment: “An additional barrier in children is a lack of direct evidence for [single pill combinations].” Well there is one trial- Sorof JM, Cargo P, Graepel J, Humphrey D, King E, Rolf C, Cunningham RJ. Beta-blocker/thiazide combination for treatment of hypertensive children: a randomized double-blind, placebo-controlled trial. Pediatr Nephrol. 2002 May;17(5):345-50. PMID: 12042891.

*Response: Thank you. This sentence has been updated accordingly:*

*‘An additional barrier in children is a lack of direct evidence for this approach, aside from a 12-week randomised, blinded, placebo-controlled trial of bisoprolol-hydrochlorothiazide.[88] ’*

## 16.12 The role of ABPM in assessing treatment efficacy; Recommendation 22

Comment: Use of ABPM when clinic or home readings are discordant is evidence-based and should improve target attainment

Provide guidance on repeating ABPM 3–6 months after dosage adjustment to confirm control.

*Response: A treatment flowchart has been added to further highlight the role of ABPM. Regarding the frequency of repeat measurement, there were divergent opinions and practice among the experts on the panel and steering committee. The reproducibility and physiologic variability of ABPM in children is an area of active research and we would hope to provide more definitive advice in the future. We agree that in patients with uncertainty or discordance between measurement methods during the initial workup require repeat ABPM and have added the following to the relevant discussion:*

*‘Regardless, ABPM remains the reference standard and when there are differences between measurement methods and should be repeated after 3 to 6 months to confirm treatment adequacy when there is uncertainty about the reliability of other methods in a given patient.’*

Comment: Re ABPM - how do children fit with the current MBS thresholds for eligibility for an ABPM in Australia? The MBS item was developed for diagnosis of HT in adults, so it is possible children with BPs of concern may not meet the thresholds in the MBS item number, so I'm wondering if this may be a potential barrier to MBS supported ABPM.

Good to know but would like a sister guide specifically targeting primary care with simplified guideline, and approaches that will work in primary care. Access to appropriate ABPM may be problematic - will standards be developed and enforced. I am unsure of the capacity for pathology companies to do this in children.

*Response: A flowchart has been added to improve ease of implementation. A sister guide is planned for general practitioners with release.*

*We have made a submission to MSAC and are awaiting determination about the inclusion of paediatric thresholds that would allow children to access ABPM. There is not currently capacity that we are aware of, but we hope to work with providers once final feedback from MSAC has been received.*

*The US guideline ABPM update referenced and endorsed is effectively the current global standard with methods for performing and interpreting 24-hour ABPM.*

## 16.13 Treatment of children with hypertension and kidney disease; Recommendations 23-27

Comment: The strong recommendations for ACEi/ARB, < 50th percentile ABPM targets and regular proteinuria assessment are well supported. Specify a pragmatic ABPM frequency (e.g., 6-monthly while uncontrolled, 12-monthly once stable).

*Response: Substantial clinical practice variation was unearthed during voting on this item. Some units are performing ABPM annually and others rarely. See earlier discussion about ABPM generally. However, we have left this open as children with CKD are treated by subspecialists who are capable of continuing to make the determination in-lieu of definitive evidence.*

Comment: For diabetes (Rec 28) the threshold aligns with adult criteria but differs from ISH youth guidance; briefly justify choice to pre-empt confusion.

*Response: We are not aware of ISH youth guidance but the (adult) ISH 2020 Guidelines support a threshold of 140/90 mmHg for treatment initiation. The ISPAD microvascular complications guideline follows the AAP 2017 Guideline rather than supporting a lower threshold. We added to the manuscript highlight the latter to avoid confusion.*

*‘Currently, there are a lack of data supporting a lower treatment initiation threshold in diabetes mellitus compared to the general population; noting the International Society for Paediatric and Adolescent Diabetes (ISPAD) Consensus Guideline follows the AAP 2017 threshold of 130/80 mmHg.[89, 90]’*

Comment: “Hence, in children with CKD, BP is measured at every medical encounter and a target BP ≤50th percentile is recommended.” This needs to be edited to clarify that ESCAPE targeted a 24-hr MAP of <50th percentile, not office BP. 50th percentile of office BP is a different number than 50th percentile of MAP. I think many papers confuse this.

*Response: This is noted and there was vigorous debate about BP targets by the panel. We amended the relevant section in the table but not the manuscript text. The target in the table reads ‘Children or adolescents with both chronic kidney disease and hypertension should be treated to lower 24-hr mean arterial pressure <50th percentile by ambulatory BP monitoring. In-lieu of recent ambulatory BP data, a target office BP of the 50th percentile (acceptable range 25^th^ to 75^th^ percentile) measured by auscultation may substitute’. The following has been added to the relevant manuscript discussion:*

*‘The lack of difference in primary outcome left in HOT-KID leaves uncertainty about the net benefit of treating to SBP by auscultatory, office BP. The panel recommended that the treatment target should be to lower 24-hr mean arterial pressure <50th percentile by ambulatory BP monitoring. In-lieu of recent ambulatory BP data, a target office BP of the 50th percentile (acceptable range 25^th^ to 75^th^ percentile) measured by auscultation may substitute.’*

## 16.14 Treatment of children with hypertension and diabetes mellitus; Recommendation 28

Comment: This section is about children with DM. Do you recommend waiting until children with DM have albuminuria before initiating RAAS blockade? I think that would be unfortunate.

*Response: We agree. We have revisited this section and amended.*

## 16.15 Treatment of acute severe hypertension; Recommendation 29

Comment: The directive to reduce BP by ≤ 25 % of the planned reduction in the first 8 h matches international consensus and is appropriately graded ‘expert opinion’. Provide an emergency drug table (doses of labetalol, hydralazine, sodium nitroprusside) in the supplement to assist rural centres.

*Response: This information is now provided in Table A11 of this Supplement.*

Comment: Should also treat moderate hypertension

*Response: This is the intention. We had added a flowchart to make the timing of treatment initiation easier.*

Comment: There is also no adult guideline. Examination of the optic fundi is essential, and can be achieved via an optometry review, which should be fairly widely available in the community, and should be straight forward for slightly older children who can sit and co-operate, although may be more difficult in toddlers and babies. Hypertensive crisis is NOT defined by BP levels, as it depends on the rate of rise of BP as well as the level. It is also defined by clinical findings, and clinical assessment in combination with BP, NOT by BP levels alone. Maybe need to split this section to talk separately about hypertensive crisis vs severe hypertension.

*Response: We have added reference to the importance of considering rate of rise in BP in the relevant table and directed readers to ensure the section on acute severe hypertension is not overlooked. Clarifications to the manuscript text as follows:*

*‘The AAP definition of severe hypertension was endorsed (95^th^ percentile + 30 mmHg) to a limit of 180/110 mmHg per the National Heart Foundation of Australia 2016 Guideline. However, it is important to note that hypertensive emergencies and the risk of developing clinical sequalae such as posterior reversible encephalopathy syndrome (PRES) are also influenced by the rate of BP rise. PRES is more common among certain patient groups (e.g. kidney disease, including glomerulonephritis, and oncology patients).[91-94] Children with no or minimal symptoms may be treated with oral antihypertensive agents (hypertensive urgency), but those with clear symptoms related to acute hypertension (hypertensive emergency) should be treated in the intensive care unit with intravenous antihypertensive agents.[45, 47, 95] Common symptoms include blurred vision, headache, vomiting and seizures in association with raised intracranial pressure and/or PRES. Hence, a thorough neurologic examination is important, including for papilloedema when possible. Infants tend to present with non-specific symptoms (e.g. lethargy, poor feeding) and/or heart failure.[96-98]’*

Comment: Table 2: Statement 29: The recommendation that the short acting agent should be an IV titratable agent with intensive care monitoring or if not available then treatment should be commenced under consultation with a paediatric hypertension specialist/ paediatric emergency retrieval service.

*Response: The relevant Key Action Statement was adopted from AAP 2017 by the Clinical Advisory Panel after the Delphi process described. The relevant section of the manuscript provides the following discussion, including added reference to a table of dosing based on reviewer feedback:*

*‘The use of intravenous agents when treating patients with symptoms allows for careful titration to ensure a controlled reduction in BP (target: <25% in the first 6 to 8 hours of treatment). A more rapid reduction in BP carries greatest risk in children with longstanding hypertension among whom vascular adaptations may lead to inadequate end organ perfusion (e.g. brain, retina, kidneys) with large drops in BP, regardless of the absolute level of BP.[99] The choice of agent will depend on local preference and medication availability, along with patient-specific factors. Acute severe hypertension and treatment in the Australian context has recently been reviewed; see Table A11 (Online Supplement) for suggested treatment options.[95]’*

## 16.16 Sports participation; Recommendation 30

Comment: The permissive stance, with cardiology review only for significant cardiac changes, avoids unnecessary exclusion while ensuring safety. Include a reference to the Australian Sports Commission’s pre-participation screening tool for consistency.

*Response: The following has been added:*

*‘The AUSactive pre-exercise screening tools provide further advice on broader considerations for exercise among those beginning an exercise program from a sedentary or low baseline, or when significantly upgrading exercise intensity (https://ausactive.org.au/pss-yp).’*

Comment: Consider acknowledging the importance of motor neural development in the sentence- Exclusion from sports participation also has negative impacts upon physical and mental health as childhood is considered a critical window for movement development. It is not just physical health that is impacted, but development of key movements itself and further supports your recommendation.

*Response: Thank you, we have added reference to the importance of childhood as a window for the development of normal motor skills.*

*‘Physical activity in early life specifically is important, being associated with the development of motor skills, and the persistence of physical activity and sports participation among school-aged children as a predictor of physical activity in adulthood.[100-102]’*

## 16.17 Transition of care; Recommendation 31

Comment: Clear guidance to transition by 16–18 y with documented handover supports continuity.

- Recommend co-developed transition plans involving both paediatric and adult teams, especially for complex cases.

From our consumer expert: Also, I had not previously thought of the transition arrangements from child to adult and the changes that this tricky issue would involve. The problems with 1st nations health also made me wonder if that would need very particular attention and even a separate category of recommendations.

*Response: We have added reference co-developed transition plans:*

*‘The principles of good transition, including preparation, communication, co-developed transition plans, and empowering families as healthcare consumers are universal.’*

Comment: No mention of transition clinics, nursing support during transition - many people are lost in transitioning to adult care (T1DM, congenital heart disease), Explicitly involving the GP, stressing importance of follow up. There is no mention of transitioning to an adult physician - surprising given the burden of disease and morbidity that can be associated with poorly treated HT

*Response: Transition of care is a broad topic impacting all children being treated outside of primary care. In children with essential hypertension that is well controlled, we believe that general practitioners are best positioned and sufficiently expert to provided ongoing care. The section that specifically covers transition is as follows:*

*‘This item was adapted to reflect differences in the Australian healthcare system regarding the age of transition, which typically occurs between 16 and 18 years of age. Paediatricians and families strongly believe that transition should be tailored to individual capacity and healthcare status. The principles of good transition, including preparation, communication, co-developed transition plans, and empowering families as healthcare consumers are universal. These actions begin in early adolescence through encouraging the patient voice and fostering positive engagement with health, while supporting a graded increase in patient autonomy and responsibility prior to transfer of care. Setting patients up for lifelong care is one of the reasons why early inclusion of a GP in the care team is important for children with any chronic condition.’*

## 16.18 Specific considerations in a rural and remote context; Table 11: Considerations in Rural and Remote Settings

Comment: Table 11 identifies equipment deficits and workforce shortages; ranking general practice or school programs as highest implementation priority is appropriate.

Add telehealth ABPM interpretation services and mobile outreach clinics to mitigate geographic barriers.

*Response: We are in the early stages of working on ABPM services with MSAC. We intend to continue building and advocating for better access.*

Comment: Any comment on the role of Telehealth to access Nephrology and other subspecialist services?

*Response: Telehealth is well established at all paediatric tertiary hospitals in Australia.*

Comment: “This correlates with higher BP among children in rural and remote Australia compared to those living in urban centres.” This is interesting - why is it the case? Do they have fewer opportunities for aerobic exercise/activity? Is it related to adoption of Westernized diets by Aboriginal populations? Should lifestyle modification be emphasized in these regions? Might be worth expanding on.

*Response: This is an open topic and there are components of all the above. For example, in the Western Desert there is little difference between Aboriginal and non-Aboriginal children, suggesting location related factors such as access to healthy food and safe drinking water. Further research is being conducted, with an MRFF project recently commenced.*

## 16.19 Improving care for Aboriginal and Torres Strait Islander children" (page 23)

Comment: The section rightly highlights higher hypertension prevalence and the importance of cultural safety.

- Recommend co-design of educational materials with Aboriginal Community Health Services.

Please note, based on government advice the preference for terminology that should be used is ‘First Nations people’, ‘Aboriginal and Torres Strait Islander People(s)’, or ‘Indigenous Australians’. Please ensure the term Indigenous is culturally acceptable from your experts.

*Response: We are planning on producing a companion piece to this manuscript, along with culturally appropriate educational materials.*

*The term Indigenous came from a cultural expert. Understandably, there is wide variation around Australia, for example in Western Australia the term First Nations is not usually a preferred term.*

Comment: This was a little light on considering issues that face First Nations people - I thought more should be on screening and models of care - resources such as ACCHOs etc, a home base. Supports and resourcing ACCHOs to do this well is important - many similar programs such as retinal screening, OM etc have worked

*Response: A companion manuscript will be developed to further highlight this question and devote sufficient attention to do these issues justice. This is a grossly under-researched and under-invested area. We are currently collaborating on an Aboriginal led study investigating these questions and attempting to raise awareness among the community, healthcare providers, and policy makers.*

Comment: It may be worth recognising/recommending physical activity that is tied with cultural/historical context or having further consideration about any differences in non-pharmacological interventions that you think would be relevant.

*Response: See earlier responses. Expanding on the cultural and historic context, ongoing resultant healthcare gaps driven by early cardiovascular and kidney disease, and need for a strengths-based approach driven by Aboriginal and Torres Strait Islander people are central to our immediate plans following publication of this guideline.*

## 16.20 Any other parts of the manuscript; Table 1: Summary of key principles from the Australian guideline; The Supplement

Comment: Table 1 (key principles) is concise and aligns well with later Key Action Statements but could signpost where in the document each principle is operationalised.

*Response: Based on this feedback and others we have added a clinical flowchart to the document. This includes page references to the relevant manuscript sections to improve ease of navigation.*

Please note, in the introduction there is the sentence ‘It is aligned in all practical aspects to Heart Foundation, Stroke Foundation, and Hypertension Australia guidelines for blood pressure and cardiovascular disease risk.’ And based on previous feedback above, the Heart Foundation Hypertension guideline is being updated so aspects of this guideline may not be aligned from 2026.

*Response: We have added reference to this in the introduction:*

*‘Noting that the Heart Foundation Guideline is due for review shortly and amendments will be issued to this guideline if significant changes are required to maintain continuity.’*

Comment: I wonder if an adolescent section might be helpful? Can include issues of poor adherence (and strategies to try and improve), overall CVD prevention (not smoking/vaping, ensure lipids normal, healthy weight), esp as the focus is on life course perspective and the ultimate outcome we are trying to avoid is premature CVD in the following decades. Can also provide guidance on most appropriate contraception for young women.

*Response: These are important issues. The principles of adolescent care are applicable to paediatric hypertension but requires a broader discussion than is feasible within the scope of this guideline.*

Comment: Introduction - Should this be Hypertension, or Elevated blood pressure and hypertension?

*Response: We agree this could be posed either way. Given the linear association between BP and risk one could also say any increase in BP above a low-normal value is associated with increased risk. The term hypertension has been used in the introduction to keep the message simple, but we hope the concept that the relationship between hypertension and risk is continuous in a physiologic sense has been conveyed in the discussion about thresholds.*

Comment: Rationale for adapting the AAP 2017 guideline - should this be more generic - Rationale for choice of guideline for adaptation?

*Response: Suggested change made.*

Comment: Supplement 7 Definition of a hypertensive crisis Australia 2025. A clinical hypertensive crisis is NOT defined by an arbitrary BP level, as it depends on both the BP level and the rate of rise. It can occur at relatively low levels of BP, if pre-existing BP is low, and the rate of rise has been rapid. It is recognised and defined by the clinical assessment, in combination with BP readings. I am not sure what you are intending with this box, whether you are meaning to indicate something else (other than a hypertensive crisis). As it is written at the moment, this information is potentially misleading to clinicians, and may contribute to impairing the capacity of clinicians to recognise a hypertensive crisis. Suggest this section requires revision.

*Response: We have amended the relevant section to make this clearer and added a footnote to Table 4. Additionally, a management flowchart has been added to aid providers in identifying the appropriate acuity of care.*

*Clinical hypertensive crisis introduces another term for this area that clinicians already find confusing. The previous definition in the paediatric literature was a hypertensive crisis, divided into urgencies and emergencies based on clinical symptoms. More recently there has been a more towards talking about acute severe hypertension, which captures the key feature of the condition (acute), and then this is divided into the self-explanatory groups with or without symptoms.*

Comment: Consider including a table on red flags on history and examination of the child with hypertension to be able to guide primary care provider in terms of further investigations and referral. This is keeping in mind the differences in the level of experience amongst primary care providers in managing children and time constraints.

*Response: Red flags on history and examination have been included in the added clinical flowchart.*

Comment: Caffeine consumption typically has a longer half life than 30 minutes. Why is the recommendation to avoid it 30 min prior to measurement if it typically has peak BP raising affects ~ 1 hour post consumption and can an affect for ~6 hours post consumption? Exercise is logical with it taking ~30 min to return to resting post exercise, but why is caffeine consumption paired with exercise for this point?

*Response: This is an interesting question. The timing of 30 minutes was taken from the AAP Guideline. An Australian RCT measured BP 1-hour post one cup of coffee in young adults, and found a 2.77 mmHg mean increase in systolic BP between groups (p=0.05) and 2.11 mmHg mean increase in diastolic BP (P = 0.64) [Teng et al 2016]. Of course, the quantity of caffeine ingested will also impact the degree and duration of BP elevation and the pharmacokinetic parameters are not consistent with recommendation.*

*In terms of other major guidelines, the 2021 ‘European Society of Hypertension practice guidelines for office and out-of-office blood pressure’ also recommend 30-minutes abstinence from caffeine prior to measurement [103]. The ‘International Consensus on Standardized Clinic Blood Pressure Measurement’ suggests not measuring BP immediately after caffeine intake [104].*

*For practicality, we have maintained consistency with the adult guidelines for initial measurement, but noted the pharmacokinetic parameters in the manuscript and added a footnote to the table suggesting that confirmatory measurements after taken 4 to 6 hours post-caffeine.*

*‘Regarding caffeine intake, current guidelines for BP measurement refer to abstaining from caffeine immediately before up or ≤ 30 minutes prior to measurement.[81, 82] This is a balance between the degree of blood pressure elevation after consumption of a typical caffeinated beverage (2 to 5 mmHg) and practicality of obtaining office BP when patients have not been advised to fast, against peak plasma levels being achieved at ~60 minutes post ingestion and a half-life of caffeine of 4 to 6 hours (less among young children).[83-86] The panel recommended maintaining consistency with standard recommendations, but for confirmatory readings to ensure abstinence from caffeine for 4 to 6 hours prior to measurement.’*

Comment: Introduction: “Yet, in Australia there is no unified guideline…” You do get to this point later, but it may be relevant to mention here that BP measurement in children is not routinely performed in Australia

*Response: Added for international context:*

*‘Use of international guidelines may not account for the unique characteristics of the Australian population and healthcare system, including geographic distribution and limited person-level preventative healthcare mechanisms once children commence school.’*

Comment: Rationale for adapting the AAP 2017 guideline: “noting that ESH 2023 is primarily an adult-focused guideline and relies on the ESH 2016 guidelines.” Suggest replacing “relies on” with “reproduces verbatim”

*Response: Amended to ‘mirrors’.*

Comment: Did you look at the European Society of Cardiology pediatric GL published in 2022? I think it is an important one, especially as it gives rationale for adoption of the normative data in the 2017 AAP GL and not the Fourth Report data

*Response: We did look at this consensus document and have elaborated on the discussion about normative values per earlier comment.*

Comment: This is a fabulous guideline, comprehensive yet remaining very clear and pragmatic and it will be a great help for paediatricians, GPs and other paediatric health care providers to try and improve detection and management of HT in childhood. CVD prevention is a paediatric issue, but too often gets left in the too hard basket, as the outcome (a CV event) occurs many decades later. It is time policy makers and others invest in childhood prevention of CVD, so that paediatric health care providers to prevent the preventable, and not leave it to adult providers to play catch-up at much greater cost to individuals and the health care system. I wonder if your lead in to this (or associated manuscripts) might include a "call to arms", as detection of high BP (and other significant and modifiable CVD risk factors) needs to become the responsibility of everyone who provides clinical care for children.

The guide provides useful resources, including a list of risk factors for childhood hypertension, and practical suggestions for defining abnormal blood pressure readings and treatment targets in children identified with hypertension.

Well done to all involved in this great work :)

Nice work

Overall, this is an excellent guideline- congrats!

Reads well - will need a lot of education / support if more is to be done in primary care in this cohort. Will need a pocket guide for GPs to use and will need a lot of education.

Excellent work! Congratulations.

*Thank you!*

# 17 References

[1]	National Heart Foundation of Australia. Guideline for the diagnosis and management of hypertension in adults - 2016. National Heart Foundation of Australia, Melbourne, 2016.

[2]	Commonwealth of Australia as represented by the Department of Health and Aged Care. Australian Guideline for assessing and managing cardiovascular disease risk. 2023.

[3]	Mynard JP, Chaturvedi S, Cheung MMH, et al. A Call for Australian Clinical Practice Guidelines for Paediatric Hypertension. *Heart Lung Circ*. 2023; **32**: 1032–1034.

[4]	Flynn JT, Kaelber DC, Baker-Smith CM, et al. Clinical Practice Guideline for Screening and Management of High Blood Pressure in Children and Adolescents. *Pediatrics*. 2017; **140**: e20171904.

[5]	Lurbe E, Agabiti-Rosei E, Cruickshank JK, et al. 2016 European Society of Hypertension guidelines for the management of high blood pressure in children and adolescents. *J Hypertens*. 2016; **34**: 1887–1920.

[6]	Rabi DM, McBrien KA, Sapir-Pichhadze R, et al. Hypertension Canada’s 2020 comprehensive guidelines for the prevention, diagnosis, risk assessment, and treatment of hypertension in adults and children. *Can J Cardiol*. 2020; **36**: 596–624.

[7]	Umemura S, Arima H, Arima S, et al. The Japanese Society of Hypertension guidelines for the management of hypertension (JSH 2019). *Hypertens Res*. 2019; **42**: 1235–1481.

[8]	The ADAPTE Collaboration. The ADAPTE Process: Resource Toolkit for Guideline Adaptation. Version 2.1. 2010 <https://g-i-n.net/wp-content/uploads/2021/05/ADAPTE-Resource-toolkit-V2.1-March-2010-updated-disclaimer.pdf>.

[9]	Mancia G, Kreutz R, Brunström M, et al. 2023 ESH Guidelines for the management of arterial hypertension The Task Force for the management of arterial hypertension of the European Society of Hypertension: Endorsed by the International Society of Hypertension (ISH) and the European Renal Association (ERA). *J Hypertens*. 2023; **41**: 1874–2071.

[10]	de Simone G, Mancusi C, Hanssen H, et al. Hypertension in children and adolescents: A consensus document from ESC Council on Hypertension, European Association of Preventive Cardiology, European Association of Cardiovascular Imaging, Association of Cardiovascular Nursing &amp; Allied Professions, ESC Council for Cardiology Practice and Association for European Paediatric and Congenital Cardiology. *Eur Heart J*. 2022; **43**: 3290–3301.

[11]	Lurbe E, Mancia G, Calpe J, et al. Joint statement for assessing and managing high blood pressure in children and adolescents: Chapter 1. How to correctly measure blood pressure in children and adolescents. *Frontiers in pediatrics*. 2023; **11**: 457.

[12]	Wühl E, Calpe J, Drożdż D, et al. Joint statement for assessing and managing high blood pressure in children and adolescents: Chapter 2. How to manage high blood pressure in children and adolescents. *Frontiers in pediatrics*. 2023; **11**.

[13]	Malachias MVB, Koch V, Colombo FC, Silva ACS, Guimarães ICB, Nogueira PK. 7th Brazilian Guideline of arterial hypertension: Chapter 10-Hypertension in children and adolescents. *Arq Bras Cardiol*. 2016; **107**: 53–63.

[14]	Royal Children’s Hospital Clinical Practice Guideline. Hypertension in children and adolescents. June 2021 <https://www.rch.org.au/clinicalguide/guideline_index/Hypertension_in_children_and_adolescents/>.

[15]	AGREE Next Steps Consortium (2017). The AGREE II Instrument [Electronic version], Retrieved 24 January 2023. <https://www.agreetrust.org/>.

[16]	Yuan H, Shao W, Ren Y, Jiang M, Kong J, Jing F. Non-linear effect of physical activity intervention on blood pressure in children and adolescents: a systematic review and dose-response meta-analysis. *BMC Public Health*. 2025; **25**: 3366.

[17]	Tozo JVA, Tadiotto MC, Tozo TAA, et al. Effects of different physical exercise programs on blood pressure in overweight children and adolescents: systematic review and meta-analysis. *BMC Pediatr*. 2025; **25**: 252.

[18]	Hernandez-Martinez J, Perez-Carcamo Jn, Melki H, et al. Effects of exergaming on morphological variables, biochemical parameters, and blood pressure in children and adolescents with overweight/obesity: a systematic review with meta-analysis of randomized controlled trials. *Children*. 2024; **12**: 29.

[19]	Pozuelo-Carrascosa DP, Cavero-Redondo I, Herraiz-Adillo A, Diez-Fernandez A, Sanchez-Lopez M, Martinez-Vizcaino V. School-based exercise programs and cardiometabolic risk factors: A meta-analysis. *Pediatrics*. 2018; **142**.

[20]	Hassan MA, Zhou W, Ye M, He H, Gao Z. The effectiveness of physical activity interventions on blood pressure in children and adolescents: A systematic review and network meta-analysis. *Journal of Sport and Health Science*. 2024.

[21]	García-Hermoso A, Alonso-Martinez AM, Ramí­rez-Vélez R, Izquierdo M. Effects of Exercise Intervention on Health-Related Physical Fitness and Blood Pressure in Preschool Children: A Systematic Review and Meta-Analysis of Randomized Controlled Trials. *Sports Med*. 2020; **50**: 187–203.

[22]	Oosterhoff M, Joore M, Ferreira I. The effects of school‐based lifestyle interventions on body mass index and blood pressure: a multivariate multilevel meta‐analysis of randomized controlled trials. *Obes Rev*. 2016; **17**: 1131–1153.

[23]	Lopez-Gil JF, Garcia-Hermoso A, MartÃ­nez-GonzÃ¡lez MÃn, Rodriguez-Artalejo F. Mediterranean diet and cardiometabolic biomarkers in children and adolescents: a systematic review and meta-analysis. *JAMA Network Open*. 2024; **7**: e2421976.

[24]	Farhangi MA, Nikniaz L, Khodarahmi M. Sugar-sweetened beverages increases the risk of hypertension among children and adolescence: a systematic review and dose-response meta-analysis. *J Transl Med*. 2020; **18**: 344.

[25]	Rios-Leyvraz M, Bloetzer C, Chatelan A, et al. Sodium intake and blood pressure in children with clinical conditions: A systematic review with metaâanalysis. *J Clin Hyperten*. 2019; **21**: 118–126.

[26]	Leyvraz M, Chatelan A, da Costa BR, et al. Sodium intake and blood pressure in children and adolescents: a systematic review and meta-analysis of experimental and observational studies. *Int J Epidemiol*. 2018; **47**: 1796–1810.

[27]	Abboud M. Vitamin D supplementation and blood pressure in children and adolescents: a systematic review and meta-analysis. *Nutrients*. 2020; **12**: 1163.

[28]	Alnooh G, Alessa T, Noorwali E, et al. Identification of the Most Suitable Mobile Apps to Support Dietary Approaches to Stop Hypertension (DASH) Diet Self-Management: Systematic Search of App Stores and Content Analysis. *Nutrients*. 2023; **15**: 3476.

[29]	Schaafsma HN, Jantzi HA, Seabrook JA, et al. The impact of smartphone app–based interventions on adolescents’ dietary intake: a systematic review and evaluation of equity factor reporting in intervention studies. *Nutr Rev*. 2023; **82**: 467–486.

[30]	Couch SC, Saelens BE, Khoury PR, et al. Dietary Approaches to Stop Hypertension Dietary Intervention Improves Blood Pressure and Vascular Health in Youth With Elevated Blood Pressure. *Hypertension*. 2021; **77**: 241–251.

[31]	Couch SC, Saelens BE, Levin L, Dart K, Galciglia G, Daniels SR. The efficacy of a clinic-based behavioral nutrition intervention emphasizing a DASH-type diet for adolescents with elevated blood pressure. *J Pediatr*. 2008; **152**: 494–501.

[32]	Krijger JA, Nicolaou M, Nguyen AN, Voortman T, Hutten BA, Vrijkotte TG. Diet quality at age 5-6 and cardiovascular outcomes in preadolescents. *Clin Nutr ESPEN*. 2021; **43**: 506–513.

[33]	Loizou P, Taylor CM, Buckland G. The dietary approaches to stop hypertension (DASH) dietary pattern in childhood in relation to cardiometabolic risk in adolescence and early adulthood in the ALSPAC birth cohort. *Public Health Nutr*. 2024; **27**: e86.

[34]	National High Blood Pressure Education Program. The fourth report on the diagnosis, evaluation, and treatment of high blood pressure in children and adolescents. US Department of Health and Human Services, National Institutes of Health, National Heart, Lung, and Blood Institute, 2005.

[35]	Ogihara T, Kikuchi K, Matsuoka H, et al. The Japanese Society of Hypertension guidelines for the management of hypertension (JSH 2009). *Hypertens Res*. 2009; **32**: 3–107.

[36]	Rosner B, Cook NR, Evans DA, et al. Reproducibility and predictive values of routine blood pressure measurements in children. Comparison with adult values and implications for screening children for elevated blood pressure. *Am J Epidemiol*. 1987; **126**: 1115–1125.

[37]	Theodore RF, Broadbent J, Nagin D, et al. Childhood to Early-Midlife Systolic Blood Pressure Trajectories. *Hypertension*. 2015; **66**: 1108–1115.

[38]	Juhola J, Magnussen CG, Viikari JS, et al. Tracking of serum lipid levels, blood pressure, and body mass index from childhood to adulthood: the Cardiovascular Risk in Young Finns Study. *J Pediatr*. 2011; **159**: 584–590.

[39]	Chen X, Wang Y. Tracking of blood pressure from childhood to adulthood: a systematic review and meta-regression analysis. *Circulation*. 2008; **117**: 3171–3180.

[40]	Jacobs DR, Jr., Woo JG, Sinaiko AR, et al. Childhood Cardiovascular Risk Factors and Adult Cardiovascular Events. *N Engl J Med*. 2022; **386**: 1877–1888.

[41]	Kartiosuo N, Raitakari OT, Juonala M, et al. Cardiovascular Risk Factors in Childhood and Adulthood and Cardiovascular Disease in Middle Age. *JAMA Netw Open*. 2024; **7**: e2418148.

[42]	Haley J, Woo JG, Jacobs DR, Jr., et al. A Clinical Tool to Relate Youth Risk Factors to Adult Cardiovascular Events and Type 2 Diabetes: The International Childhood Cardiovascular Cohort Consortium. *J Pediatr*. 2025; **276**: 114277.

[43]	Robinson CH, Hussain J, Jeyakumar N, et al. Long-Term Cardiovascular Outcomes in Children and Adolescents With Hypertension. *JAMA Pediatr*. 2024; **178**: 688–698.

[44]	Freedman AA, Perak AM, Ernst LM, et al. High Blood Pressure in Childhood and Premature Cardiovascular Disease Mortality. *JAMA*. 2025.

[45]	Flynn JT, Kaelber DC, Baker-Smith CM, et al. Clinical Practice Guideline for Screening and Management of High Blood Pressure in Children and Adolescents. *Pediatrics*. 2017; **140**: e20171904.

[46]	Rosner B, Cook NR, Daniels S, Falkner B. Childhood blood pressure trends and risk factors for high blood pressure: The NHANES experience 1988-2008. *Hypertension*. 2013; **62**: 247–254.

[47]	Lurbe E, Agabiti-Rosei E, Cruickshank JK, et al. 2016 European Society of Hypertension guidelines for the management of high blood pressure in children and adolescents. *J Hypertens*. 2016; **34**: 1887–1920.

[48]	National High Blood Pressure Education Program Working Group on High Blood Pressure in Children and Adolescents. The fourth report on the diagnosis, evaluation, and treatment of high blood pressure in children and adolescents. *Pediatrics*. 2004; **114**: 555–576.

[49]	Hamdani G, Mitsnefes MM, Flynn JT, et al. Pediatric and Adult Ambulatory Blood Pressure Thresholds and Blood Pressure Load as Predictors of Left Ventricular Hypertrophy in Adolescents. *Hypertension*. 2021; **78**: 30–37.

[50]	Goulas I, Farmakis I, Doundoulakis I, et al. Comparison of the 2017 American Academy of Pediatrics with the fourth report and the 2016 European Society of Hypertension guidelines for the diagnosis of hypertension and the detection of left ventricular hypertrophy in children and adolescents: a systematic review and meta-analysis. *J Hypertens*. 2022; **40**: 197–204.

[51]	Stabouli S, Redon J, Lurbe E. Redefining hypertension in children and adolescents: A review of the evidence considered by the European Society of Hypertension and American Academy of Pediatrics guidelines. *J Hypertens*. 2020; **38**: 196–200.

[52]	Larkins NG, Choong C, Schlaich M, et al. Defining childhood hypertension: is it too complicated? An evaluation of the potential impact of different approaches in an Australian paediatric population. *J Hypertens*. 2024; **42**: 1932–1939.

[53]	Wuhl E, Witte K, Soergel M, Mehls O, Schaefer F, German Working Group on Pediatric H. Distribution of 24-h ambulatory blood pressure in children: normalized reference values and role of body dimensions. *J Hypertens*. 2002; **20**: 1995–2007.

[54]	Stergiou GS, Yiannes NG, Rarra VC, Panagiotakos DB. Home blood pressure normalcy in children and adolescents: the Arsakeion School study. *Journal of hypertension*. 2007; **25**: 1375–1379.

[55]	Yip GW, Li AM, So HK, et al. Oscillometric 24-h ambulatory blood pressure reference values in Hong Kong Chinese children and adolescents. *J Hypertens*. 2014; **32**: 606–619.

[56]	Liu H, Zhao D, Sabit A, et al. Arm Position and Blood Pressure Readings: The ARMS Crossover Randomized Clinical Trial. *JAMA Internal Medicine*. 2024; **184**: 1436–1442.

[57]	Stergiou G, Stambolliu E, Bountzona I, et al. Home Blood Pressure Monitoring in Children and Adolescents: Systematic Review of Evidence on Clinical Utility. *Curr Hypertens Rep*. 2019; **21**: 64.

[58]	Stergiou GS, Nasothimiou E, Giovas P, Kapoyiannis A, Vazeou A. Diagnosis of hypertension in children and adolescents based on home versus ambulatory blood pressure monitoring. *Journal of hypertension*. 2008; **26**: 1556–1562.

[59]	Wühl E, Hadtstein C, Mehls O, Schaefer F. Home, clinic, and ambulatory blood pressure monitoring in children with chronic renal failure. *Pediatric research*. 2004; **55**: 492–497.

[60]	Guarino S, Tirelli P, Di Sessa A, et al. Investigations for diagnosis of secondary hypertension in children: yield and costs. *Pediatr Nephrol*. 2025.

[61]	Ding FL, Elias I, Wright R, De Mello A, Cojocaru D, Dionne J. Yield of diagnostic testing in evaluating etiology and end organ effects of pediatric hypertension. *Pediatr Nephrol*. 2023.

[62]	McTaggart SJ, Gulati S, Walker RG, Powell HR, Jones CL. Evaluation and long-term outcome of pediatric renovascular hypertension. *Pediatr Nephrol*. 2000; **14**: 1022–1029.

[63]	Parksook WW, Brown JM, Omata K, et al. The Spectrum of Dysregulated Aldosterone Production: An International Human Physiology Study. *J Clin Endocrinol Metab*. 2024; **109**: 2220–2232.

[64]	Kayser SC, Dekkers T, Groenewoud HJ, et al. Study Heterogeneity and Estimation of Prevalence of Primary Aldosteronism: A Systematic Review and Meta-Regression Analysis. *J Clin Endocrinol Metab*. 2016; **101**: 2826–2835.

[65]	Larkins N, Roebuck D. Diagnostic Evaluation of Pediatric Hypertension. *Pediatric Hypertension*, 2022; 1–17.

[66]	Wong H, Mylrea K, Feber J, Drukker A, Filler G. Prevalence of complications in children with chronic kidney disease according to KDOQI. *Kidney Int*. 2006; **70**: 585–590.

[67]	Flynn J, Zhang Y, Solar-Yohay S, Shi V. Clinical and demographic characteristics of children with hypertension. *Hypertension*. 2012; **60**: 1047–1054.

[68]	Williams KM, Shah AN, Morrison D, Sinha MD. Hypertensive retinopathy in severely hypertensive children: demographic, clinical, and ophthalmoscopic findings from a 30-year British cohort. *J Pediatr Ophthalmol Strabismus*. 2013; **50**: 222–228.

[69]	Grossman A, Prokupetz A, Koren-Morag N, Grossman E, Shamiss A. Comparison of usefulness of Sokolow and Cornell criteria for left ventricular hypertrophy in subjects aged< 20 years versus> 30 years. *The American journal of cardiology*. 2012; **110**: 440–444.

[70]	Vasbinder GB, Nelemans PJ, Kessels AG, et al. Accuracy of computed tomographic angiography and magnetic resonance angiography for diagnosing renal artery stenosis. *Ann Intern Med*. 2004; **141**: 674–682; discussion 682.

[71]	Trautmann A, Roebuck DJ, McLaren CA, Brennan E, Marks SD, Tullus K. Non-invasive imaging cannot replace formal angiography in the diagnosis of renovascular hypertension. *Pediatric nephrology (Berlin, Germany)*. 2016; **32**: 495–502.

[72]	Larkins N, Lurbe E, Flynn JT. Evaluation and Management of Hypertension in Children. *Pediatric Nephrology*, 2021; 1–26.

[73]	Sox HC. Commentary: Accuracy of Computed Tomographic Angiography and Magnetic Resonance Angiography for Diagnosing Renal Artery Stenosis. *Ann Intern Med*. 2004.

[74]	Schutte AE, Bennett B, Chow CK, et al. National Hypertension Taskforce of Australia: a roadmap to achieve 70% blood pressure control in Australia by 2030. *Med J Aust*. 2024.

[75]	Leary SD, Ness AR, Smith GD, et al. Physical activity and blood pressure in childhood: findings from a population-based study. *Hypertension*. 2008; **51**: 92–98.

[76]	Tarp J, Child A, White T, et al. Physical activity intensity, bout-duration, and cardiometabolic risk markers in children and adolescents. *Int J Obes (Lond)*. 2018; **42**: 1639–1650.

[77]	McMurray RG, Harrell JS, Bangdiwala SI, Bradley CB, Deng S, Levine A. A school-based intervention can reduce body fat and blood pressure in young adolescents. *J Adolesc Health*. 2002; **31**: 125–132.

[78]	Sriram K, Mulder HS, Frank HR, et al. The Dose-Response Relationship Between Physical Activity and Cardiometabolic Health in Adolescents. *Am J Prev Med*. 2021; **60**: 95–103.

[79]	Care. AGDoHaA. Physical Activity and Exercise Guidelines for All Australians. . <https://www.health.gov.au/topics/physical-activity-and-exercise/physical-activity-and-exercise-guidelines-for-all-australians>.

[80]	Organization WH. *WHO guidelines on physical activity and sedentary behaviour*. World Health Organization, 2020.

[81]	Cheung AK, Whelton PK, Muntner P, et al. International Consensus on Standardized Clinic Blood Pressure Measurement - A Call to Action. *Am J Med*. 2023; **136**: 438–445 e431.

[82]	Stergiou GS, Palatini P, Parati G, et al. 2021 European Society of Hypertension practice guidelines for office and out-of-office blood pressure measurement. *J Hypertens*. 2021; **39**: 1293–1302.

[83]	White JR, Jr., Padowski JM, Zhong Y, et al. Pharmacokinetic analysis and comparison of caffeine administered rapidly or slowly in coffee chilled or hot versus chilled energy drink in healthy young adults. *Clin Toxicol (Phila)*. 2016; **54**: 308–312.

[84]	Puri BK, Heard CR, Monro JA. Salivary clearance of caffeine in children. *J Oral Biol Craniofac Res*. 2023; **13**: 364–366.

[85]	Savoca MR, MacKey ML, Evans CD, Wilson M, Ludwig DA, Harshfield GA. Association of ambulatory blood pressure and dietary caffeine in adolescents. *Am J Hypertens*. 2005; **18**: 116–120.

[86]	Teng CL, Lim WY, Chua CZ, Teo RS, Lin KT, Yeo JC. Does a single cup of caffeinated drink significantly increase blood pressure in young adults? A randomised controlled trial. *Aust Fam Physician*. 2016; **45**: 65–68.

[87]	Schlaich MP, Bellet M, Weber MA, et al. Dual endothelin antagonist aprocitentan for resistant hypertension (PRECISION): a multicentre, blinded, randomised, parallel-group, phase 3 trial. *Lancet*. 2022; **400**: 1927–1937.

[88]	Sorof JM, Cargo P, Graepel J, et al. Beta-blocker/thiazide combination for treatment of hypertensive children: a randomized double-blind, placebo-controlled trial. *Pediatr Nephrol*. 2002; **17**: 345–350.

[89]	Bjornstad P, Dart A, Donaghue KC, et al. ISPAD Clinical Practice Consensus Guidelines 2022: Microvascular and macrovascular complications in children and adolescents with diabetes. *Pediatr Diabetes*. 2022; **23**: 1432–1450.

[90]	Unger T, Borghi C, Charchar F, et al. 2020 International Society of Hypertension Global Hypertension Practice Guidelines. *Hypertension*. 2020; **75**: 1334–1357.

[91]	Yamamoto H, Natsume J, Kidokoro H, et al. Clinical and neuroimaging findings in children with posterior reversible encephalopathy syndrome. *Eur J Paediatr Neurol*. 2015; **19**: 672–678.

[92]	Fugate JE, Claassen DO, Cloft HJ, Kallmes DF, Kozak OS, Rabinstein AA. Posterior reversible encephalopathy syndrome: associated clinical and radiologic findings. *Mayo Clin Proc*. 2010; **85**: 427–432.

[93]	Siebert E, Spors B, Bohner G, Endres M, Liman TG. Posterior reversible encephalopathy syndrome in children: radiological and clinical findings - a retrospective analysis of a German tertiary care center. *Eur J Paediatr Neurol*. 2013; **17**: 169–175.

[94]	Komur M, Ozgur A, Delibas A, et al. Posterior reversible encephalopathy syndrome (PRES) due to acute hypertension in children: 12 years single-center experience. *Acta Neurol Belg*. 2020.

[95]	Hijazzi S, Moon K, Larkins NG. Oral agents for acute severe hypertension in children with minimal or no symptoms. *J Paediatr Child Health*. 2022; **58**: 1935–1941.

[96]	Lim AM, Chong SL, Ng YH, Chan YH, Lee JH. Epidemiology and Management of Children with Hypertensive Crisis: A Single-Center Experience. *J Pediatr Intensive Care*. 2020; **9**: 45–50.

[97]	Lee GH, Lee IR, Park SJ, Kim JH, Oh JY, Shin JI. Hypertensive crisis in children: an experience in a single tertiary care center in Korea. *Clin Hypertens*. 2015; **22**: 10.

[98]	Yang W-C, Wu H-P. Clinical Analysis of Hypertension in Children Admitted to the Emergency Department. *Pediatrics & Neonatology*. 2010; **51**: 44–51.

[99]	Deal JE, Barratt TM, Dillon MJ. Management of hypertensive emergencies. *Arch Dis Child*. 1992; **67**: 1089–1092.

[100]	Laukkanen A, Pesola A, Havu M, Sääkslahti A, Finni T. Relationship between habitual physical activity and gross motor skills is multifaceted in 5‐to 8‐year‐old children. *Scandinavian journal of medicine & science in sports*. 2014; **24**: e102–e110.

[101]	Cleland V, Dwyer T, Venn A. Which domains of childhood physical activity predict physical activity in adulthood? A 20-year prospective tracking study. *Br J Sports Med*. 2012; **46**: 595–602.

[102]	Telama R, Yang X, Viikari J, Valimaki I, Wanne O, Raitakari O. Physical activity from childhood to adulthood: a 21-year tracking study. *Am J Prev Med*. 2005; **28**: 267–273.

[103]	Stergiou GS, Palatini P, Parati G, et al. 2021 European Society of Hypertension practice guidelines for office and out-of-office blood pressure measurement. *J Hypertens*. 2021; **39**: 1293–1302.

[104]	Cheung AK, Whelton PK, Muntner P, et al. International consensus on standardized clinic blood pressure measurement–a call to action. *The American journal of medicine*. 2023; **136**: 438–445.

1. Declaration of Interest form adapted from that used by the National COVID-19 Clinical Evidence Taskforce
